# Blocking MG53^S255^ Phosphorylation Protects Diabetic Heart From Ischemic Injury

**DOI:** 10.1161/CIRCRESAHA.122.321055

**Published:** 2022-11-07

**Authors:** Fengxiang Lv, Yingfan Wang, Dan Shan, Sile Guo, Gengjia Chen, Li Jin, Wen Zheng, Han Feng, Xiaohu Zeng, Shuo Zhang, Yan Zhang, Xinli Hu, Rui-Ping Xiao

**Affiliations:** 1 State Key Laboratory of Membrane Biology, Institute of Molecular Medicine, College of Future Technology, Peking University, Beijing, China (F.L., Y.W., D.S., S.G., G.C., L.J., W.Z., H.F., X.Z., S.Z., Y.Z., X.H., R.-P.X.).; 2 Peking-Tsinghua Center for Life Sciences, Beijing, China (R.-P.X.).; 3 Beijing City Key Laboratory of Cardiometabolic Molecular Medicine, Peking University, Beijing, China (R.-P.X.).

**Keywords:** cardiac ischemia/reperfusion injury, cardioprotection, GSK3β, insulin resistance, MG53

## Abstract

As an integral component of cell membrane repair machinery, MG53 (mitsugumin 53) is important for cardioprotection induced by ischemia preconditioning and postconditioning. However, it also impairs insulin signaling via its E3 ligase activity-mediated ubiquitination-dependent degradation of IR (insulin receptor) and IRS1 (insulin receptor substrate 1) and its myokine function-induced allosteric blockage of IR. Here, we sought to develop MG53 into a cardioprotection therapy by separating its detrimental metabolic effects from beneficial actions.

**Methods:**

Using immunoprecipitation-mass spectrometry, site-specific mutation, in vitro kinase assay, and in vivo animal studies, we investigated the role of MG53 phosphorylation at serine 255 (S255). In particular, utilizing recombinant proteins and gene knock-in approaches, we evaluated the potential therapeutic effect of MG53-S255A mutant in treating cardiac ischemia/reperfusion injury in diabetic mice.

**Results:**

We identified S255 phosphorylation as a prerequisite for MG53 E3 ligase activity. Furthermore, MG53^S255^ phosphorylation was mediated by GSK3β (glycogen synthase kinase 3 beta) and markedly elevated in the animal models with metabolic disorders. Thus, IR-IRS1-GSK3β-MG53 formed a vicious cycle in the pathogenesis of metabolic disorders where aberrant insulin signaling led to hyper-activation of GSK3β, which in turn, phosphorylated MG53 and enhanced its E3 ligase activity, and further impaired insulin sensitivity. Importantly, S255A mutant eliminated the E3 ligase activity while retained cell protective function of MG53. Consequently, the S255A mutant, but not the wild type MG53, protected the heart against ischemia/reperfusion injury in *db/db* mice with advanced diabetes, although both elicited cardioprotection in normal mice. Moreover, in S255A knock-in mice, S255A mutant also mitigated ischemia/reperfusion-induced myocardial damage in the diabetic setting.

**Conclusions:**

S255 phosphorylation is a biased regulation of MG53 E3 ligase activity. The MG53-S255A mutant provides a promising approach for the treatment of acute myocardial injury, especially in patients with metabolic disorders.

**Novelty and Significance**

**What Is Known?**

- Cardiovascular disease is the leading cause of death in type 2 diabetes (T2D) patients, but the therapeutic options for treating cardiovascular comorbidities are limited.
- MG53 (mitsugumin 53) plays an essential role in cardioprotection against ischemia-induced injury. Meanwhile, it also impairs insulin signaling.

**What New Information Does This Article Contribute?**

- Phosphorylation of S255 by GSK3β (glycogen synthase kinase 3 beta) is required for the interaction of MG53 with its substrates, including IR (insulin receptor) and IRS1 (insulin receptor substrate 1).
- MG53-S255A mutant is E3 ligase-deficient, but retains the cell protective function of MG53.
- In mice with advanced diabetes, recombinant wild type MG53 (rhMG53-WT) fails to elicit protection against cardiac injury caused by ischemia. In contrast, recombinant human MG53-S255A (rhMG53-S255A) protein protects the heart against acute ischemia/reperfusion (I/R) damage as well as I/R-induced myocardial remodeling in the diabetic setting.

There is an unmet medical need in T2D patients for treatment of cardiovascular complications. Many therapeutic strategies are less effective in T2D patients due to their compromised intrinsic protective function and increased vulnerability. On the one hand, MG53 functions as an essential component of the membrane repair machinery and also plays an indispensable role in ischemic preconditioning and postconditioning. On the other hand, MG53 is a negative regulator of insulin signaling, and its metabolic effects rely on the phosphorylation of S255 by GSK3β. We generated a MG53 S255A mutant that successfully separated the cardioprotective function from the detrimental metabolic effects of MG53. Consequently, in the diabetic heart with impaired insulin sensitivity, the therapeutic potential of rhMG53-WT was abrogated by its undesired effects on metabolism, as rhMG53-S255A markedly ameliorated I/R-induced myocardial acute damage and subsequent maladaptive remodeling. rhMG53-S255A may have high therapeutic potential in treating myocardial infarction in patients with diabetes.

**In This Issue, see p 949**

**Meet the First Author, see p 950**

**Editorial, see p 977**

It is well known that people with type 2 diabetes (T2D) are predisposed to heart failure.^1^ In fact, over 60% of T2D patients die of cardiovascular complications.^2,3^ In 2019, American Heart Association published a statement to highlight the internal connection of diabetes with cardiovascular comorbidities and the importance of developing therapeutic strategies to treat both conditions cohesively.^4^ Unfortunately, therapeutic options for treating these comorbidities are limited, because diabetes compromises the cellular protective signaling and increases the myocardial vulnerability toward acute myocardial damage, such as myocardial infarction and ischemia/reperfusion (I/R) injury,^5^ which makes the management of T2D patients experiencing myocardial infarction a big challenge in clinical practice. Thus, it is urgent and pivotal to develop new therapeutic strategies to alleviate T2D-associated acute myocardial damage.

Insulin resistance impairs glycemic control and contributes to the increased incidence of adverse cardiovascular events in T2D patients. Under normal conditions, binding of insulin to IR (insulin receptor) enhances the tyrosine kinase activity of the receptor, which in turn, phosphorylates the tyrosine residue of IRS1 (insulin receptor substrate 1). On the contrary, IRS1 can also be phosphorylated by several S/T (serine/threonine) kinases, such as GSK3β (glycogen synthase kinase 3 beta) activated by insulin signaling,^6,7^ S6K1 by mTORC1,^8^ PKCs by diacylglycerol,^9,10^ and JNK by inflammation.^11^ The S/T phosphorylation of IRS1 suppresses its activation by IR and negatively regulates insulin signaling. Among these S/T kinases, GSK3β is especially important for insulin sensitivity of skeletal muscle.^12^ GSK3β is constitutively active and can be deactivated by insulin-stimulated Akt.^13^ In T2D patients, impaired insulin signaling diminishes Akt-mediated suppression of GSK3β and subsequently enhances the inhibitory phosphorylation of IRS1, thereby exacerbating insulin resistance. In addition, several studies have shown that increased GSK3β activity is accompanied by decreased IRS1 protein abundance in T2D.^14,15^ In particular, it has been shown that in Chinese hamster ovary (CHO) cells, high glucose-induced activation of GSK3 facilitates the ubiquitination and degradation of IRS1.^16^ However, whether similar mechanism works in striated muscle and, if so, which E3 ligase mediates the ubiquitination of IRS1 is still unclear.

MG53 (mitsugumin 53) is a striated muscle-enriched protein with diversified biological targets and functions. It is a major component of cell membrane repair machinery,^17^ and is also essentially involved in cardiac protective signaling triggered by ischemia preconditioning and postconditioning, including reperfusion injury salvage kinase pathway.^18,19^ However, increasing evidence has implicated MG53 as an important negative regulator of insulin sensitivity. Specifically, it targets IR and IRS1 for ubiquitination-dependent degradation via its E3 ubiquitin ligase activity.^20,21^ Moreover, MG53 constitutes a myokine, which binds to the extracellular domain of IR (IR-ECD), allosterically blocking insulin signaling.^22^ Therefore, cardiac protective effect of MG53 could be offset by its detrimental action toward metabolism. Especially in the diabetic heart where insulin sensitivity is seriously disturbed, the therapeutic potential of MG53 is obliterated by its undesired effects on metabolism. To address this challenge, in this study, we have examined the posttranslational modifications of MG53 for solution to separate the 2 sides of the functional roles of MG53. We have found that phosphorylation of serine 255 (S255) by GSK3β enables the E3 ligase activity of MG53 by facilitating its binding to targets. Notably, with the augmented GSK3β activity in the animal models with diabetes or obesity, the level of S255 phosphorylation is significantly elevated, further boosting the detrimental effects of increased MG53 expression under these pathological conditions. Importantly, injection of recombinant human MG53 (rhMG53-WT) in the diabetic *db/db* mice causes a further elevation of blood glucose level, and consequently rhMG53-WT fails to protect the heart against I/R injury in mice with advanced diabetes. In contrast, recombinant human MG53-S255A mutant (rhMG53-S255A), which has no adverse effect of glucose-raising, not only markedly ameliorates I/R-induced acute myocardial damage, but also effectively diminishes chronic maladaptive cardiac remodeling, including myocardial hypertrophy and fibrosis; regardless of the severity of diabetes.

## Methods

### Data Availability

Data supporting finding of this study are available from corresponding authors upon reasonable request. Detailed description of materials and methods can be found in the Supplemental Material. Please see the Major Resources Table in the Supplemental Material.

## Results

### Phosphorylation of MG53 at S255 is Crucial for its E3 Ubiquitin Ligase Activity

To study the posttranslational modifications of MG53, we performed mass spectrometry analysis of the endogenous MG53 from murine skeletal muscle and human MG53 overexpressed in HEK293 cells. Five potential phosphorylated serine residues, serine 2/13, 189, 255, and 307, were identified in human MG53 (Figure S1A and S1B). Of note, phosphorylation of S255 and S307 were also observed in MG53 from mouse skeletal muscle. The 5 serine residues are well conserved across several mammalian species (Figure S1C), implying that they might be critical for the biological functions of MG53.

**Figure 1.**
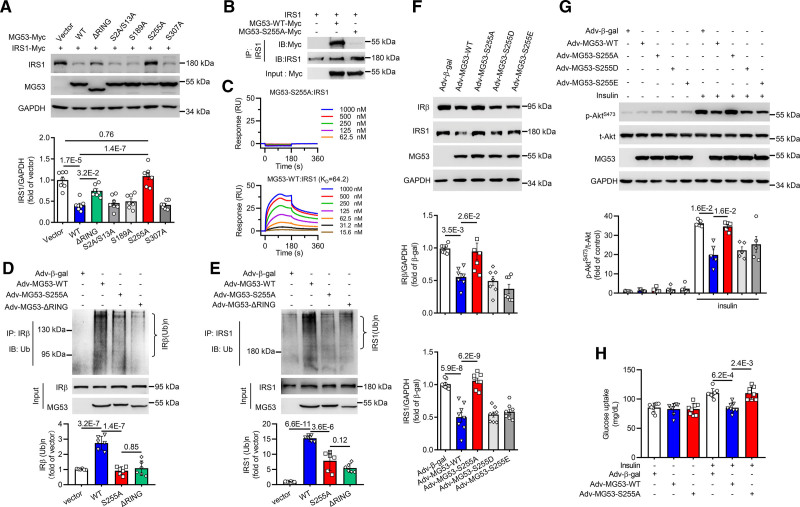
**MG53 phosphorylation at serine 255 is essential for its E3 ligase activity. A**, Representative Western blots and averaged data showing that only S255A mutant abrogated MG53-mediated downregulation of IRS1 in HEK293 cells (n=7). **B**, Co-immunoprecipitation of IRS1 with Myc-tagged wild type MG53 or S255A mutant in HEK293 cells (n=3). **C**, Results of surface plasmon resonance (SPR) assay illustrating S255A mutant abrogated binding of MG53 with IRS1. In this binding assay, IRS1 was immobilized on the chip, MG53-WT or MG53-S255A was injected at indicated concentrations. **D**, **E**, Representative Western blots and averaged data showing the ubiquitination of IRβ (**D**) and IRS1 (**E**) in C2C12 cells expressing wild type, S255A mutant, or ΔRING truncation of MG53 (n=6). **F**, Representative Western blots and averaged data showing S255A mutant abrogated MG53-mediated downregulation of IRβ and IRS1 in neonatal rat ventricular myocytes (NRVMs, n=7 for IRβ, n=8 for IRS1). **G**, Representative Western blots and averaged data showing that S255A mutant abrogated MG53-mediated suppression of insulin-induced phosphorylation of Akt at serine 473 in NRVMs (n=4). **H**, Statistical data showing that S255A mutant abrogated MG53-mediated suppression of glucose uptake in NRVMs (n=8). Normal distribution was confirmed by Shapiro-Wilk test. Data were analyzed using 1-way ANOVA with Tukey post hoc test (**A**, **D**, **E**, and **F** IRS1/GAPDH) and the Mann-Whitney *U* test (**F** IRβ/GAPGH, **G**, and **H**). Data are presented as mean±SEM. IRS1 indicates insulin receptor substrate 1; and MG53, mitsugumin 53.

To explore the significance of these phosphorylation modifications toward the E3 ligase activity of MG53, we individually substituted these serine residues with alanine and overexpressed each mutant or the wild type MG53 (MG53-WT) in HEK293 cells. Since MG53 facilitates the ubiquitination-dependent degradation of IRS1, we co-expressed IRS1 in HEK293 and assessed its protein level as a proxy of the E3 ligase activity. Similar to the situation of the E3-dead truncation lacking the catalytic RING finger domain (MG53-ΔRING), the substitution of S255 with alanine (MG53-S255A) abolished the downregulation of IRS1 mediated by MG53-WT, while other mutations had little effect (Figure 1A). Co-immunoprecipitation (co-IP) showed that MG53-S255A failed to form complex with IRS1 when they were co-expressed in HEK293 cells (Figure 1B). Concomitantly, surface plasmon resonance assay demonstrated that S255A mutation abolished the physical interaction between MG53 and IRS1 (Figure 1C). These results indicate that phosphorylation of S255 is critical for substrate binding to the E3 ligase. Consistently, in C2C12 myotubes, the ubiquitination of IR and IRS1 was enhanced by MG53-WT, but not by MG53-S225A or MG53-ΔRING (Figure 1D and 1E). In fact, compared to the wild type, MG53-S255A markedly attenuated the ubiquitination of IR and IRS1 to the similar extent as MG53-ΔRING that lacks the entire RING domain (Figure 1D and 1E). In contrast, the substitution of S255 with aspartic acid or glutamic acid (MG53-S255D or MG53-S255E) did not interfere with the ubiquitination of the substrates (Figure S2A and S2B). Consequently, overexpression of MG53-S255A or MG53-ΔRING did not reduce the protein abundance of either IR or IRS1 (Figure S2C), while the S255D and S255E mutants could promote the degradation of both substrates as effectively as MG53-WT (Figure 1F). Functionally, overexpression of MG53-WT, MG53-S255D, or MG53-S255E, but not MG53-S255A, impaired insulin signaling, as evidenced by reduced Akt activation (Figure 1G) and glucose uptake (Figure 1H) in response to insulin stimulation in cultured neonatal rat ventricular myocytes. Similar results were obtained for MG53-S255A in C2C12 myotubes (Figure S2C–S2E). Taken together, these data demonstrate that phosphorylation of S255 is critical for the E3 ubiquitin ligase activity of MG53, and that mutation of S255 fully blocks MG53-induced suppression of insulin signaling.

### The Phosphorylation of S255 is Mediated by GSK3β

**Figure 2.**
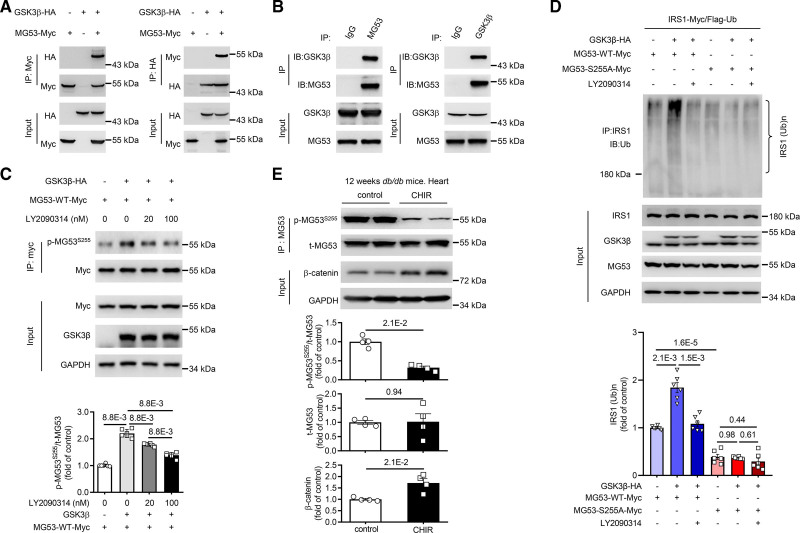
**GSK3β-mediated phosphorylation of MG53 at S255 is crucial for its E3 ligase activity. A**, Co-immunoprecipitation of MG53-Myc and GSK3β-HA when both were expressed in HEK293 cells (n=3). **B**, Co-immunoprecipitation of endogenous MG53 and GSK3β in mouse skeletal muscle (n=3). **C**, Representative Western blots and averaged data showing overexpression of GSK3β enhanced, while GSK3β inhibitor LY2090314 dose dependently attenuated, the level of phosphorylated MG53 at S255 in HEK293 cells (n=6). **D**, Representative Western blots and averaged data showing overexpression of GSK3β enhanced, while GSK3β inhibitor LY2090314 attenuated, IRS1 ubiquitination in HEK293 cells. MG53-S255A could not mediate the ubiquitination of IRS1 (n=6). **E**, Representative Western blots and averaged data showing intraperitoneal injection with CHIR99021, a GSK3β inhibitor, attenuated the phosphorylation of MG53 at S255 in the heart of *db/db* mice (n=4). Normal distribution was confirmed by Shapiro-Wilk test. Data were analyzed using the Mann-Whitney *U* test (**C** and **E**) and 2-way ANOVA with Sidak multiple comparisons test (**D**). Data are presented as mean±SEM. GSK3β indicates glycogen synthase kinase 3 beta; IRS1, insulin receptor substrate 1; and MG53, mitsugumin 53.

We next searched for the kinase that phosphorylates MG53 at S255. To this end, we used NetPhos3.1 (Center for Biological Sequence) to predict the potential kinase(s) for S255 in silico based on the peptide sequence around S255, and identified GSK3 as a top candidate (Figure S3A). We also immunoprecipitated MG53 from mouse skeletal muscle, followed by mass spectrometry analysis of the immunocomplex to identify the kinases that may interact with MG53. Consistently, GSK3β was among the 5 kinases identified by both of the 2 independent immunoprecipitation-mass spectrometry experiments (Figure S3B), as its specific peptides were observed by mass spectrometry (Figure S3C). Indeed, co-IP and reverse co-IP of MG53 and GSK3β co-expressed in HEK293 cells revealed that MG53 and GSK3β were in the same complex (Figure 2A). Their interaction was further confirmed by co-IP of endogenous MG53 and GSK3β from mouse skeletal muscle (Figure 2B). To determine whether GSK3β directly phosphorylates MG53, we utilized in vitro kinase assay where purified MG53-WT or MG53-S255A mutant protein was incubated with GSK3β in the presence or absence of radioactive γ-^32^P-ATP. The phosphorylated MG53 as well as the autophosphorylated GSK3β could be observed when GSK3β and γ-^32^P-ATP were both in the reaction mixture. However, when an inhibitor of GSK3β, CHIR99021, was added, the phosphorylated MG53 or GSK3β was undetectable (Figure S4A). Furthermore, MG53-S255A could not be phosphorylated by GSK3β (Figure S4B), corroborating that the residue phosphorylated by GSK3β is S255. These results strongly suggest that GSK3β is the kinase that phosphorylates MG53 at S255.

To further characterize MG53 S255 phosphorylation and assess its potential physiological and pathological significance, we developed a customized monoclonal antibody (anti-p-MG53^S255^) specifically reacting with phosphorylated MG53 at S255. A series of tests were performed to validate this antibody. First, in HEK293 cells expressing Myc-tagged wild type MG53 or its S255A mutant, this antibody could detect MG53-WT-Myc, but not MG53-S255A-Myc (Figure S5A). Second, pretreatment of MG53-WT protein purified from HEK293 cell lysate with λprotein phosphatase obliterated the band recognized by anti-p-MG53^S255^ antibody (Figure S5B). Third, adding phosphorylated peptide (CLQKILSE-pS-PPPARL) attenuated the signal intensity of S255-phosphorylated MG53 in a dose-dependent manner, while adding the unphosphorylated same peptide (CLQKILSESPPPARL) had no effect (Figure S5C and S5D).

Importantly, the presence of GSK3β not only markedly augmented phosphorylation of MG53 at S255 assessed by this anti-p-MG53^S255^ antibody (Figure 2C), but also robustly increased MG53-mediated ubiquitination of IRS1 (Figure 2D). Another GSK3β inhibitor, LY2090314, dose dependently suppressed GSK3β-mediated increases in S255 phosphorylation as well as the E3 ligase function of MG53 (Figure 2C and 2D). Since MG53-S255A could neither be phosphorylated by GSK3β nor interact with IRS1 (Figure S5A and S5B; Figure 1B and 1C), it failed to mediate IRS1 ubiquitination regardless of the presence of GSK3β (Figure 2D). Thus, the phosphorylation of S255 by GSK3β is necessary for the E3 ligase activity of MG53.

To further confirm the role of GSK3β in vivo, 12-week-old *db/db* mice were treated with CHIR99021. The successful inhibition of GSK3β activity by CHIR99021 was demonstrated by increased protein abundance of β-catenin (Figure 2E). Importantly, with the repression of GSK3β, the phosphorylation of S255 in the heart was markedly reduced, indicating that GSK3β is the major kinase that catalyzing MG53 phosphorylation in vivo (Figure 2E).

### Phosphorylation of MG53 at S255 is Enhanced in Animal Models With Metabolic Diseases

**Figure 3.**
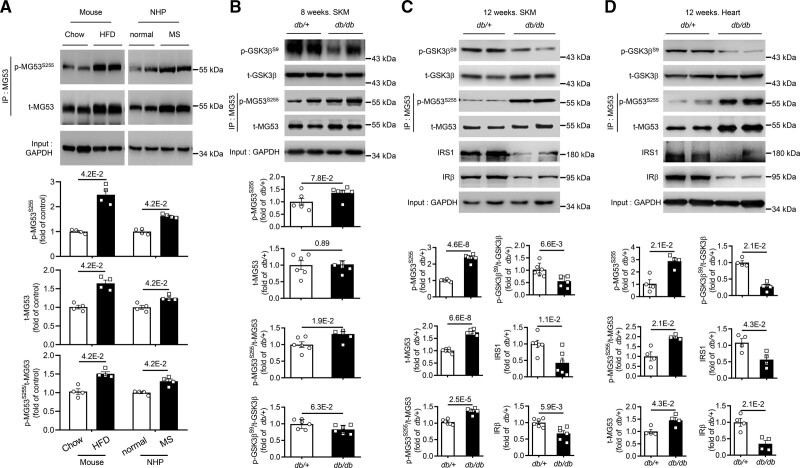
**Phosphorylation of MG53 at S255 is increased in animal models with metabolic diseases. A**, Representative Western blots and averaged data showing increased S255-phosphorylated and total MG53 in the skeletal muscle from high-fat diet (HFD)-induced obese mice and rhesus monkeys with spontaneous metabolic syndrome (MS), versus their respective age- and gender-matched controls (n=4). **B** and **C**, Representative Western blots and averaged data showing repressed phosphorylation of GSK3β at S9, and elevated phosphorylation of S255 and total MG53 in the skeletal muscle (SKM) from 8-week-old (**B**) or 12-week-old (**C**) male diabetic *db/db* mice versus corresponding age-matched controls (n=6). **D**, Representative Western blots and averaged data showing repressed phosphorylation of GSK3β at S9, and elevated phosphorylation of S255 and total MG53 in the hearts from 12-week-old male diabetic *db/db* mice versus corresponding age-matched controls (n=4). Normal distribution was confirmed by Shapiro-Wilk test. Data were analyzed using the Mann-Whitney *U* test (**A**, **B** p-MG53^S255^ and **D**) and 2-tailed unpaired *t* test (**B** t-MG53, p-MG53^S255^/t-MG53, p-GSK3β^S9^/t-GSK3β and **C**). Data are presented as mean±SEM. HSA, human serum albumin; rhMG53-S255A, recombinant human MG53-S255A; and rhMG53-WT, recombinant wild type MG53.

Using anti-p-MG53^S255^, we first assessed the phosphorylation status of MG53 in skeletal muscle of animal models with metabolic disorders, including high-fat diet-induced obese mice and rhesus monkeys with spontaneous metabolic syndrome. Consistent with previous reports, the protein levels of MG53 were significantly increased in the skeletal muscle of the obese mice and metabolic syndrome monkeys (Figure 3A). Importantly, the augmentation of phosphorylated MG53 was even more robust, resulting in an increase in the ratio of S255-phosphorylated to total MG53 (Figure 3A). Similarly, in skeletal muscle of diabetic *db/db* mice, S255 phosphorylation was augmented (*P*=0.078), although total MG53 protein level remained unaltered when the mice were at 8 weeks of age (Figure 3B). The increase in S255 phosphorylation became more prominent with the progression of diabetes (Figure 3B and 3C). It is noteworthy that the enhanced S255 phosphorylation was associated with elevated GSK3β activation, as indicated by decreased phosphorylation of the kinase, and that both MG53 and GSK3β protein levels were significantly upregulated in mice with advanced diabetes (Figure 3B and 3C). The increased activation of GSK3β and MG53-S255 phosphorylation were accompanied by markedly reduced IR and IRS1 protein abundance (Figure 3C). Similar observations were made in the heart of 12-week-old diabetic *db/db* mice (Figure 3D). These results indicate that, under the pathological conditions of T2D, GSK3β-MG53-IR/IRS1 signaling axis forms a vicious cycle where insulin resistance relieves insulin induced-repression of GSK3β, resulting in enhanced phosphorylation of MG53 at S255 and subsequently promoting the degradation of both IR and IRS1, thus exacerbating insulin resistance in the heart and skeletal muscle.

### Mutation of S255 to Alanine Does not Disrupt its Cell Protective Function

**Figure 4.**
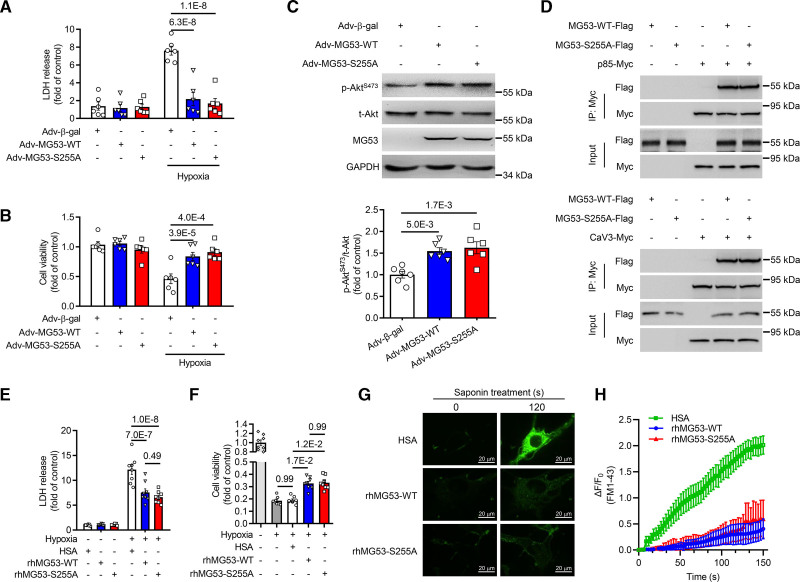
**E3 ligase-deficient mutant MG53-S255A protects cardiac myocytes from hypoxia-induced cell death via activation of PI3K-Akt mediated reperfusion injury salvage kinase (RISK) pathway. A** and **B**, Cell viability of neonatal rat ventricular myocytes (NRVMs) assessed by LDH release in the medium (**A**) and cellular ATP content (**B**). Cells were infected with adenovirus expressing β-gal, MG53-WT, or MG53-S255A and then subjected to hypoxia (n=6). **C**, Representative Western blots and averaged data showing phosphorylated and total Akt levels in NRVMs with adenovirus infection (n=6). **D**, Immunoprecipitation of Flag-tagged wild type or S255A mutant of MG53 with p85-PI3K-Myc (**upper**) or CaV3-Myc (**lower**) in HEK293 cells. **E** and **F**, NRVMs viability determined by LDH concentration in the medium (**E**) and Cell Counting Kit-8 (**F**) after incubated with HSA, rhMG53-WT, or rhMG53-S255A and challenged with hypoxia (n=8 for **E** and n=7 for **F**). **G** and **H**, Representative imaging and quantitative results of tracing FM1-43 dye entry showing the membrane repair function of rhMG53-WT and rhMG53-S255A in C2C12 myoblast cells treated with saponin (n=10 for HSA, n=6 for rhMG53-WT, and n=7 for rhMG53-S255A). Scale bars, 20 μm. Dye influx was quantified as the changes in fluorescent signal intensity over baseline (ΔF/F_0_). Adv-β-gal, Adv-MG53-WT, and Adv-MG53-S255A were cells infected with adenovirus expressing β-gal, MG53-WT, and MG53-S255A, respectively. Normal distribution was confirmed by Shapiro-Wilk test. Statistical analyses were performed with 2-way ANOVA with Sidak multiple comparisons test (**A**, **B**, and **E**) and 1-way ANOVA with Tukey post hoc test (**C** and **F**). Data are presented as mean±SEM. HSA, human serum albumin; MG53, mitsugumin 53; rhMG53-S255A, recombinant human MG53-S255A; and rhMG53-WT, recombinant wild type MG53.

Up to this point, we have shown that phosphorylation of S255 is essential for the E3 ligase activity of MG53, which may contribute to the detrimental effect of MG53 in impairing insulin sensitivity and glucose metabolism. We next examined whether phosphorylation of S255 is also required for the prosurvival function of MG53. In cultured neonatal rat ventricular myocytes, hypoxia induced dramatic cell damage as evidenced by increased LDH release (Figure 4A) and decreased cell viability (Figure 4B), which were fully blocked by overexpressing MG53-S255A, S255D, or S255E mutant, as was the case of overexpressing MG53-WT (Figure 4A and 4B; Figure S6). Furthermore, the S255A mutant increased Akt activation to the same extent as MG53-WT (Figure 4C), and it was able to form complex with p85 subunit of PI3K and CaV3 (caveolin-3; Figure 4D), which constitute important components of the reperfusion injury salvage kinase signaling complex.^19^ Thus, MG53-S255A is E3 ligase-deficient, but equally potent in protecting cardiac myocytes against harmful insults.

Additionally, previous studies have shown that recombinant MG53 protein exerts cell protective function extracellularly.^23^ To determine whether extracellular MG53-S255A can be used for therapeutic purposes, we incubated hypoxia-challenged neonatal rat ventricular myocytes with purified recombinant wild type or S255A mutant MG53 (rhMG53-WT or rhMG53-S255A, respectively). The rhMG53-S255A significantly reduced the LDH release and cell death incurred by hypoxia with indistinguishable potency relative to rhMG53-WT (Figure 4E and 4F). Moreover, saponin-induced perforation of the C2C12 cell membrane was profoundly alleviated by incubation with rhMG53-S255A as well as rhMG53-WT, as evidenced by markedly reduced FM1-43 fluorescence dye accumulation in C2C12 cells (Figure 4G and 4H, and Video S1–S3), corroborating that rhMG53-S255A is equally effective in facilitating membrane repair as rhMG53-WT. Thus, extracellular MG53-S255A can also protect the cells against various injuries. Taken together, although S255A mutation abolishes the E3 ligase activity of MG53, it does not disturb the beneficial role of MG53 in cell protection.

### Extracellular MG53-WT but not MG53-S255A Impairs Systemic Insulin Signaling

**Figure 5.**
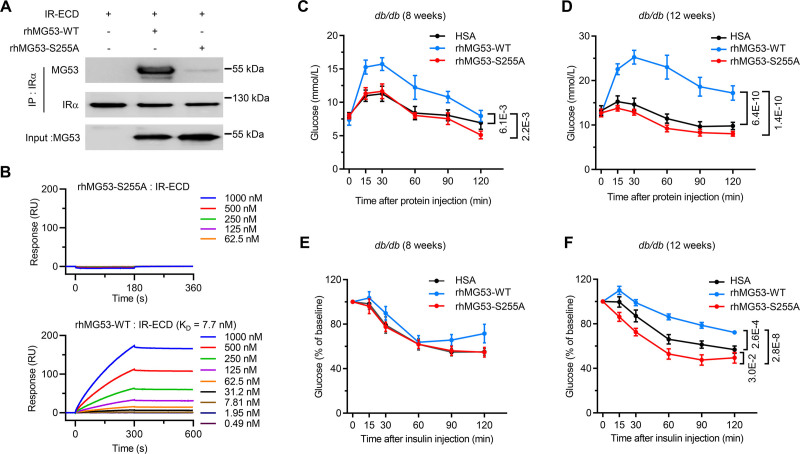
**rhMG53-WT, but not its E3 ligase-deficient mutant rhMG53-S255A, exaggerates hyperglycemia and insulin resistance in *db/db* diabetic mice. A**, Immunoprecipitation of purified recombinant extracellular domain of human insulin receptor (IR-ECD) with rhMG53-WT or rhMG53-S255A (n=3). **B**, Surface plasmon resonance (SPR) measurements illustrating MG53-S255A mutant abrogated binding of MG53 with IR-ECD. SPR experiment was performed by immobilizing IR-ECD on the chip, and injecting rhMG53-WT or rhMG53-S255A at indicated concentrations. **C** and **D**, Blood glucose levels at indicated time points of 8-week (**C**) and 12-week-old (**D**) *db/db* mice after i.p. injection with HSA, rhMG53-WT, or rhMG53-S255A recombinant protein (n=6). **E** and **F**, Results of insulin tolerance test of 8-week-old (n=10) (**E**) and 12-week-old (**F**) *db/db* mice (n=13 in HSA and rhMG53-WT groups, n=9 in rhMG53-S255A group) after i.p. injection with HSA, rhMG53-WT, or rhMG53-S255A. Normal distribution was confirmed by Shapiro-Wilk test. Data were analyzed using the 2-way ANOVA with Tukey post hoc test. Data are presented as mean±SEM. HSA, human serum albumin; MG53, mitsugumin 53; rhMG53-S255A, recombinant human MG53-S255A; and rhMG53-WT, recombinant wild type MG53; and STZ, streptozotocin.

Our previous studies have shown that MG53 is secreted under metabolic stress conditions such as high glucose or high insulin, and that circulating MG53 level increases with the development of metabolic diseases, contributing to the pathogenesis of systemic insulin resistance.^22^ Mechanistically, MG53 binds to IR-ECD and allosterically inhibits the receptor signaling.^22^ When MG53-S255A, MG53-S255D, or MG53-S255E was overexpressed in HEK293 cells, the mutant proteins could be detected in the culture media and their concentrations were comparable to that of MG53-WT (Figure S7A and S7B), suggesting that MG53-S255A/D/E can be secreted normally. However, unlike MG53-WT, MG53-S255A mutant could not bind to IR-ECD, as determined by co-IP and surface plasmon resonance assay (Figure 5A and 5B), implying that extracellular MG53-S255A does not interfere with insulin signaling. Indeed, in the diabetic *db/db* mice, blood glucose level was markedly increased and remained high for at least 2 hours after systemic delivery of rhMG53-WT (1 mg/kg body weight via intravenously injection), whereas it was unaltered in the diabetic mice in response to injection of rhMG53-S255A compared to the control group treated by HSA (human serum albumin; Figure 5C and 5D). Notably, MG53-exaggrated hyperglycemia and insulin insensitivity were much more severe in *db/db* mice at 12 weeks of age relative to the younger group (8-week-old) (Figure 5C–5F). In the 8-week-old *db/db* mice (Figure 5E) and the 12-week-old *db/+* control mice (Figure S7C), rhMG53-WT had no influence on their insulin sensitivity, highlighting that the detrimental effect of rhMG53-WT is dependent on the progression of diabetes (Figure S8). Importantly, rhMG53-S255A did not cause elevation in blood glucose levels no matter the mice were at 8 or 12 weeks of age (Figure 5C and 5D). In fact, rhMG53-S255A even moderately but significantly improved insulin sensitivity in 12-week-old *db/db* mice (Figure 5F). These results suggest that rhMG53-S255A is a safer drug candidate with potential beneficial effect on insulin sensitivity than rhMG53-WT in treating patients with various diabetic complications.

### rhMG53-S255A Protects the Diabetic Heart Against Acute I/R Injury

To investigate whether rhMG53-S255A can be used to protect the heart against I/R injury, we first compared the pharmacokinetics of rhMG53-S255A (1 mg/kg body weight, intravenous injection) with that of rhMG53-WT by an ELISA kit developed by our laboratory.^24^ The rhMG53-WT and rhMG53-S255A could be detected by this ELISA kit with comparable efficiency (Figure S9A), and the 2 proteins demonstrated similar turnover rates in vivo (Figure S9B). Then, we tested the efficacy of rhMG53-S255A in treating SD rats subjected to I/R procedure (Figure S10A). Delivery of rhMG53-WT or rhMG53-S255A by intravenous injection markedly attenuated I/R-induced myocardial infarction and cardiomyocyte death in SD rats (Figure S10B and S10C). Notably, the effectiveness of rhMG53-S255A was comparable to that of rhMG53-WT.

**Figure 6.**
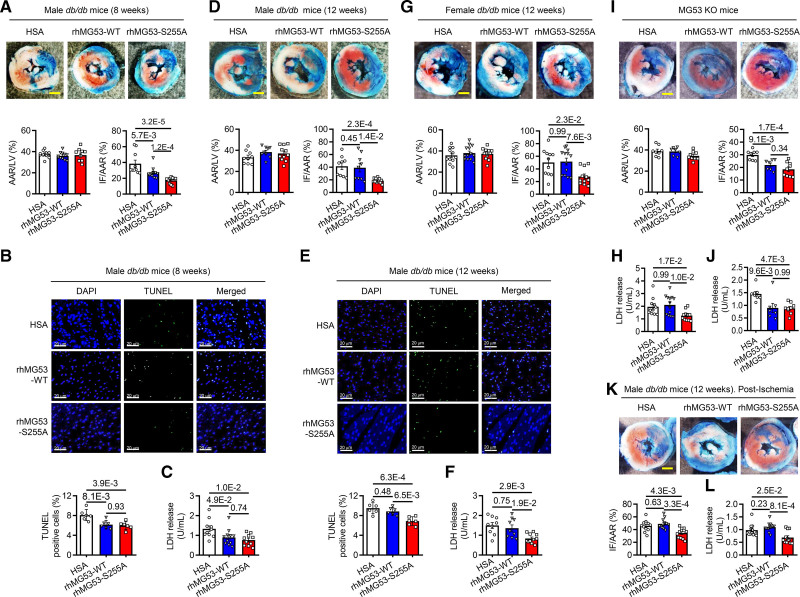
**rhMG53-S255A (recombinant human MG53-S255A) ameliorates ischemia/reperfusion (I/R)-induced cardiac injury in mice with early and advanced diabetes, whereas rhMG53-WT (recombinant wild type MG53) works only in the early phase. A**, **D**, **G**, and **I**, Representative images and quantitative data of infarct size (IF) and area-at-risk (AAR) in the heart of 8-week-old male *db/db* mice (n=10 in human serum albumin [HSA] or rhMG53-S255A group, n=11 in rhMG53-WT group) (**A**), 12-week-old male *db/db* mice (n=9 in HSA or rhMG53-WT group, n=11 in rhMG53-S255A group) (**D**), 12-week-old female *db/db* mice (n=11 in HSA or rhMG53-S255A group, n=13 in rhMG53-WT group) (**G**), and male MG53 knockout mice (n=8 in HSA, n=7 in rhMG53-WT, and n=9 in rhMG53-S255A group) (**I**) subjected to I/R injury (30 min ischemia/24-h reperfusion) treated with HSA, rhMG53-WT or rhMG53-S255A, as indicated. Scale bar, 1 mm. **K**, Representative images and quantitative data of IF and AAR in the heart of 12-week-old male *db/db* mice subjected to I/R injury with postischemia treatment (n=12). Scale bar, 1 mm. **B** and **E**, Representative images and quantitative data of TUNEL staining of myocardial sections in the hearts from 8-week-old (**B**) and 12-week-old (**E**) male *db/db* mice (n=6). Scale bar, 20 μm. **C**, **F**, **H**, **J**, and **L**, Serum LDH levels from 8-week-old male *db/db* mice (**C**), 12-week-old male *db/db* mice (**F**), 12-week-old female *db/db* mice (**H**), MG53-deficient male mice (**J**), and 8-week-old male *db/db* mice with Postischemia treatment (**L**). Number of samples measured in each group was the same as listed above. Normal distribution was confirmed by Shapiro-Wilk test. Data were analyzed using the Mann-Whitney *U* test (**A** and **L**), 1-way ANOVA with Tukey post hoc test (**B–F** and **I–K**) and Kruskal-Wallis test (**G** and **H**). Data are presented as mean±SEM. GSK3β indicates glycogen synthase kinase 3 beta; and MG53, mitsugumin 53.

To date, >60% deaths of T2D patients are caused by cardiovascular complications, and patients with diabetes experience poorer prognosis than those with cardiovascular diseases in the absence of diabetes.^2,3,25–28^ Since systemic delivery of rhMG53-WT, but not rhMG53-S255A, exacerbated hyperglycemia and insulin resistance in diabetic animals (Figure 5C–5F), we speculated that rhMG53-S255A is superior to rhMG53-WT in treating cardiac conditions with comorbidity of diabetes. Indeed, in 8-week-old male diabetic *db/db* mice challenged with I/R (Figure S10A), the protective effect of rhMG53-S255A was significantly stronger than that of rhMG53-WT, as manifested by greater reductions in the infarct size (Figure 6A), although both rhMG53-WT and rhMG53-S255A were able to ameliorate cardiac I/R damage (Figure 6A–6C). Most importantly, as the diabetic conditions of *db/db* mice progressed till they were at 12 weeks of age, treatment with rhMG53-WT failed to alleviate cardiac injury induced by I/R, whereas rhMG53-S255A could still profoundly decrease the infarct area by half and reduced myocardial cell death (Figure 6D–6F). Similar results were observed in 12-week-old female diabetic *db/db* mice subjected to I/R injury (Figure 6G and 6H). Since the expression level of MG53 is very low in the human heart,^29,30^ we further examined the potential effects of rhMG53-WT or rhMG53-S255A in the MG53 knockout mice insulted by I/R. We found that both recombinant proteins ameliorated the damage in the heart lacking endogenous MG53 (Figure 6I and 6J). To better mimic the clinical situation, we performed postischemia protocol that 1 single injection of protein was given 5 minutes after reperfusion (Figure S11A). Using this treatment regimen, rhMG53-S255A also effectively reduced the infarct sizes and cardiac cell death, while rhMG53-WT had no significant effect in the 12-week-old *db/db* mice (Figure 6K and 6L). These results indicate that rhMG53-S255A not only retains the cardiac protective function of MG53, but also displays strong therapeutic effect in the condition of advanced diabetes where rhMG53-WT fails to be functional.

### rhMG53-S255A Protects the Diabetic Heart Against I/R-Induced Heart Failure and Maladaptive Remodeling

**Figure 7.**
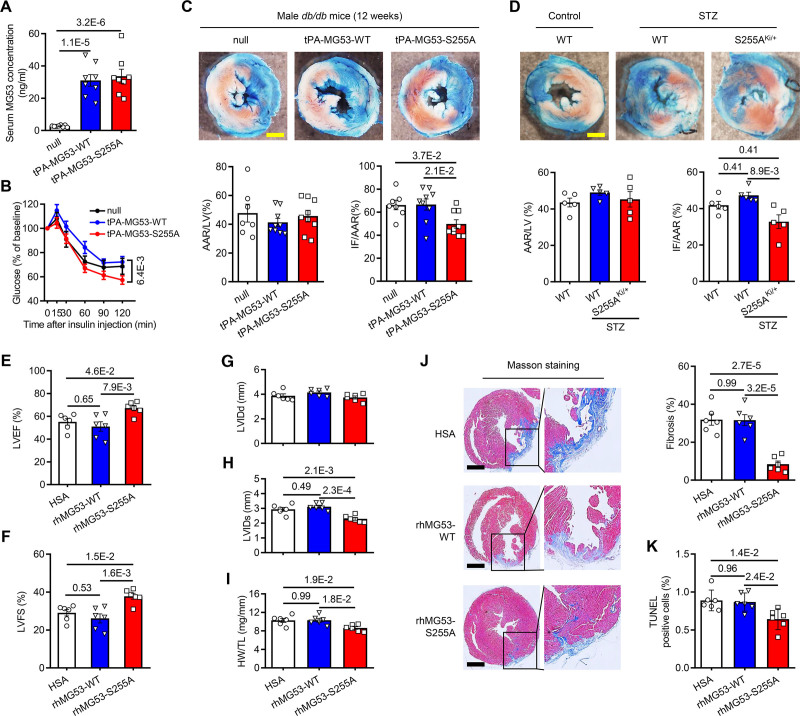
**Chronic treatment of rhMG53-S255A ameliorates I/R-induced myocardial injury, maladaptive remodeling and cardiac dysfunction in diabetes mice. A**, Serum MG53 levels in 12-week-old male *db/db* mice injected with empty adenoviral vector (null) or adenoviral vector expressing tPA-MG53-WT or tPA-MG53-S255A (n=8 for each group). **B**, Results of insulin tolerance test (ITT) of *db/db* mice 7 days after injection with adenoviral expression vectors via a tail vein (n=9 in null, n=15 for tPA-MG53-WT and tPA-MG53-S255A groups). **C**, Representative images and quantitative data of infarct size (IF) and area-at-risk (AAR) in the hearts of the infected *db/db* mice subjected to I/R injury (30 min ischemia/24-h reperfusion) (n=7 in null, n=9 for tPA-MG53-WT and tPA-MG53-S255A groups). **D**, Representative images and quantitative data of IF and AAR in the hearts of streptozotocin (STZ)-induced diabetic S255A^Ki/+^ or littermate WT mice (n=5). **E–I**, Averaged data of echocardiogram, including left ventricular ejection fraction (LVEF) (**E**), fractional shortening (LVFS) (**F**), diastolic left ventricular internal diameter (LVIDd) (**G**), systolic left ventricular internal diameter (LVIDs) (**H**), and the ratio of heart weight (HW) to tibia length (TL) (**I**) of the mice 4 weeks after being subjected to I/R injury (reperfusion after 30-min ischemia) and treated with HSA, rhMG53-WT, or rhMG53-S255A (n=6). **J** and **K**, Representative images and quantitative data of Masson’s trichrome staining (**J**) and TUNEL staining (**K**) of sections of the heart 4 weeks after I/R injury and with HSA, rhMG53-WT or rhMG53-S255A treatment (n=6 for each group). Scale bar, 1 mm. Normal distribution was confirmed by Shapiro-Wilk test. Data were analyzed using 1-way ANOVA (**A**, **C**, and **E–K**), 2-way ANOVA (**B**) with Tukey post hoc test, and the Kruskal-Wallis test (**D**). Data are presented as mean±SEM. HSA, human serum albumin; IR-ECD, extracellular domain of IR; MG53, mitsugumin 53; rhMG53-S255A, recombinant human MG53-S255A; rhMG53-WT, recombinant wild type MG53; and S255, serine 255.

To evaluate the effects of secreted wild type and the mutant MG53, we overexpressed human wild type or S255A mutant MG53 fused with a signal peptide of tissue plasminogen activator^24,31,32^ using adenoviral gene transfer, which increased serum wild type and S255A mutant to a similar level in the *db/db* mice (Figure 7A). The mice with increased circulating tPA-MG53-S255A displayed improved insulin sensitivity relative to those with increased tPA-MG53-WT (Figure 7B). When subjected to I/R injury, the tPA-MG53-S255A group had significantly reduced infarct size (Figure 7C). Moreover, we established a streptozotocin-induced diabetic model to evaluate the cardiac protective effect of S255A in knock-in mice (Figure S12A and S12B). Remarkably, the heterozygotes of MG53-S255A knock-in (S255A^Ki/+^) mice displayed significantly reduced infarct size relative to their wild type littermates after I/R challenge (Figure 7D), while the MG53 protein levels in serum and skeletal muscle of S255A^Ki/+^ were comparable to the normal endogenous levels (Figure S12C and S12D). Taken together, these results substantiate our findings that, unlike MG53-WT, the circulating S255A mutant is devoid of detrimental effects on metabolism, and thus, may better protect the diabetic heart against a variety of injuries.

To test the long-term effects of treatment with rhMG53-S255A, 8-week-old diabetic *db/db* mice were subjected to I/R injury and treated with rhMG53-WT, rhMG53-S255A, or HSA (Figure S11B). Four weeks after I/R and protein treatment, echocardiography was performed to evaluate the cardiac function and dimension of these mice. In the rhMG53-S255A-treated group, both ejection fraction and fractional shortening were significantly improved compared with those of the rhMG53-WT- or HSA-treated groups (Figure 7E and 7F). Although the left ventricular internal diameter end diastole was not different among the 3 groups, the ventricular internal diameter end systole of the mice treated with rhMG53-S255A was smaller than those of the other 2 groups (Figure 7G and 7H). Consistent with the echocardiography results, cardiac hypertrophy indexed by the ratio of heart weight/tibia length was significantly reduced after rhMG53-S255A treatment (Figure 7I). Moreover, myocardial fibrosis and TdT-mediated dUTP Nick-end labeling positive cardiomyocytes were markedly reduced in response to the treatment of rhMG53-S255A but not rhMG53-WT (Figure 7J and 7K; Figure S13). These results indicate that rhMG53-S255A not only effectively attenuates I/R-induced acute myocardial injury but also protects the heart against chronic cardiac remodeling such as myocardial hypertrophy and fibrosis.

**Figure 8.**
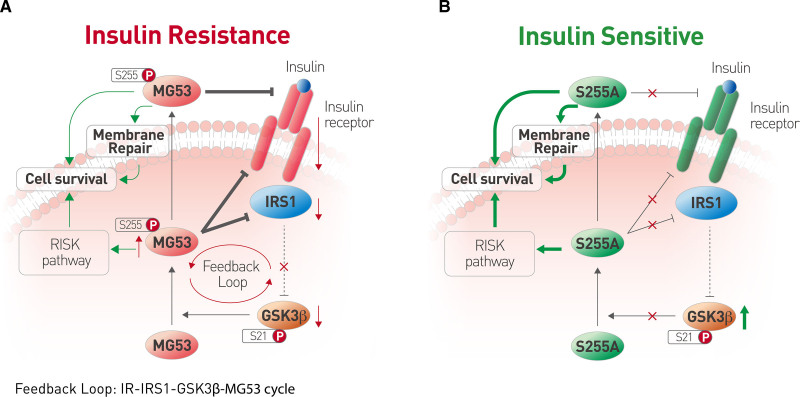
**Schematic illustration of the difference between wild-type MG53 and its E3 ligase-deficient mutant S255A. A**, Under the pathological conditions of insulin resistance, insulin-stimulated inactivation of GSK3β is impaired, which markedly enhanced the phosphorylation of MG53 at S255 and facilitates the downregulation of IR and IRS1; which in turn, exaggerates insulin resistance. **B**, MG53-S255A mutant lacks both intracellular E3 ligase activity and extracellular allosteric IR blocker function, breaking the IR-IRS1-GSK3β-MG53 vicious cycle, thus improving insulin sensitivity. Meanwhile, MG53-S255A still maintains the prosurvival and protective functions of MG53. GSK3β indicates glycogen synthase kinase 3 beta; IR, insulin receptor; IRS1, insulin receptor substrate 1; MG53, mitsugumin 53; and S255, serine 255.

Taken together, we have shown that the phosphorylation of MG53 at S255 by GSK3β activates the E3 ligase activity that is responsible for its adverse effect on metabolism (Figure 8A). Mutation of S255 abolishes the undesired detrimental effects, while preserves the prosurvival function of MG53 (Figure 8B). rhMG53-S255A rather than rhMG53-WT displays therapeutic effects in the diabetic heart experiencing I/R injury. Thus, rhMG53-S255A is a promising novel therapy for the treatment of T2D-associated acute myocardial damages as well as chronic cardiac maladaptive remodel and cardiac dysfunction.

## Discussion

Over the past decade, a large body of evidence has shown that MG53 is a double-edged sword, involved in regulating both cell protection and energy metabolism.^33^ For cell protection, MG53 is an integral component of membrane repair machinery^17^ and involved in the activation of reperfusion injury salvage kinase signaling pathway in the heart in response to various insulting stimuli.^18,19^ However, MG53 also functions as an E3 ligase that catalyzes the ubiquitination of its substrates, such as IR, IRS1, and AMPKα,^20,21,34,35^ thereby leading to metabolic disorders when upregulated. Thus, to explore its therapeutic potential in cardiac protection, it is necessary to get rid of its detrimental effects on metabolic homeostasis. In this study, we have 3 major findings. First, by analyzing the posttranslational modifications of MG53, we have identified S255 as an essential amino acid residue for MG53 to function as an E3 ligase. Second, we have found that S255 can be phosphorylated by GSK3β, and that this modification is required for the interaction of MG53 with its substrates, including IR and IRS1. Importantly, the mutant MG53-S255A is E3 ligase-deficient, but fully intact in terms of the cardiac protective function of MG53. These findings indicate that the application of recombinant rhMG53-S255A holds a great promise for the treatment of diabetes-associated acute myocardial infarction and I/R injury as well as chronic cardiac remodeling and dysfunction, in particular, in individuals with full-blown diabetes.

### Posttranslation Modification Regulates Function of MG53

Previous studies have shown posttranslational modifications of MG53 influence its biological or pathologic functions. For instance, relative to MG53-WT, modifying MG53 with S-nitrosylation at cysteine 144 or MG53 C144S mutant can more effectively protect the heart from I/R injury both ex vivo and in vivo, while the E3 ligase activity of MG53 is unaffected.^36^ Another cysteine residue C242 has been implicated in sensing the redox status of the environment and facilitating the oligomerization of MG53 in membrane repair.^17^ In addition, arginines 207 and 260 can be mono-ADP-ribosylated and recruited to the sites of membrane damage.^37^ It is noteworthy that all of these known modifications are related to cell protective function of MG53. Here we have shown, for the first time, that phosphorylation of S255 by GSK3β is important for MG53 to function as an E3 ligase. Mechanistically, we have demonstrated that S255 phosphorylation is essential not only for intracellular MG53 to bind to its E3 ligase substrates, but also for extracellular MG53 to bind to IR-ECD and work as an allosteric blocker of IR. Moreover, under disease conditions such as obesity and diabetes, GSK3β-mediated MG53 phosphorylation is robustly enhanced, resulting in markedly diminished IR and IRS1 levels in the heart and skeletal muscle and exaggerated systemic insulin resistance (see below for further discussion).

### GSK3β Negatively Regulates Insulin Signaling via Phosphorylating MG53 at S255

It has been known that the expression of GSK3β is increased in the skeletal muscle of T2D patients^14^ and animal models,^38^ and that muscle-specific knockout of GSK3β improves glucose handling and insulin sensitivity.^12^ Diabetes-associated enhancement of GSK3β activity increases phosphorylation of IRS1 at S332/336, which functionally inhibits IRS1 via attenuating its tyrosine phosphorylation by IR.^6,7^ Notably, several studies reported increased GSK3β activity and decreased protein abundance of IRS1 and IR in diabetes,^14,38–43^ although the connection between these 2 observations has not been established. Here, we have demonstrated that the reduced IR and IRS1 protein levels are attributable to not only the increased expression of MG53 but also the elevated phosphorylation at S255 by GSK3β in the context of diabetes (Figure 8A). The downregulation of IR and IRS1 leads to impaired Akt activation by insulin, which then cannot effectively deactivate GSK3β. Subsequently, hyperactive GSK3β induces phosphorylation of IRS1 at S332/336, resulting in attenuated IRS1 activation by IR-mediated tyrosine phosphorylation. Meanwhile, enhanced phosphorylation of MG53 at S255 by GSK3β enables its E3 ligase activity to mediate the degradation of both IR and IRS1. Thus, GSK3β-MG53-IR/IRS1 form a vicious cycle that exacerbates insulin resistance and glucose intolerance (Figure 8A).

### rhMG53-S255A is Beneficial, Whereas rhMG53-WT is Toxic in Mice With Advanced Diabetes

We have provided multiple lines of evidence to demonstrate that it is feasible to dissect different functional roles of MG53, and separate the beneficial effects from detrimental ones. Specifically, the mutation at S255 eliminates the E3 ligase activity and the related detrimental effects of MG53 on insulin signaling and metabolic homeostasis. On the contrary, S255A mutation does not affect the function of MG53 in activating reperfusion injury salvage kinase pathway or facilitating membrane repair, as evidenced by its preserved ability in promoting signaling complex formation (Figure 4D) and maintaining membrane integrity (Figure 4G and 4H), which may account for the robust cardiac protective function of S255A mutant (Figures 6 and 7, and Figure S10B and S10C).

Remarkably, in the diabetic *db/db* mice that suffered hyperglycemia and insulin resistance, rhMG53-S255A outperformed rhMG53-WT in cardiac protection against I/R injury, although rhMG53-S255A and rhMG53-WT displayed similar efficacy in protecting cardiac myocytes challenged with hypoxia in vitro and in normal mice or rats subjected to I/R in vivo. More importantly, MG53-S255A displayed superiority over WT in both the *db/db* mice with advanced diabetes and streptozotocin-induced diabetic S255A^ki/+^ mice. These are, at least in part, attributed to the fact that increasing circulating MG53 causes whole body insulin resistance.^22^ Injection of rhMG53-WT induced significant elevation in blood glucose on top of hyperglycemia in *db/db* mice, which was more severe and harmful in mice with advanced diabetes whose insulin sensitivity and glucose handling ability were deteriorated. In the 12-week-old mice with advanced diabetes, the protective effects of rhMG53-WT was largely offset by its damaging function in raising blood glucose level, in line with previous notion that the fluctuation in glucose level is a major risk factor for hospitalized patients and negatively associated with the prognosis after surgery.^44^ In fact, the association between high glucose and increased mortality after myocardial infarction is observed in patients with and without diabetes.^45^ It has also been reported that hyperglycemia is associated with impaired microvascular function and may be responsible for the no-reflow phenomenon in the culprit vessel after acute myocardial infarction, which contributes to enlarged infarct size and aggravated cardiac dysfunction.^46^ Therefore, the overall therapeutic outcome cannot justify the risk of utilizing rhMG53-WT in treating patients with myocardial infarction or I/R, especially those with insulin resistance and diabetes. In contrast, rhMG53-S255A does not bind to the IR-ECD or influences glucose handling, thus inflicting no challenge on blood glucose control. If any effect on insulin signaling, the mutant actually improves insulin sensitivity in mice with advanced diabetes (Figure 5F). This characteristic of rhMG53-S255A is especially advantageous for individuals with diabetes. This is because that, first, due to their compromised glucose handling capability, T2D patients are more vulnerable to fluctuations in blood glucose. Second, patients with T2D are predisposed to cardiac incidences, and the trend is increasing particularly in patients with heart failure with preserved ejection fraction.^47^ Of note, the therapeutic strategy for heart failure with preserved ejection faction is still an unmet medical need, and there are very limited options.

In summary, we have demonstrated that the phosphorylation of MG53 at S255 by GSK3β is necessary for its function as an E3 ligase, and that the phosphorylation is markedly enhanced in the heart as well as skeletal muscle of animals with metabolic disorders, in particular, diabetes. Importantly, MG53-S255A mutant can get rid of the E3 ligase property and resultant side effects on metabolic homeostasis, but does not disturb the prosurvival and protective functions of MG53. Therefore, the application of rhMG53-S255A in treating acute myocardial infarction and I/R injury as well as various insulting factors-induced chronic cardiac remodeling and dysfunction holds a great therapeutic potential, especially in diabetic settings.

## Article Information

### Acknowledgments

We thank the Analytical Instrumentation Center at Peking University for the help by Dr Wen Zhou with Mass spectrometry experiment and data analysis.

### Sources of Funding

This work was supported by the National Key R&D Program of China (2018YFA0507603, 2018YFA0800701, and 2018YFA0800501), the National Natural Science Foundation of China (31970722, 81630008, 81790621, and 31521062), and the Beijing Natural Science Foundation (5202010).

### Disclosures

None.

### Supplemental Material

Expanded Materials and Methods

Figures S1–S13

Video S1–S3

References 48–56

## Supplementary Material
