# Supplementary material for: Blocking MG53S255 Phosphorylation Protects Diabetic Heart From Ischemic Injury
Source: Circ Res. 2022 Nov 7;131(12):962–76. doi: 10.1161/CIRCRESAHA.122.321055 (PMC9770150; doi:10.1161/CIRCRESAHA.122.321055)
Supplement: Supplementary file 4 [file res-131-0962-s004.pdf]

SUPPLEMENTAL MATERIAL

Animals

Animals were maintained in the Laboratory Animal Center at Peking University, Beijing, China. This facility is accredited by AAALAC. The animals were randomly allocated to experimental groups. All procedures were performed following protocols approved by the IACUC of Peking University, and conformed to the Guide for the Care and Use of Laboratory Animals (NIH publication No. 86-23, revised 2011).

The generation of *mg53*$^{-/-}$ mice was described previously[20,48,49]. Diabetic *db/db* mice (#T002407) and age- and gender-matched lean control *db/+* mice were purchased from GemPharmatech Co., Ltd. (Nanjing, China). The S255A knock-in mouse line was generated by Cyagen Biosciences Inc (Guangzhou, China). Male Sprague-Dawley (SD) rats (#101) at 8 weeks of age and age-matched control rats were purchased from Vital River Laboratories (Beijing, China). For dietary intervention, male C57/BL6N mice were fed a high-fat diet (HFD, Research Diets Inc., #D12492) or standard chow (Academy of Military Medical Sciences, Beijing, China) for 20 weeks starting from 3 weeks of age. To induce diabetes, 8-week-old male S255A$^{Ki/+}$ and their age- and gender-matched wild type littermates were fed HFD for 1 week, then injected with streptozotocin (STZ, 100 mg/kg) and continued on HFD for another 3 weeks. The development and characterization of a nonhuman primate (NHP) model of spontaneous insulin resistance and metabolic syndrome were reported previously[50]. No non-inclusion or exclusion parameters were used in our studies.

Metabolic parameters

To test the influence of administrating recombinant protein on blood glucose level, mice were fasted overnight (for 16 hours) and injected *i.p.* with HSA, rhMG53-WT, or rhMG53-S255A (1 mg/kg body weight). For ITTs, mice were fed *ad libitum* and challenged with bovine insulin (*i.p.* at 0.75 U/kg, Sigma-Aldrich, #I0516) mixed with HSA, rhMG53-WT or rhMG53-S255A (1 mg/kg body weight). For both tests, blood samples were collected from a tail vein right before the injection of recombinant proteins or insulin which was considered as time 0, and at indicated time points after injection. Blood glucose was measured with an AccuCheck blood glucose meter (Roche Diagnostics Inc., ACCU-CHECK Performa).

Cell culture, plasmid transfection, and adenoviral infection

HEK293 cells were from ATCC (#CRL-1573) and authenticated by Short Tandem Repeat (STR) analysis. The cells were maintained in Dulbecco's modified Eagle's medium (DMEM) supplemented with 10% fetal bovine serum (FBS, Sigma-Aldrich, #F6178). For plasmid transfection, Lipofectamine 2000 (Invitrogen, #11668027) was used according to the instruction by manufacturer. 48 hours after transfection, the culture medium was collected and centrifuged at 4,000 rpm in Amicon Ultra-4 10K Centrifugal Filter Devices (Millipore, #UFC801096) for 20 min to concentrate MG53 for detection by Western blotting.

The isolation of neonatal rat ventricular cardiomyocytes (NRVMs) and adenovirus infection to overexpress MG53 in NRVMs were described previously[51].

Hypoxia treatment

Hypoxia treatment was performed as previously described[19]. In brief, cells were cultured in DMEM for 48 hours after adenoviral infection. Then, the medium was changed to serum-free RPMI1640 saturated with 95% N_2 /5% CO_2, and cells were placed in a 37 °C airtight box saturated with 95% N_2/5% CO_2 for 12 hours. O_2 concentrations were < 0.1 % (Billups-Rothenberg Inc., modular incubator chamber, #92014). For normoxia controls, cells were placed in a 37°C / 5% CO_2 incubator for 12 hours before analysis. Cardiomyocyte viability was assessed by an ATP assay as previously described[19]. The LDH concentration in the culture medium was spectrophotometrically assayed using a kit from Shanghai Gensource Co., Ltd. (#LDH0360).

To measure the protective effect of rhMG53-WT and rhMG53-S255A, NRVMs were cultured in serum-free RPMI 1640 with 100 μg/mL HSA, rhMG53-WT or rhMG53-S255A saturated with 95% N_2/5% CO_2 for 16 hours. O_2 concentrations were < 0.1 % (Billups-Rothenberg Inc., modular incubator chamber, #92014). For normoxia controls, cells were placed in an incubator with 5% CO_2 at 37°C for 16 hours before analysis. The Cell Counting Kit-8 (CCK-8) was used to measure cell viability. For the CCK-8 assay, NRVMs were seeded into 96-well plates at 5×10^3 cells per well. After hypoxia, the cytotoxicity was assessed by incubating the cells with CCK-8 reagent (Solarbio, #CA1210) for 2-4 hours, and measuring the absorbance at 450 nm with a microplate reader (BioTek).

Plasmids and adenoviral vectors

DNA fragments corresponding to full-length or ΔRING MG53 was amplified from a mouse cDNA library by PCR and inserted into pcDNA4/TO/Myc-His B expression vector (Invitrogen, #V103020) between the KpnI and XhoI restriction sites. The Flag-tagged MG53 has been described previously[20]. MG53 mutants, including S2/13A, S189A, S255A, and S307A with the

corresponding serine substituted by alanine, were generated from the wild type MG53 construct (MG53-WT) by point mutation using Stratagene's QuikChange II site-directed mutagenesis kit (Stratagene, #210518). IRS1 was sub-cloned from pBS mouse IRS1 (Addgene, #11026) and inserted into pcDNA4/TO/myc-His B expression vector (Invitrogen, #V103020) between the HindIII and NotI restriction sites. The coding sequence of p85 was amplified from a mouse fetal liver cDNA library and inserted into pcDNA4/TO/myc-His B expression vector between the BamHI and XbaI restriction sites. The coding sequence of CaV3 was amplified from a mouse fetal liver cDNA library and inserted into pcDNA3.1(+)-myc-HisA expression vector (Invitrogen, #V80020) between the EcoRI and XbaI restriction sites. The HA-tagged GSK3β has been described previously[52].

Adenovirus expressing MG53-WT, MG53-S255A, MG53-ΔRING and β-galactosidase (Adv-β-gal) were generated by BAC Biological Technology (Beijing, China).

Adenoviral vector expressing tPA-MG53-WT or tPA-MG53-S255A was constructed by fusing a tissue polypeptide antigen (tPA) signal peptide at the N-terminus of human wild type or S255A mutant MG53. The coding sequence of tPA signal peptide is: 5'-ATGGATGCAATGAAGAGAGGGCTCTGCTGTGTGCTGCTGCTGTGTGGAGCAGTCTTC GTTTCGCCC-3'[53]. For adenovirus-mediated gene delivery, 100 μL high titer adenovirus (> 1×10^8 cfu/mL) was diluted in saline and then injected intravenously as 5×10^5 cfu/kg body weight into mice[53].

Chemicals and antibodies

Unless indicated otherwise, all chemicals were from Sigma-Aldrich. Human insulin receptor extracellular domain protein (#INR-H5220) was from ACROBiosystems.

For Western blotting, the anti-phospho-Akt antibody (phosphorylation at Ser473; #4060; 1:1,000), anti-total-Akt antibody (#9272; 1:1,000), insulin receptor β subunit antibody (#3025; 1:1,000), insulin receptor substrate 1 antibody (#2382; 1:1,000), anti-total-GSK3β (#9315; 1:1,000) and β-Catenin antibody (#9562; 1:1,000) were from Cell Signaling Technology. GAPDH antibody (#BE0023; 1:10,000) was from Bioeasy Technology. Myc-tag antibody (#M5546; 1:5,000) and Flag-tag antibody (#F1804; 1:5,000) were from Sigma-Aldrich. Insulin receptor α subunit antibody (#ab36550; 1:1,000) was from Abcam. The anti-phospho-GSK3β antibody (phosphorylation at Ser9; #MA5-14873; 1:1,000) was from Invitrogen. The custom-made monoclonal antibody against MG53 was described previously[22]. Brilliant Green (#5141-20-8) for staining of the non-specific (NS) bands was from Amresco. LY2090314 (#HY-16294) and CHIR99021 (#HY-10182) were from MedChemExpress.

Monoclonal antibody specifically reacted with the phosphorylated MG53 at S255 was custom-manufactured by contract research organization (Chempartner, Inc., Shanghai, China). Briefly, phosphor-modified peptide CLQKILSE-pS-PPPARL was synthesized and was used to immunized Balb/c mice. Then the splenocyte was isolated and generate hybridomas. The optimal antibody from hybridomas was screened by ELISA with unmodified (CLQKILSESPPPARL) and phosphor-modified peptide and confirmed by Western blotting.

Western blotting assay

Tissue samples or cells were lysed in lysis buffer (30 mM HEPES at pH 7.6, 100 mM NaCl, 0.5% Nonidet P-40, and protease inhibitor cocktail) on ice for 10 min. The lysates were then centrifuged at 13,000 rpm for 10 min, and the supernatant was used for Western blotting as described previously[20]. All the representative images are representative of the average.

Co-immunoprecipitation and in vitro kinase assay

Cell or skeletal muscle lysates, or recombinant proteins were mixed with Protein A Sepharose™ 4 Fast Flow (GE Healthcare, #17-5280-02) and 0.5 μg antibody in 500 μL ice-cold PBS buffer, and incubated at 4 ºC for 3 hours. The resins were then washed 5 times with ice-cold PBS buffer. The immunoprecipitated proteins were resolved on SDS-PAGE and detected by corresponding antibodies by Western blotting.

For *in vitro* kinase assay, plasmids expressing GSK3β-HA or MG53-Myc were transfected into HEK293T cells, and proteins were partially purified from cell lysate by immunoprecipitation with anti-HA and anti-Myc antibodies, respectively. 1 μg of purified protein was used in the reaction mixture containing kinase buffer (20 mM Tris-HCl, pH 7.5, 5 mM $MgCl_2$, 0.5 mM DTT, and 150 mM KCl), with 5 μM ATP, in the presence or absence of 5 μCi of [γ-^{32}P] ATP. The reactions were performed at 30°C for 30 min and stopped by boiling in Laemmli buffer (BIO-RAD, #1610737). The phosphorylated proteins were analyzed in 10% SDS-PAGE. Gels were stained with Coomassie Blue or transferred to Immobilon-P membrane (Millipore, #IPVH00010), and the incorporated radioactivity was determined.

Purification of recombinant proteins

The human wild type MG53 (rhMG53-WT) and S255A mutation (rhMG53-S255A) were purchased from Origene Technology. CO., Ltd (Beijing, China). Both recombinant proteins were purified from bacterial host strain M15. Expression vectors for Myc-tagged MG53-WT, MG53-S255A, and IRS1 were transfected into HEK293 cells. The Myc-tagged proteins were purified by affinity purification using EZview Red Anti-Myc Affinity Gel (Sigma-Aldrich, #CFAD-E6654).

Mass spectrometry (MS)

For the analysis of MG53 phosphorylation and interacting proteins, the total proteins from C57BL/6N mouse skeletal muscle or HEK293 overexpressing human MG53 were extracted with lysis buffer A (30 mM HEPES at pH 7.6, 100 mM NaCl, 0.5% Nonidet P-40, and protease inhibitor cocktail). MG53 was partially purified with custom MG53 antibody[22], and then subjected to MS analysis as described previously[54]. Data processing was carried out using Thermo Proteome Discoverer 2.4 using the human or mouse database downloaded from uniprot.org.

Surface Plasmon Resonance (SPR) experiments

All SPR experiments were performed with a Biacore T200 instrument (Biacore, Uppsala, Sweden) as previously descripted[22]. Briefly, recombinant IR-ECD or IRS1 was immobilized on a CM5 sensor chip with free carboxyl groups on a dextran matrix by using Amine Coupling Kit (GE Healthcare) according to the manufacturer's instructions. The protein to be immobilized was dissolved in 10 mM sodium acetate buffer (pH 4.5) and then injected to couple to the active sites on the sensor surface via the primary amine groups, and the remaining active sites in the test flow cell were blocked by 1 M ethanolamine. A reference flow cell was activated and blocked in the

absence of protein. The immobilization level was fixed at 500 Biacore response units (RU), and gradient concentrations of analyte were serially injected to flow over the chip surface. All binding experiments were performed in PBS-P buffer (20 mM phosphate buffer, 2.7 mM NaCl, 137 mM KCl, 0.05% vol/vol surfactant P-20, pH 7.4) at 25 ºC. Regeneration was achieved by injection of 10 mM NaOH (pH 10) after each sample injection. The signal was monitored by subtracting reference flow cell from test flow cell. Data analysis was performed with Biacore T200 Evaluation Software. All K_D values were fit to a 1:1 bimolecular binding model and obtained from kinetic analysis.

Glucose uptake

Glucose uptake assay was performed as previously descripted[34]. In brief, cellular glucose uptake was measured by the reduction of glucose content in the culture medium. 2×10^6 C2C12 cells were plated in 35 mm dishes and differentiated into myotubes. C2C12 myotubes were treated with serum-free, high glucose DMEM medium overnight before switched to glucose-free DMEM medium (ThermoFisher, #11966) for 6 hours. Then the medium was changed to high glucose DMEM medium with or without 100 nM bovine insulin (Sigma-Aldrich, #I0516). The time right before medium-change was regarded as time 0, and the medium glucose concentration was recorded at 0 and 4 hours after incubation with an Accu-Check blood glucose meter (Roche Diagnostics Inc.).

Membrane repair assay

The membrane repair assay was designed according to previous studies[17,23]. Briefly, C2C12 cells were seeded in glass-bottom dishes (BioptechsNEST, #801001). 12 hours after plating, culture media was replaced with Tyrode solution (140 mM NaCl, 5 mM KCl, 2.5 mM $CaCl_2$, 2 mM $MgCl_2$ and 10 mM HEPES, pH 7.2) containing 80 μg/mL HSA, rhMG53-WT, or rhMG53-S255A. To induce damage to the cells, saponin (Sigma-Aldrich, #S4521) was added into Tyrode to a final concentration of 0.0017%. Cells were imaged on a PerkinElmer UltraVIEW VoX system with a 60 × 1.4-NA (numerical aperture) oil immersion objective (Nikon) in the presence of 2.5 μM FM1-43 dye (Invitrogen, #T3163). Images were captured at intervals of 1.5 s each for up to 150 s. The resulting data were analyzed by calculating the change in fluorescence intensity ($\Delta F/F_0$) between each captured frame with ImageJ software.

I/R injury and infarct size measurements

I/R surgery and area-at-risk and infarct size measurements were performed as previously described[19]. Briefly, animals were challenged with cardiac ischemia by tightening a snare occluder around the left anterior descending coronary artery for 30 min (45 min for rats), and were then allowed to recover from the surgery. After reperfusion for 24 hours, the animal was sacrificed. Blood samples were collected for LDH measurement using a kit from Sigma-Aldrich (#MAK066) as previously described[19]. The heart was excised and coronary artery was re-occluded at the site of occlusion. Then the ascending aorta was cannulated (distal to the sinus of Valsalva) and perfused retrogradely with 0.05% Alcian blue (Sigma-Aldrich, #A3157) to visualize the area-at-risk. The heart was briefly frozen at −80 °C for 10 min and cut into slices (5–6 slices/heart). The slices were then incubated for 15 min in sodium phosphate buffer containing 1% 2,3,5-triphenyl-tetrazolium chloride (Sigma-Aldrich, #T8877) to visualize the unstained infarcted region. The areas of infarct

and left ventricular were determined by planimetry with ImageJ (http://imagej.nih.gov/ij/). The infarct size was calculated as infarct area divided by area-at-risk (IF/AAR). All the representative images are representative of the average.

Histological Analysis

The tissues for histological analysis were fixed overnight in 4% paraformaldehyde (pH 7.4), embedded in paraffin, serially sectioned at 5 μm. Masson's trichrome staining was performed as previously described[53,55]. The CardioTACS in situ apoptosis detection kit (Roche, #11684795910) was used for TUNEL staining according to the manufacturer's instructions as previously described[56], which were observed under a fluorescence microscope (Leica, German/DM4B).

Echocardiography

Mice were subjected to echocardiographic analysis under 1% isoflurane using a VEVO-2100 digital imaging system (Visual Sonics). Left ventricular diameter end-diastole (LVIDd) and end-systole (LVIDs) were measured from two-dimensional guided short-axis M-Mode views of left ventricle. Left ventricular ejection fraction (LVEF) was calculated using the equation: $LVEF (\%) = ((LVIDd)^3 - (LVIDs)^3)/(LVIDd)^3 \times 100$, and left ventricular fraction shortening (LVFS) was calculated using $LVFS (\%) = (LVIDd - LVIDs)/LVIDd \times 100$. The data were averaged from 5 cardiac cycles. The data were analyzed blindly.

Statistical analysis

Statistical analyses were performed using GraphPad Prism version 8.01(GraphPad Software, Inc. RRID: SCR_002798, San Diego, CA, USA) and the SPSS 24.0 software package (IBM SPSS Statistics, version 24.0, Armonk, NY, USA). Data are presented as means ± SEM. All data sets were tested for normal distribution using Shapiro-Wilk test. The homogeneity of variances was examined using Levene's test. Differences between groups with normally distributed data were analyzed using a two-sided unpaired *t* test (for 2 groups of data), one-way ANOVA followed by Tukey post hoc test (for 3 or more groups of data), two-way ANOVA with Sidak's multiple comparisons test or repeated measures ANOVA (for repeated measures data). If the data did not have normal distribution or the number of cases was too small to determine normality ($n < 6$), Mann-Whitney *U* test with two-tailed *p* values (for 2 groups of data) or Kruskal-Wallis test followed by Mann-Whitney *U* test (for 3 or more groups of data) was used, and Bonferroni-adjusted *p* values were reported where multiple comparisons were tested. Only within-test corrections were made in this study.

Fig. S1: Identification of phosphorylation sites in MG53.

A, Spectral counts of peptides containing indicated phosphorylated amino acid residues in MG53 identified by mass spectrometry (MS) analysis of human MG53 overexpressed in HEK293 cells and the endogenous MG53 from murine skeletal muscle. **B**, Spectra of the phosphorylated peptides in MG53. Lysate from HEK293 cells overexpressing MG53 or murine skeletal muscle was resolved by SDS-PAGE, and the band corresponding to MG53 was excised and subjected to in-gel trypsin digestion, followed by tandem MS. **C**, Alignment of peptides containing phosphorylated amino acid residues among different species, and their positions in the corresponding functional domains in MG53.

Fig. S2: The phosphorylation of S255 is essential for E3 ligase activity of MG53.

A and **B**, Representative Western blots and averaged data showing the ubiquitination of IRS1 in NRVMs expressing wild type, S255A, S255D, S255E mutant, or ΔRING truncation of MG53 (n = 6). **C**, Representative Western blots and averaged data showing S255A mutant abrogated MG53-mediated downregulation of IRβ and IRS1 in C2C12 myotubes (n = 5). **D**, Representative Western blots and averaged data showing that S255A mutant abrogated MG53-mediated suppression of insulin-induced phosphorylation of Akt at serine 473 in C2C12 myotubes (n = 6). **E**, Statistical data showing that S255A mutant abrogated MG53-mediated suppression of glucose uptake in C2C12 myotubes (n = 4). Normal distribution was confirmed by Shapiro-Wilk test. Data were analyzed using one-way ANOVA with Tukey post hoc test (**B** and **D**) and the Mann-Whitney *U* test (**C** and **E**). Data are presented as mean ± SEM.

Fig. S3: Identification of the kinase that phosphorylates MG53 at S255.

A, The top 5 kinases predicted by NetPhos 3.1 Server that may phosphorylate human MG53 at S255. **B**, The kinases identified by two independent IP-MS concurrently that interact with MG53 in mouse skeletal muscle. **C**, Spectra of GSK3β peptides identified by IP-MS in the immunocomplex precipitated by anti-MG53 antibody in the lysate from HEK293 cells overexpressing human MG53.

Fig. S4: GSK3β phosphorylated MG53-WT but not MG53-S255A mutant *in vitro*.

A, *In vitro* kinase assay showing the regulation of MG53 phosphorylation by GSK3β (n = 3). **B**, *In vitro* kinase assay showing MG53-S255A mutant could not be phosphorylated by GSK3β (n = 3).

Fig. S5: Development of a monoclonal antibody specifically against phosphorylated MG53 at S255.

A, Representative Western blots and averaged data showing the antibody against phosphorylated MG53 at S255 (anti-p-$MG53^{S255}$) could detect MG53-WT, but not MG53-S255A mutant, in HEK293 cells. Myc-tagged MG53-WT or MG53-S255A mutant was expressed in HEK293 cells and precipitated by anti-Myc antibody, and then detected with anti-p-$MG53^{S255}$ by Western blotting (n = 3). **B**, Representative Western blots and averaged data showing pretreatment with λ protein phosphatase eliminated the signal of phosphorylated MG53 detected by anti-p-$MG53^{S255}$

(n = 5). **C** and **D**, Representative Western blots and averaged data showing that phosphorylated peptide (CLQKILSE-pS-PPPARL) (**C**, n = 6), but not the non-phosphorylated one with the same amino acid sequence (CLQKILSEPPPARL) (**D**, n = 4), dose dependently attenuated the signal intensity of phosphorylated MG53 at S255 in the lysate of HEK293 cells overexpressing MG53. Data are presented as mean ± SEM, and statistical analysis was performed by the Mann-Whitney *U* test (**A** and **B**), one-way ANOVA with Tukey post-test (**C**) and the Kruskal-Wallis test (**D**).

Fig. S6: S255 mutant MG53-S255A, S255D or S255E protects cardiac myocytes from hypoxia-induced cell death.

A and **B**, Cell viability of NRVMs assessed by LDH release in the medium (**A**) and Cell Counting Kit-8 (**B**). Cells were infected with adenovirus expressing β-gal, MG53-WT, MG53-S255A, MG53-S255D, or MG53-S255E, and then subjected to hypoxia (n = 6). Normal distribution was confirmed by Shapiro-Wilk test. Data were analyzed using two-way ANOVA with Sidak's multiple comparisons test (**A**) and the Kruskal-Wallis test (**B**). Data are presented as mean ± SEM.

Fig. S7: S255A mutant does not affect the secretion of MG53.

A and **B**, Representative Western blots (**A**) and averaged data (**B**) showing that the amount of MG53 detected in the medium was similar when wild type MG53, or S255A, S255D, S255E mutant was overexpressed in NRVMs. (n = 5). **C**, Blood glucose levels of 12-week-old *db/+* mice after *i.p.* injection with HSA, rhMG53-WT or rhMG53-S255A (**left panel**, n = 6) or in insulin tolerance tests after administration of indicated recombinant proteins (**right panel**, n = 9 in HSA

group, n = 6 in rhMG53-WT or rhMG53-S255A group). The nonspecific bands (NS) were stained by brilliant green. Normal distribution was confirmed by Shapiro-Wilk test. Data were analyzed using the Kruskal-Wallis test (**B**). Data are presented as mean ± SEM.

Fig. S8: Metabolic data of *db/db* and *db/+* mice.

A-**C**, Body weight (**A**), fasting (**B**) and fed (**C**) blood glucose levels of 12-week-old *db/+* mice, 8-week-old and 12-week-old *db/db* mice (n = 10 for 12-week-old *db/+* mice, n = 19 for 8-week-old *db/db* mice, and n = 21 for 12-week-old *db/db* mice). Normal distribution was confirmed by Shapiro-Wilk test. Data were analyzed using the Kruskal-Wallis test (**A**) and one-way ANOVA with Tukey post hoc test (**B** and **C**). Data are presented as mean ± SEM.

Fig. S9: Pharmacokinetics of MG53 recombinant proteins in mouse plasma.

A, Standard curve of rhMG53-WT and rhMG53-S255A detected by ELISA kit. **B**, The circulating MG53 levels at the indicated time points in the wild type C57/BL6N mice after injected with rhMG53-WT or rhMG53-S255A (1 mg/kg body weight, intravenously injection) were determined by the ELISA assay.

Fig. S10: rhMG53-S255A protects against I/R injury in the heart of rat.

A, Diagram showing the experimental procedures of I/R and treatment with recombinant proteins in mice. **B**, Representative images and quantitative data of area at risk (AAR), infarct size (IF) and serum LDH level from male rats subjected to I/R injury (similar procedure as in (A), except for ischemia for 45 min), and treated with HSA, rhMG53-WT, or rhMG53-S255A (n = 14 in HSA group, n = 13 in rhMG53-WT group, and n = 7 in rhMG53-S255A group). Scale bar, 2 mm. **C**, TUNEL staining of cardiac sections in the rat heart (n = 6 for each group). Scale bar, 20 μm. Normal distribution was confirmed by Shapiro-Wilk test. Data were analyzed using one-way ANOVA with Tukey post hoc test (**B** IF/AAR and **C**) and the Kruskal-Wallis test (**B** LDH). Data are presented as mean ± SEM.

Fig. S11: The experimental procedures of I/R and treatment with recombinant proteins in mice.

A and **B**, Diagram showing the experimental procedures of I/R and Post-ischemia treatment (**A**) and evaluation of long-term effects of treatment (**B**) with recombinant proteins in mice.

Fig. S12: MG53-S255A mutant protected Diabetic heart against I/R induced injury as myokine secreted from skeletal muscle.

A, The sequencing results of MG53-S255A$^{Ki/+}$ knock-in mice. **B**, The random blood glucose levels of the STZ-treated mice (n = 5). **C**, The serum MG53 levels of S255A$^{Ki/+}$ and WT mice (n = 5). **D**, Representative Western blots and averaged data showing the same MG53 protein level in the

skeletal muscle of S255A$^{Ki/+}$ and WT mice (n = 5 for each group). Data were analyzed using the Kruskal-Wallis test (B), the Mann-Whitney *U* test (C and D). Data are presented as mean ± SEM.

Fig. S13: TUNEL staining of cardiac sections in the heart 4 weeks after I/R injury.

Representative images of the TUNEL staining of cardiac sections in the heart 4 weeks after I/R injury and with HSA, rhMG53-WT or rhMG53-S255A treatment. Scale bar, 100 μm.

NonStandard Abbreviations and Acronyms	
**MG53**	Mitsugumin 53
**rhMG53-WT**	recombinant human wild type MG53
**ΔRING**	deletion of RING domain
**HSA**	human serum albumin
**I/R**	ischemia/reperfusion
**T2D**	type 2 diabetes
**GSK3β**	Glycogen synthase kinase 3 beta
**IPC**	ischemia preconditioning
**IR-ECD**	extracellular domain of insulin receptor
**PTMs**	post-translational modifications
**NRVMs**	neonatal rat ventricular myocytes
**LDH**	Lactate dehydrogenase
**tPA**	tissue plasminogen activator
**STZ**	streptozotocin

A

Sample Species	Peptides Counts<br>Phosphorylated : Nonphosphorylated			
	S2/S13	S189	S255	S307
Human	20/14	11/198	18/182	4/83
Mouse	-	0/19	4/51	4/14

B

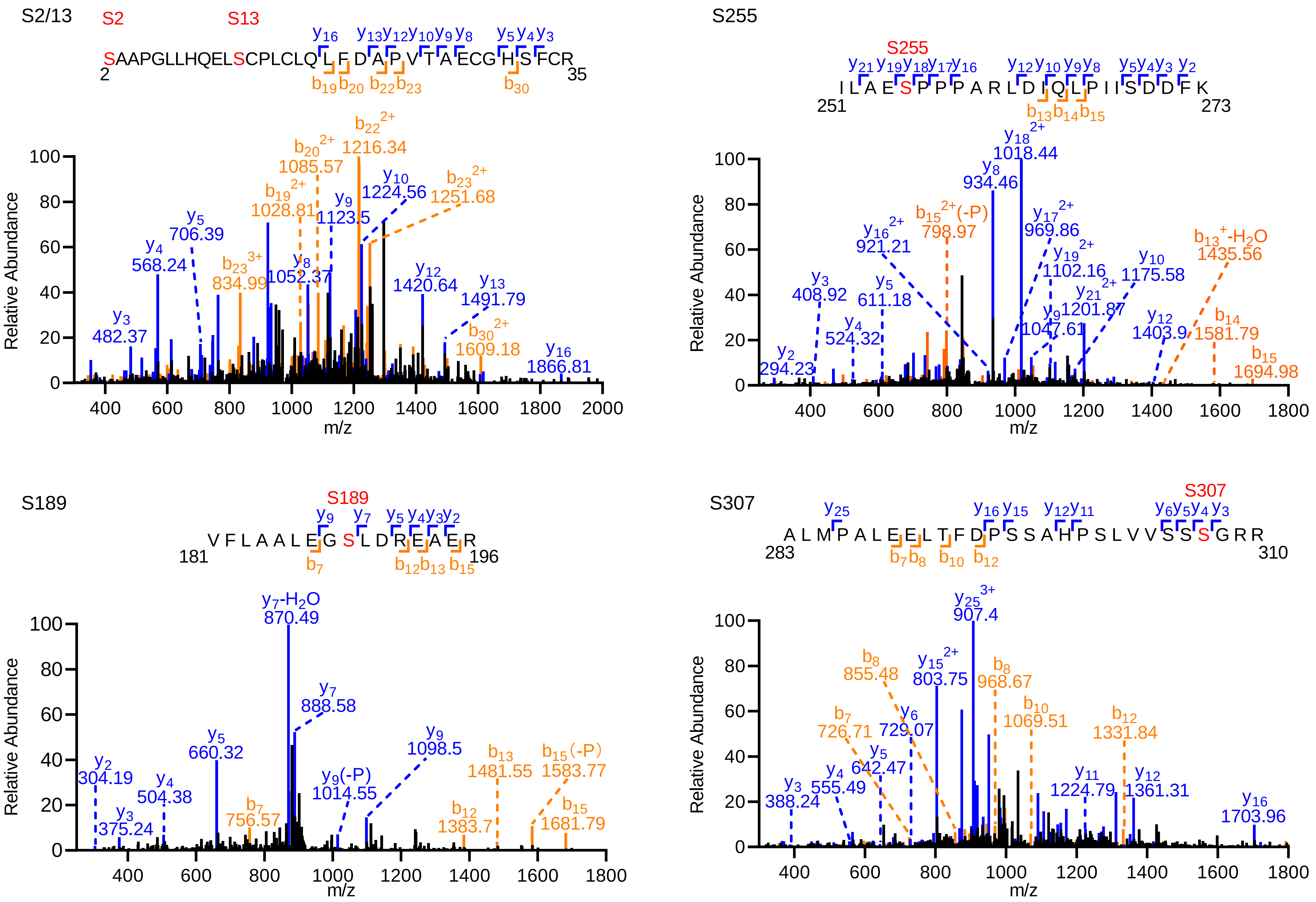

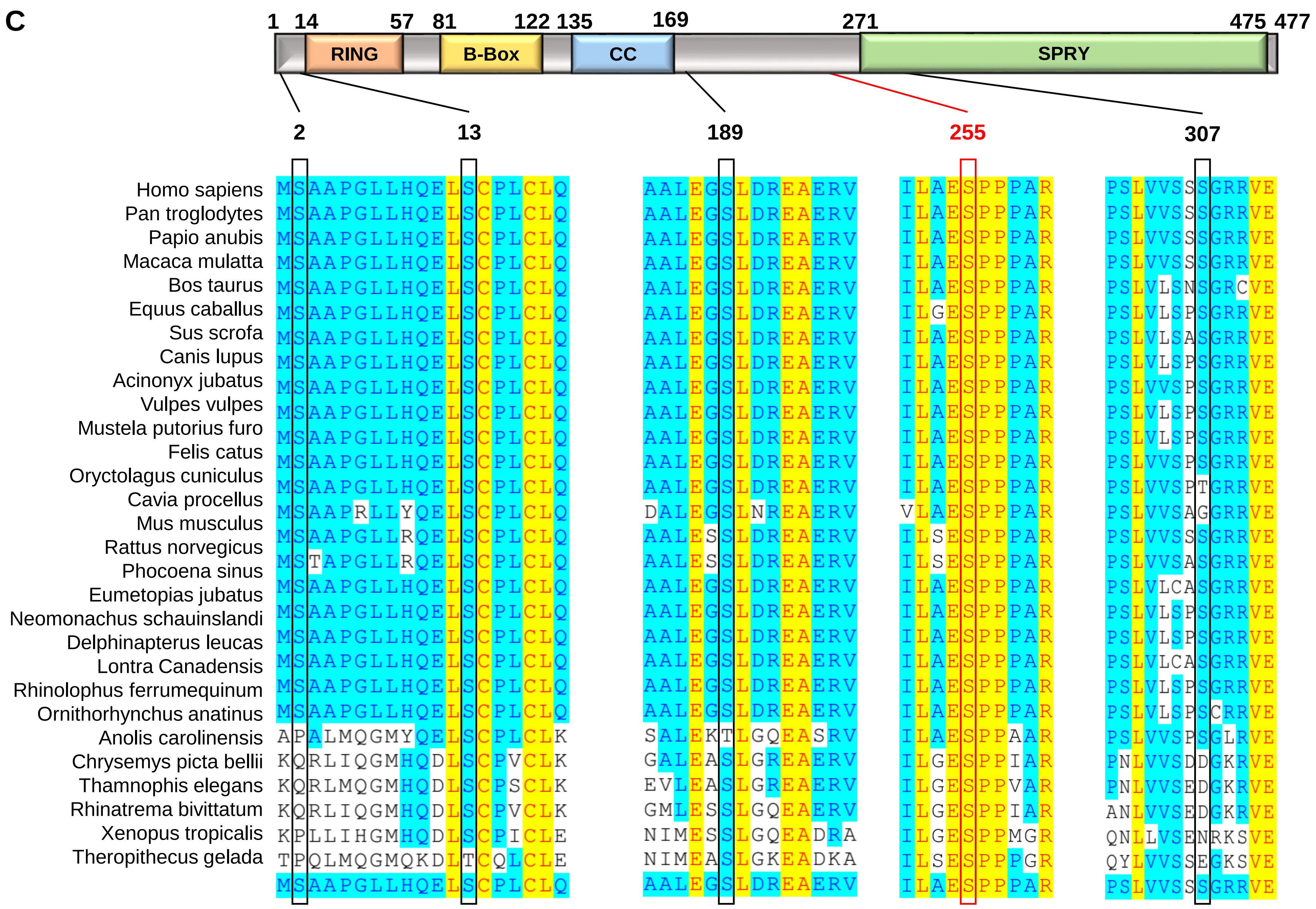
C
1 14 57 81 122 135 169 271 475 477
RING
B-Box
CC
SPRY
2
13
189
255
307
Homo sapiens
Pan troglodytes
Papio anubis
Macaca mulatta
Bos taurus
Equus caballus
Sus scrofa
Canis lupus
Acinonyx jubatus
Vulpes vulpes
Mustela putorius furo
Felis catus
Oryctolagus cuniculus
Cavia procellus
Mus musculus
Rattus norvegicus
Phocoena sinus
Eumetopias jubatus
Neomonachus schauinslandi
Delphinapterus leucas
Lontra Canadensis
Rhinolophus ferrumequinum
Ornithorhynchus anatinus
Anolis carolinensis
Chrysemys picta bellii
Thamnophis elegans
Rhinatrema bivittatum
Xenopus tropicalis
Theropithecus gelada

Fig. S1: Identification of phosphorylation sites in MG53.

A, Spectral counts of peptides containing indicated phosphorylated amino acid residues in MG53 identified by mass spectrometry (MS) analysis of human MG53 overexpressed in HEK293 cells and the endogenous MG53 from murine skeletal muscle. **B**, Spectra of the phosphorylated peptides in MG53. Lysate from HEK293 cells overexpressing MG53 or murine skeletal muscle was resolved by SDS-PAGE, and the band corresponding to MG53 was excised and subjected to in-gel trypsin digestion, followed by tandem MS. **C**, Alignment of peptides containing phosphorylated amino acid residues among different species, and their positions in the corresponding functional domains in MG53.

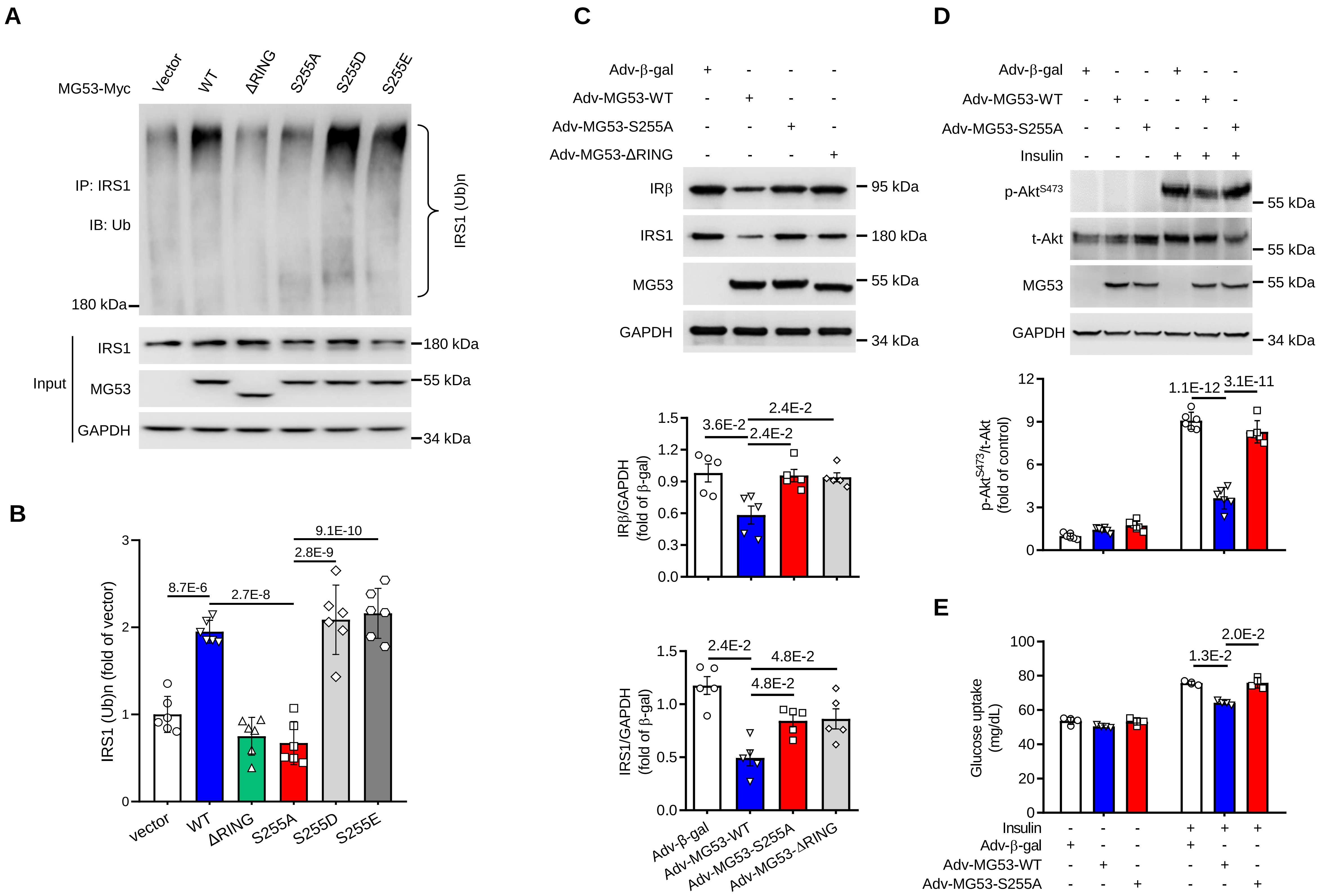
A
MG53-Myc
Vector
WT
ΔRING
S255A
S255D
S255E
IP: IRS1
IB: Ub
IRS1 (Ub)n
180 kDa
Input
IRS1
MG53
GAPDH
180 kDa
55 kDa
34 kDa
B
IRS1 (Ub)n (fold of vector)
8.7E-6
2.7E-8
2.8E-9
9.1E-10
vector
WT
ΔRING
S255A
S255D
S255E
C
Adv-β-gal
Adv-MG53-WT
Adv-MG53-S255A
Adv-MG53-ΔRING
IRβ
IRS1
MG53
GAPDH
95 kDa
180 kDa
55 kDa
34 kDa
IRβ/GAPDH (fold of β-gal)
3.6E-2
2.4E-2
2.4E-2
IRS1/GAPDH (fold of β-gal)
2.4E-2
4.8E-2
4.8E-2
D
Adv-β-gal
Adv-MG53-WT
Adv-MG53-S255A
Insulin
p-AktS473
t-Akt
MG53
GAPDH
55 kDa
55 kDa
55 kDa
34 kDa
p-AktS473/t-Akt (fold of control)
1.1E-12
3.1E-11
E
Glucose uptake (mg/dL)
1.3E-2
2.0E-2
Insulin
Adv-β-gal
Adv-MG53-WT
Adv-MG53-S255A

Fig. S2: The phosphorylation of S255 is essential for E3 ligase activity of MG53.

A and **B**, Representative Western blots and averaged data showing the ubiquitination of IRS1 in NRVMs expressing wild type, S255A, S255D, S255E mutant, or ΔRING truncation of MG53 (n = 6). **C**, Representative Western blots and averaged data showing S255A mutant abrogated MG53-mediated downregulation of IRβ and IRS1 in C2C12 myotubes (n = 5). **D**, Representative Western blots and averaged data showing that S255A mutant abrogated MG53-mediated suppression of insulin-induced phosphorylation of Akt at serine 473 in C2C12 myotubes (n = 6). **E**, Statistical data showing that S255A mutant abrogated MG53-mediated suppression of glucose uptake in C2C12 myotubes (n = 4). Normal distribution was confirmed by Shapiro-Wilk test. Data were analyzed using one-way ANOVA with Tukey post hoc test (**B** and **D**) and the Mann-Whitney *U* test (**C** and **E**). Data are presented as mean ± SEM.

A

Predicted kinases			
**Rank**	**Score**	**Kinase**	**Answer**
1	0.965	unsp	Yes
**2**	**0.508**	**GSK3**	**Yes**
3	0.483	cdc2	-
4	0.435	cdk5	-
5	0.424	CaMK-II	-

B

Kinases identified by LC-MS	
**Rank**	**Kinase**
1	PGK1
2	KCRM
3	BCKD
**4**	**GSK3β**
5	PKLR

C

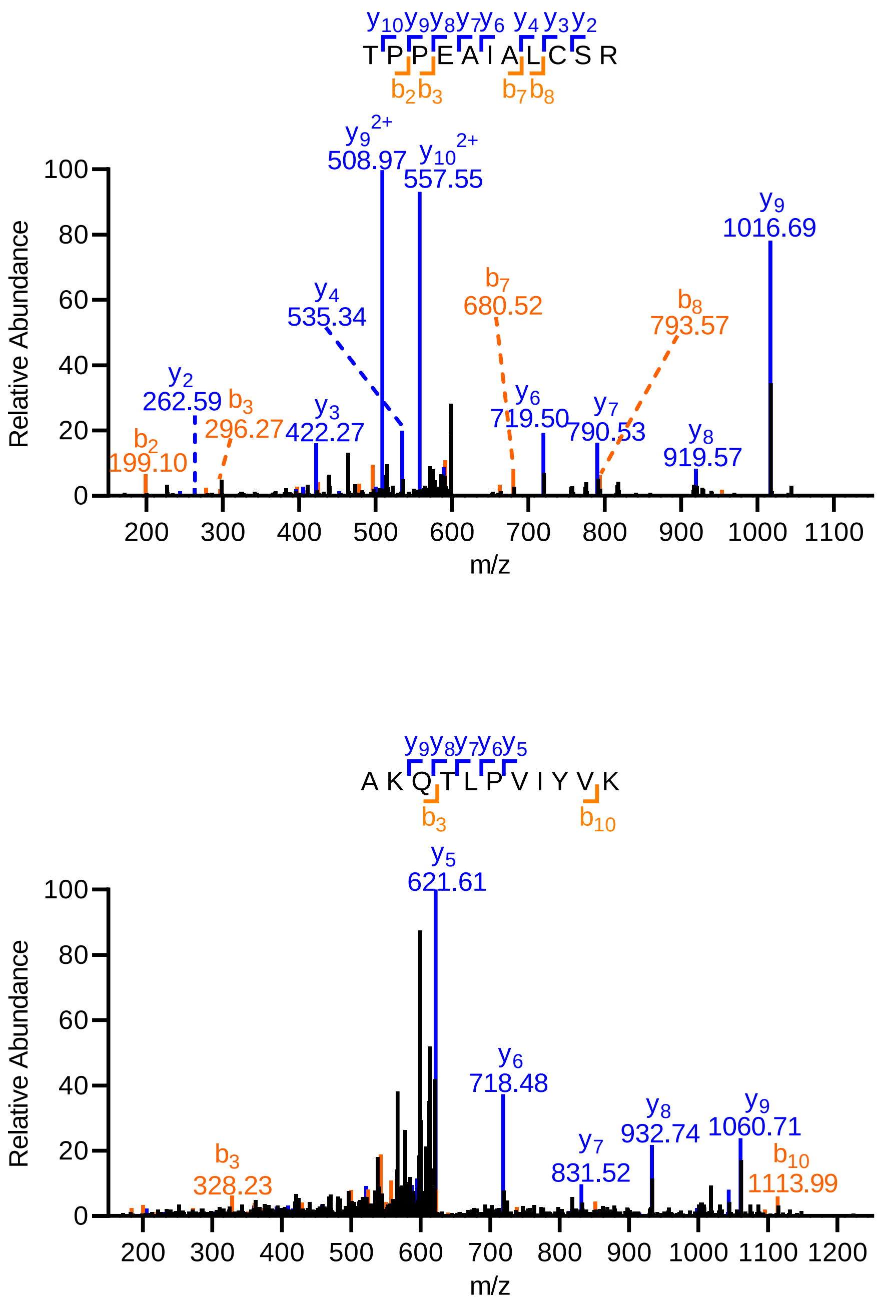

Fig. S3: Identification of the kinase that phosphorylates MG53 at S255.

A, The top 5 kinases predicted by NetPhos 3.1 Server that may phosphorylate human MG53 at S255. **B**, The kinases identified by two independent IP-MS concurrently that interact with MG53 in mouse skeletal muscle. **C**, Spectra of GSK3β peptides identified by IP-MS in the immunocomplex precipitated by anti-MG53 antibody in the lysate from HEK293 cells overexpressing human MG53.

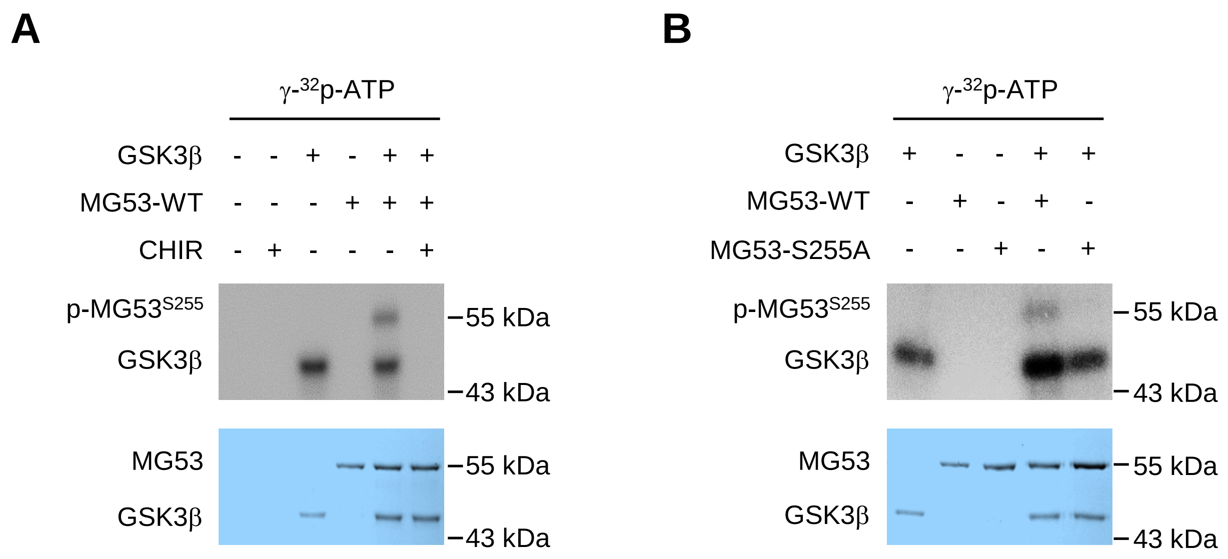
A
γ-32p-ATP
GSK3β - - + - + +
MG53-WT - - - + + +
CHIR - + - - - +
p-MG53S255
GSK3β
55 kDa
43 kDa
MG53
GSK3β
55 kDa
43 kDa
B
γ-32p-ATP
GSK3β + - - + +
MG53-WT - + - + -
MG53-S255A - - + - +
p-MG53S255
GSK3β
55 kDa
43 kDa
MG53
GSK3β
55 kDa
43 kDa

Fig. S4: GSK3β phosphorylated MG53-WT but not MG53-S255A mutant in vitro.

A, *In vitro* kinase assay showing the regulation of MG53 phosphorylation by GSK3β (n = 3). **B**, *In vitro* kinase assay showing MG53-S255A mutant could not be phosphorylated by GSK3β (n = 3).

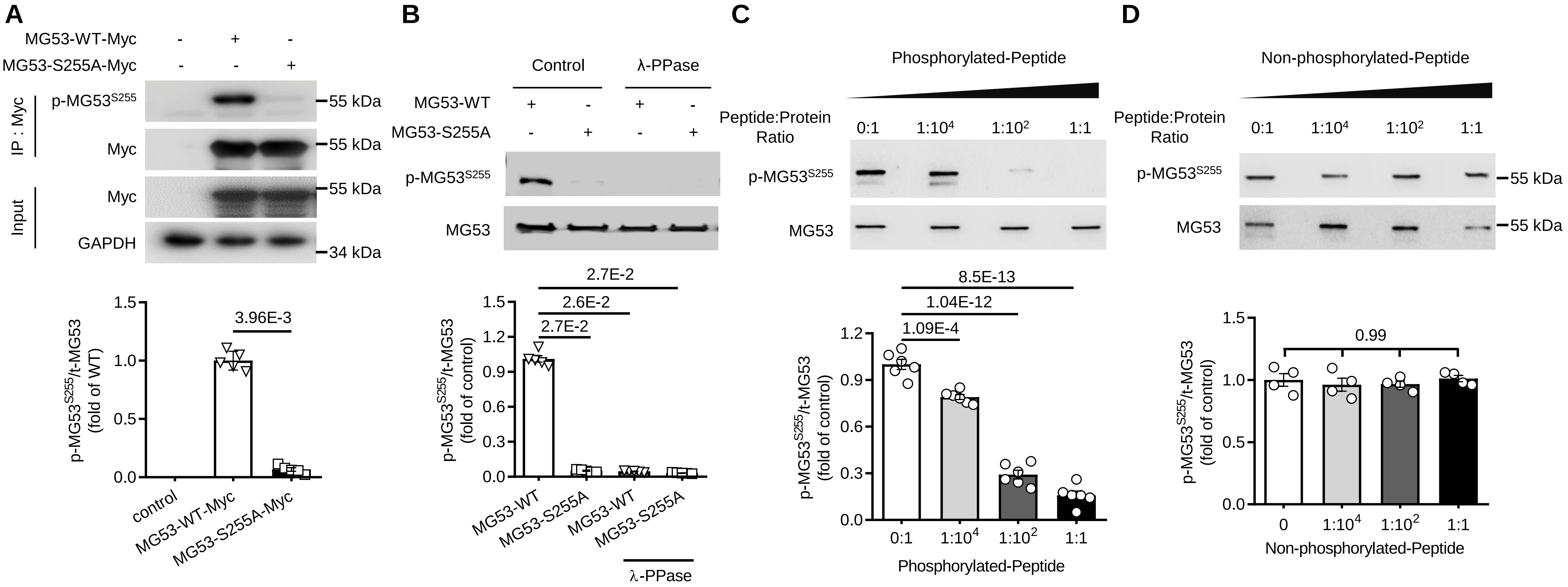

A
MG53-WT-Myc
MG53-S255A-Myc
IP : Myc
Input
p-MG53S255
Myc
Myc
GAPDH
55 kDa
55 kDa
55 kDa
34 kDa
3.96E-3
p-MG53S255/t-MG53 (fold of WT)
control
MG53-WT-Myc
MG53-S255A-Myc
B
Control
λ-PPase
MG53-WT
MG53-S255A
p-MG53S255
MG53
2.7E-2
2.6E-2
2.7E-2
p-MG53S255/t-MG53 (fold of control)
MG53-WT
MG53-S255A
MG53-WT
MG53-S255A
λ-PPase
C
Phosphorylated-Peptide
Peptide:Protein Ratio
0:1
1:10^4
1:10^2
1:1
p-MG53S255
MG53
8.5E-13
1.04E-12
1.09E-4
p-MG53S255/t-MG53 (fold of control)
Phosphorylated-Peptide
D
Non-phosphorylated-Peptide
Peptide:Protein Ratio
p-MG53S255
MG53
55 kDa
55 kDa
0.99
p-MG53S255/t-MG53 (fold of control)
0
Non-phosphorylated-Peptide

Fig. S5: Development of a monoclonal antibody specifically against phosphorylated MG53 at S255.

A, Representative Western blots and averaged data showing the antibody against phosphorylated MG53 at S255 (anti-p-MG53^{S255}) could detect MG53-WT, but not MG53-S255A mutant, in HEK293 cells. Myc-tagged MG53-WT or MG53-S255A mutant was expressed in HEK293 cells and precipitated by anti-Myc antibody, and then detected with anti-p-MG53^{S255} by Western blotting (n = 5). **B**, Representative Western blots and averaged data showing pretreatment with λ protein phosphatase eliminated the signal of phosphorylated MG53 detected by anti-p-MG53^{S255} (n = 5). **C** and **D**, Representative Western blots and averaged data showing that phosphorylated peptide (CLQKILSE-pS-PPPARL) (**C**, n = 6), but not the non-phosphorylated one with the same amino acid sequence (CLQKILSEPPPARL) (**D**, n = 4), dose dependently attenuated the signal intensity of phosphorylated MG53 at S255 in the lysate of HEK293 cells overexpressing MG53. Data are presented as mean ± SEM, and statistical analysis was performed by the Mann-Whitney *U* test (**A** and **B**), one-way ANOVA with Tukey post-test (**C**) and the Kruskal-Wallis test (**D**).

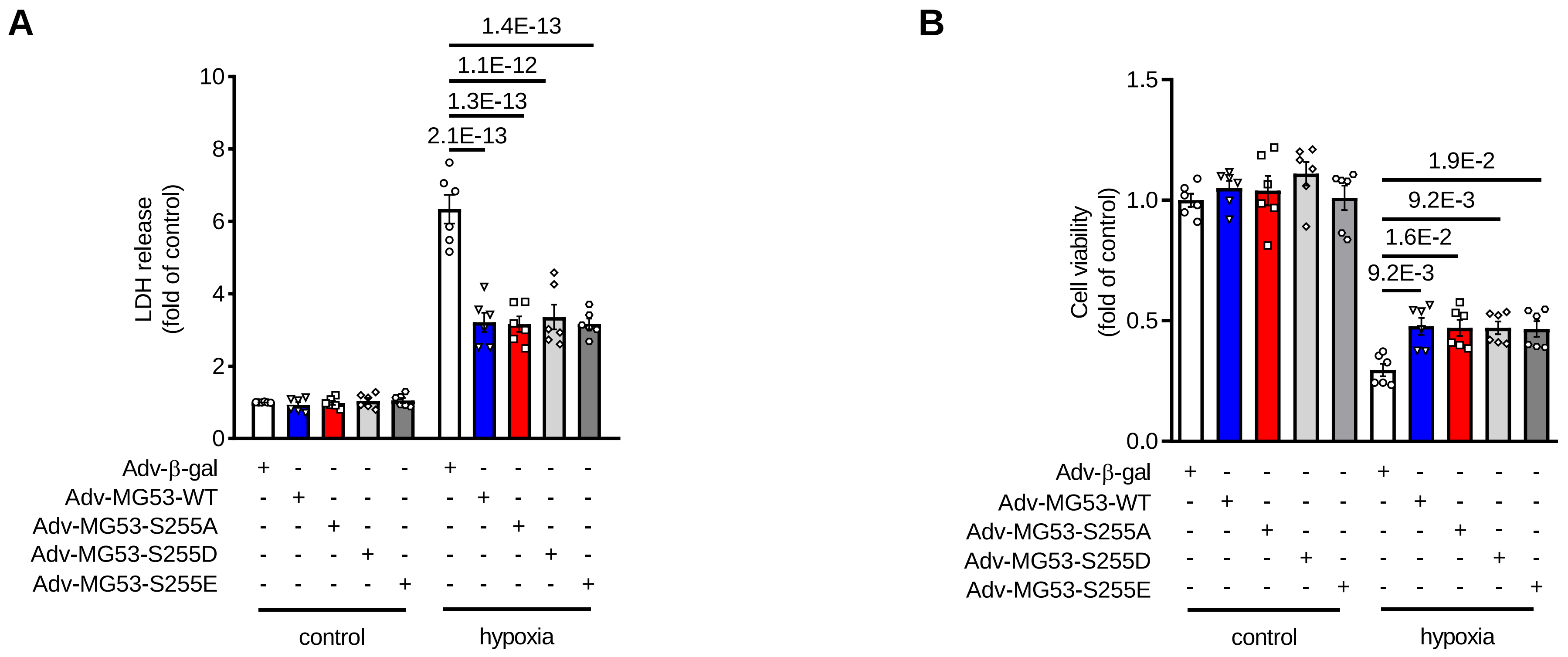

A
1.4E-13
1.1E-12
1.3E-13
2.1E-13
LDH release
(fold of control)
10
8
6
4
2
0
Adv-β-gal
Adv-MG53-WT
Adv-MG53-S255A
Adv-MG53-S255D
Adv-MG53-S255E
control
hypoxia
B
Cell viability
(fold of control)
1.5
1.0
0.5
0.0
1.9E-2
9.2E-3
1.6E-2
9.2E-3
Adv-β-gal
Adv-MG53-WT
Adv-MG53-S255A
Adv-MG53-S255D
Adv-MG53-S255E
control
hypoxia

Fig. S6: S255 site mutant MG53-S255A, S255D or S255E protect cardiac myocytes from hypoxia-induced cell death.

A and **B**, Cell viability of NRVMs assessed by LDH release in the medium (**A**) and Cell Counting Kit-8 (**B**). Cells were infected with adenovirus expressing β-gal, MG53-WT, MG53-S255A, MG53-S255D, or MG53-S255E, and then subjected to hypoxia (n = 6). Normal distribution was confirmed by Shapiro-Wilk test. Data were analyzed using two-way ANOVA with Sidak's multiple comparisons test (**A**) and the Kruskal-Wallis test (**B**). Data are presented as mean ± SEM.

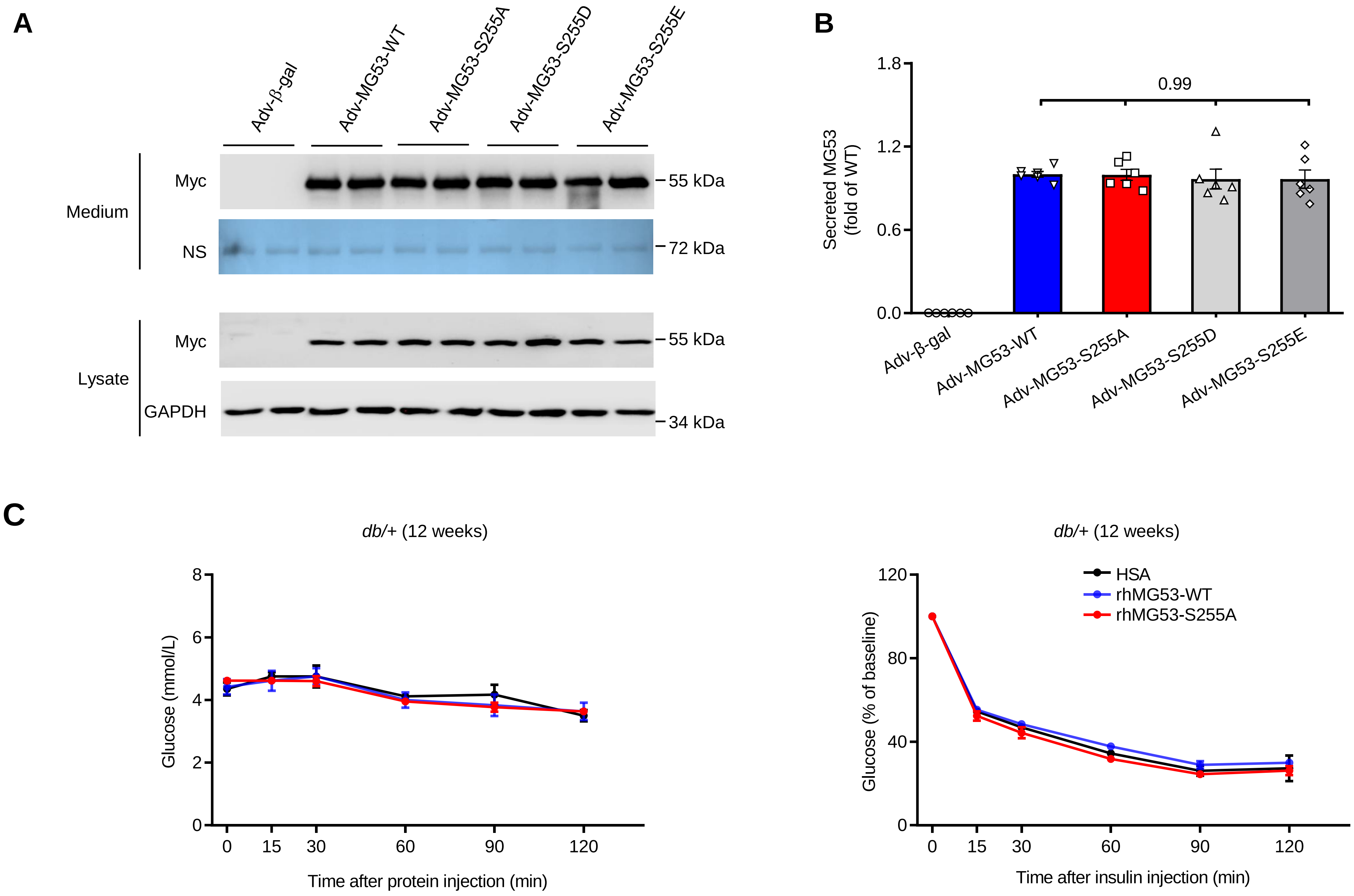
A
Adv-β-gal
Adv-MG53-WT
Adv-MG53-S255A
Adv-MG53-S255D
Adv-MG53-S255E
Medium
Myc
NS
Lysate
Myc
GAPDH
55 kDa
72 kDa
55 kDa
34 kDa
B
Secreted MG53 (fold of WT)
0.99
C
db/+ (12 weeks)
Glucose (mmol/L)
Time after protein injection (min)
db/+ (12 weeks)
Glucose (% of baseline)
Time after insulin injection (min)
HSA
rhMG53-WT
rhMG53-S255A

Fig. S7: S255A mutant does not affect the secretion of MG53.

A and **B**, Representative Western blots (**A**) and averaged data (**B**) showing that the amount of MG53 detected in the medium was similar when wild type MG53, or S255A, S255D, S255E mutant was overexpressed in NRVMs. (n = 5). **C**, Blood glucose levels of 12-week-old *db/+* mice after *i.p.* injection with HSA, rhMG53-WT or rhMG53-S255A (**left panel**, n = 6) or in insulin tolerance tests after administration of indicated recombinant proteins (**right panel**, n = 9 in HSA group, n = 6 in rhMG53-WT or rhMG53-S255A group). The nonspecific bands (NS) were stained by brilliant green. Normal distribution was confirmed by Shapiro-Wilk test. Data were analyzed using the Kruskal-Wallis test (**B**). Data are presented as mean ± SEM.

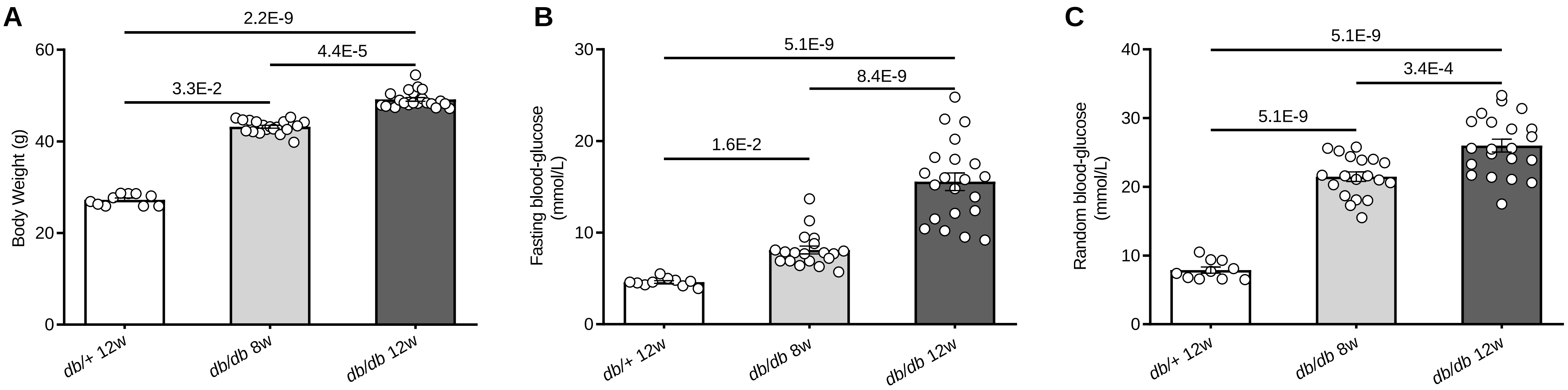
A
2.2E-9
4.4E-5
3.3E-2
Body Weight (g)
60
40
20
0
db/+ 12w
db/db 8w
db/db 12w
B
5.1E-9
8.4E-9
1.6E-2
Fasting blood-glucose (mmol/L)
30
20
10
0
db/+ 12w
db/db 8w
db/db 12w
C
5.1E-9
3.4E-4
5.1E-9
Random blood-glucose (mmol/L)
40
30
20
10
0
db/+ 12w
db/db 8w
db/db 12w

Fig. S8: Metabolic data of *db/db* and *db/+* mice.

A-C, Body weight (**A**), fasting (**B**) and fed (**C**) blood glucose levels of 12-week-old *db/+* mice, 8-week-old and 12-week-old *db/db* mice (n = 10 for 12-week-old *db/+* mice, n = 19 for 8-week-old *db/db* mice, and n = 21 for 12-week-old *db/db* mice). Normal distribution was confirmed by Shapiro-Wilk test. Data were analyzed using the Kruskal-Wallis test (**A**) and one-way ANOVA with Tukey post hoc test (**B** and **C**). Data are presented as mean ± SEM.

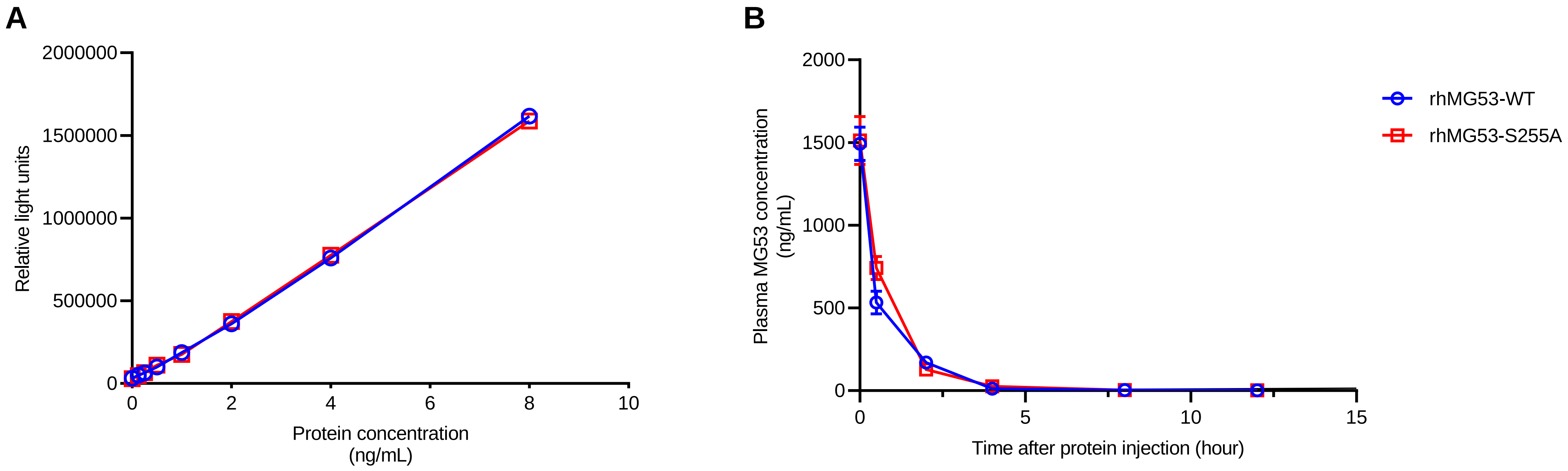
A
2000000
1500000
1000000
500000
0
Relative light units
0
2
4
6
8
10
Protein concentration
(ng/mL)
B
2000
1500
1000
500
0
Plasma MG53 concentration
(ng/mL)
0
5
10
15
Time after protein injection (hour)
rhMG53-WT
rhMG53-S255A

Fig. S9: Pharmacokinetics of MG53 recombinant proteins in mouse plasma.

A, Standard curve of rhMG53-WT and rhMG53-S255A detected by ELISA kit. **B**, The circulating MG53 levels at the indicated time points in the wild type C57/BL6N mice after injected with rhMG53-WT or rhMG53-S255A (1 mg/kg body weight, intravenously injection) were determined by the ELISA assay.

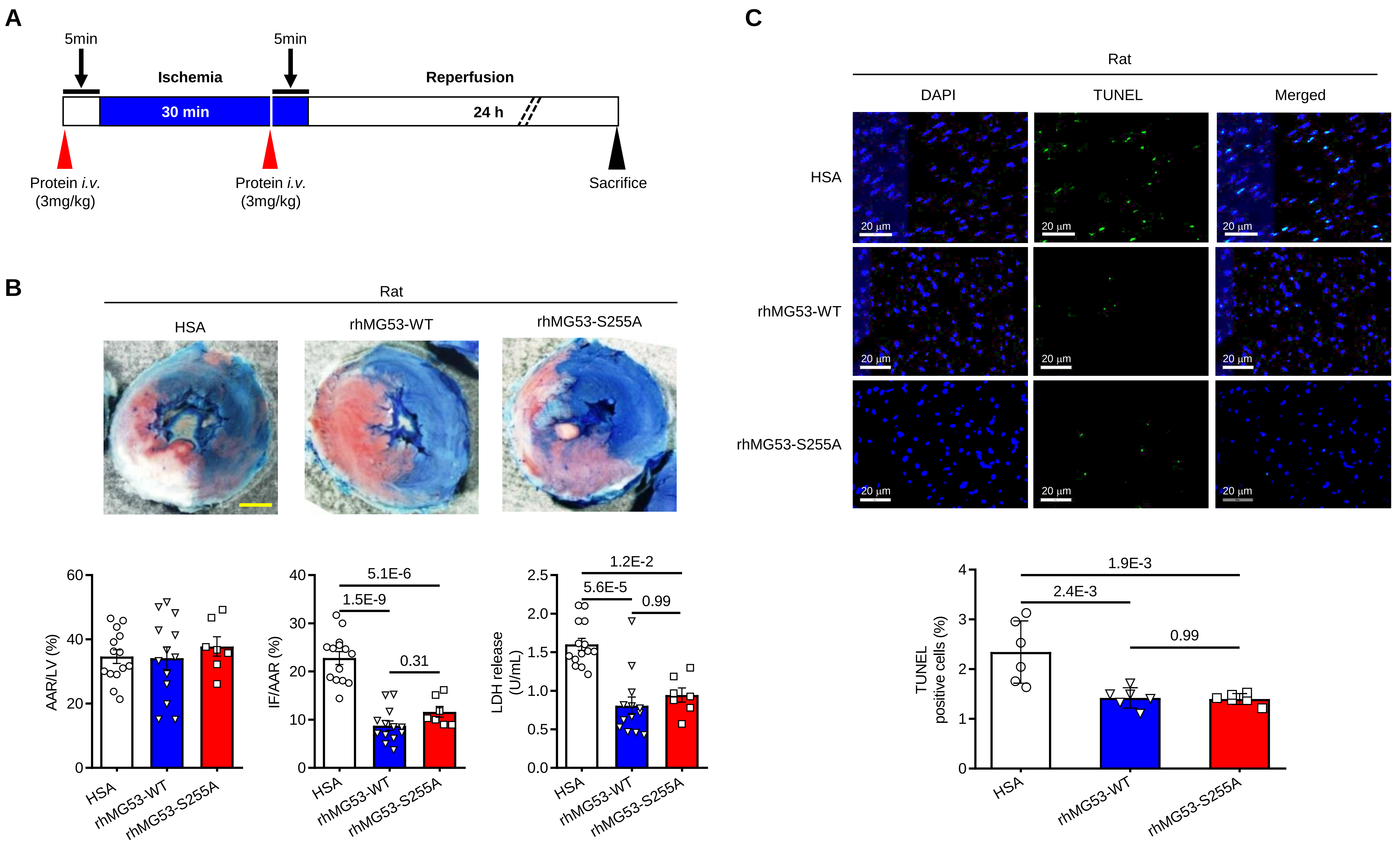
A
5min
5min
Ischemia
Reperfusion
30 min
24 h
Protein i.v. (3mg/kg)
Protein i.v. (3mg/kg)
Sacrifice
B
Rat
HSA
rhMG53-WT
rhMG53-S255A
AAR/LV (%)
IF/AAR (%)
LDH release (U/mL)
5.1E-6
1.5E-9
0.31
1.2E-2
5.6E-5
0.99
C
Rat
DAPI
TUNEL
Merged
20 µm
TUNEL positive cells (%)
1.9E-3
2.4E-3
0.99

Fig. S10: rhMG53-S255A protects against I/R injury in the heart of rat.

A, Diagram showing the experimental procedures of I/R and treatment with recombinant proteins in mice. **B**, Representative images and quantitative data of area at risk (AAR), infarct size (IF) and serum LDH level from male rats subjected to I/R injury (similar procedure as in (A), except for ischemia for 45 min), and treated with HSA, rhMG53-WT, or rhMG53-S255A (n = 14 in HSA group, n = 13 in rhMG53-WT group, and n = 7 in rhMG53-S255A group). Scale bar, 2 mm. **C**, TUNEL staining of cardiac sections in the rat heart (n = 6 for each group). Scale bar, 20 μm. Normal distribution was confirmed by Shapiro-Wilk test. Data were analyzed using one-way ANOVA with Tukey post hoc test (**B** IF/AAR and **C**) and the Kruskal-Wallis test (**B** LDH). Data are presented as mean ± SEM.

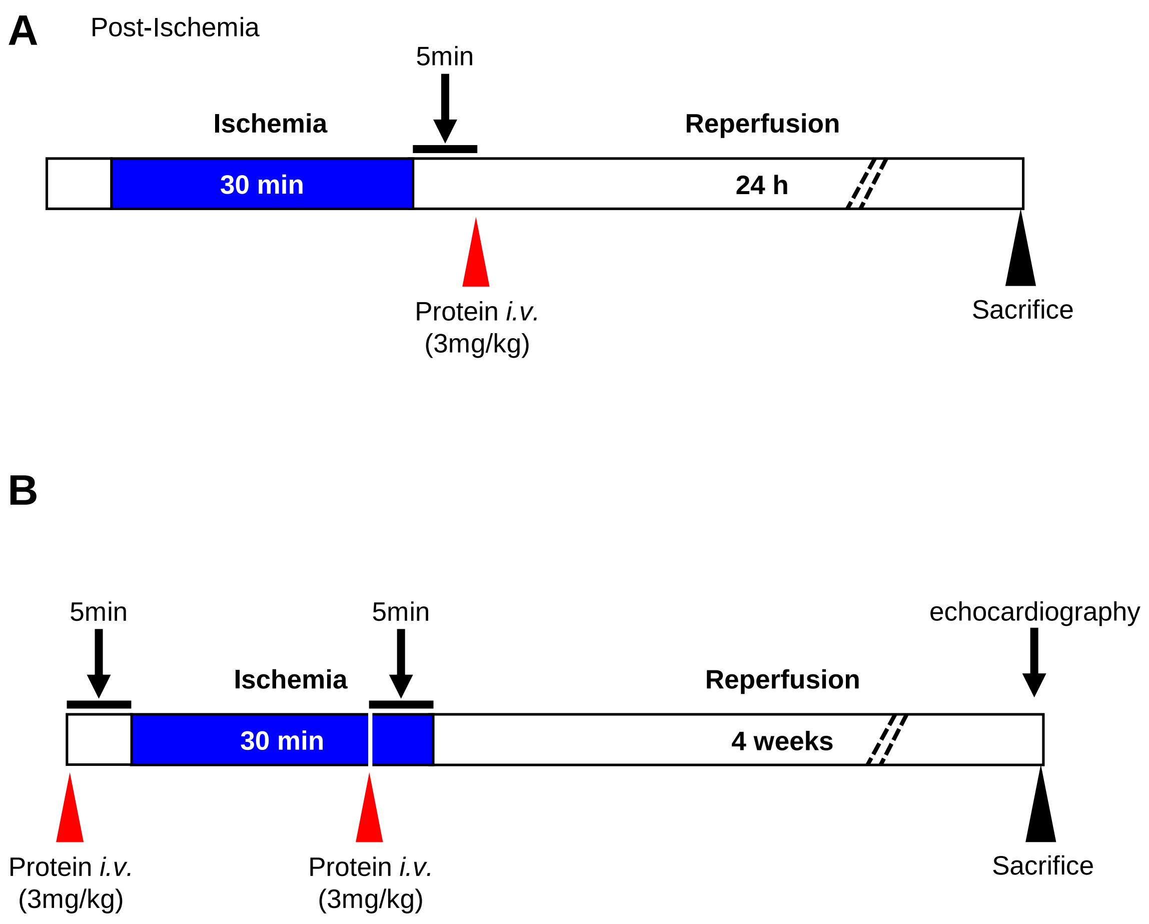
A
Post-Ischemia
5min
Ischemia
Reperfusion
30 min
24 h
Protein *i.v.*
(3mg/kg)
Sacrifice
B
5min
5min
echocardiography
Ischemia
Reperfusion
30 min
4 weeks
Protein *i.v.*
(3mg/kg)
Protein *i.v.*
(3mg/kg)
Sacrifice

Fig. S11: The experimental procedures of I/R and treatment with recombinant proteins in mice.

A and **B**, Diagram showing the experimental procedures of I/R and Post-ischemia treatment (**A**) and evaluation of long-term effects of treatment (**B**) with recombinant proteins in mice.

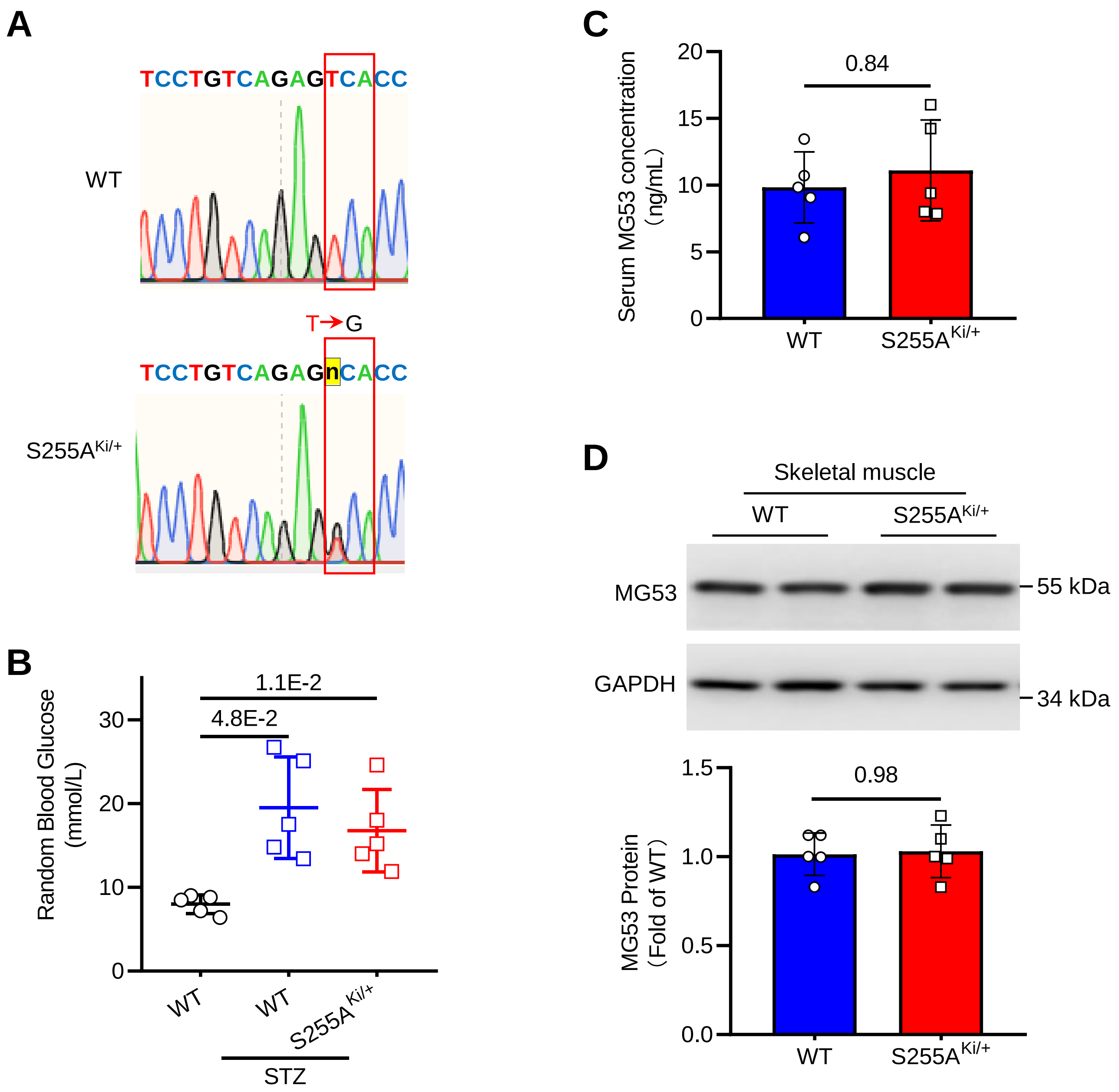

A
TCCTGTCAGAGTCACC
WT
T→G
TCCTGTCAGAGnCACC
S255A^Ki/+
B
Random Blood Glucose (mmol/L)
1.1E-2
4.8E-2
WT
WT
S255A^Ki/+
STZ
C
Serum MG53 concentration (ng/mL)
0.84
WT
S255A^Ki/+
D
Skeletal muscle
WT
S255A^Ki/+
MG53
GAPDH
55 kDa
34 kDa
MG53 Protein (Fold of WT)
0.98
WT
S255A^Ki/+

Fig. S12:MG53-S255A mutant protected Diabetic heart against I/R induced injury as Myokine secreted from skeletal muscle.

A, The sequencing results of MG53-S255A$^{Ki/+}$ knock-in mice. **B**, The random blood glucose levels of the STZ-treated mice (n = 5). **C**, The serum MG53 levels of S255A$^{Ki/+}$ and WT mice (n = 5). **D**, Representative Western blots and averaged data showing the same MG53 protein level in the skeletal muscle of S255A$^{Ki/+}$ and WT mice (n = 5 for each group). Data were analyzed using the Kruskal-Wallis test (**B**), the Mann-Whitney *U* test (**C** and **D**). Data are presented as mean ± SEM.

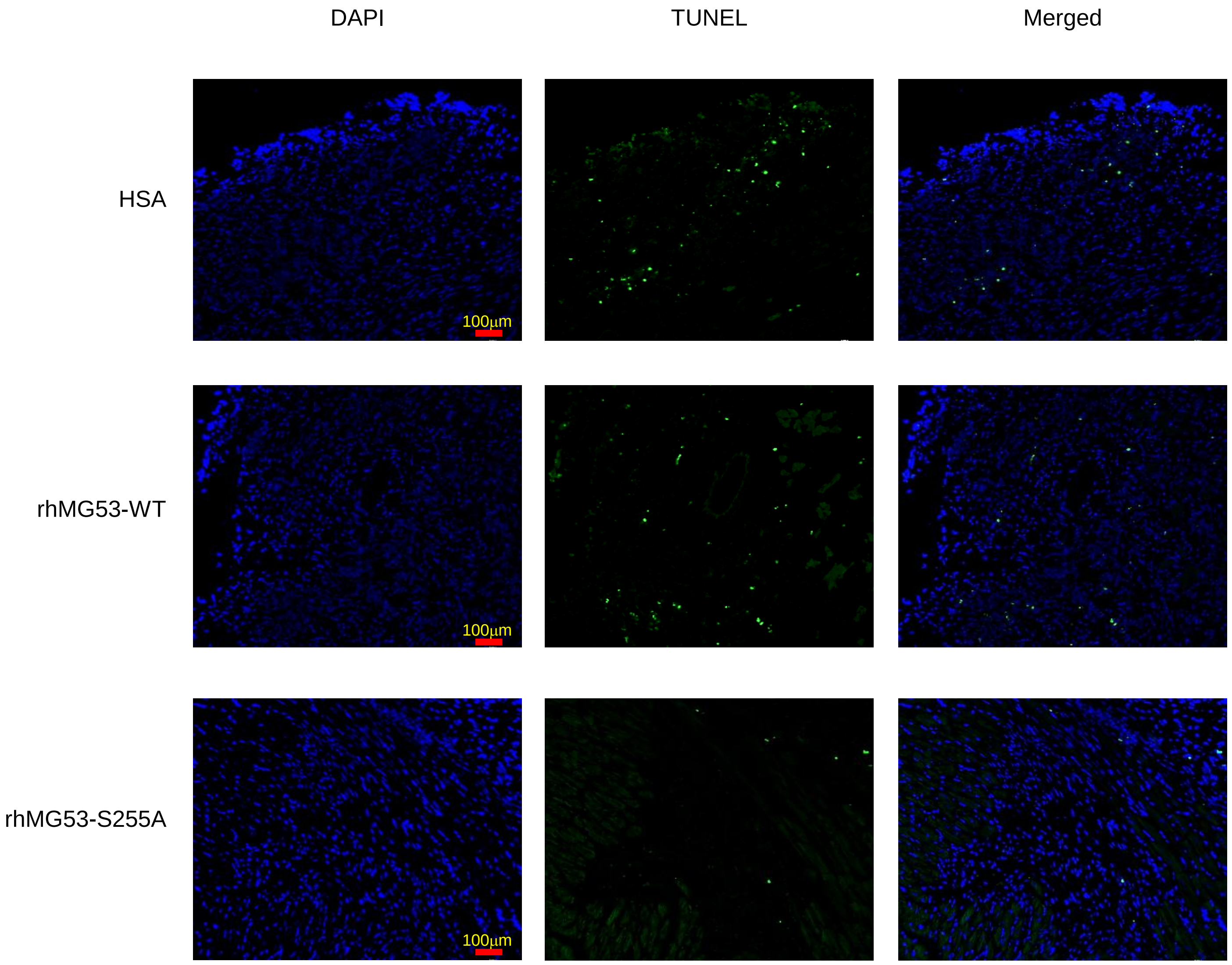
DAPI
TUNEL
Merged
HSA
rhMG53-WT
rhMG53-S255A
100µm
100µm
100µm

Fig. S13: TUNEL staining of cardiac sections in the heart 4 weeks after I/R injury.

Representative images of the TUNEL staining of cardiac sections in the heart 4 weeks after I/R injury and with HSA, rhMG53-WT or rhMG53-S255A treatment. Scale bar, 100 μm.
