# Supplementary material for: Blocking MG53S255 Phosphorylation Protects Diabetic Heart From Ischemic Injury
Source: Circ Res. 2022 Nov 7;131(12):962–76. doi: 10.1161/CIRCRESAHA.122.321055 (PMC9770150; doi:10.1161/CIRCRESAHA.122.321055)
Supplement: Supplementary file 5 [file res-131-0962-s005.pdf]

Major Resources Table

In order to allow validation and replication of experiments, all essential research materials listed in the Methods should be included in the Major Resources Table below. Authors are encouraged to use public repositories for protocols, data, code, and other materials and provide persistent identifiers and/or links to repositories when available. Authors may add or delete rows as needed.

Animals (in vivo studies)

Species	Vendor or Source	Background Strain	Sex	Persistent ID / URL
Rattus norvegicus	Vital River Laboratories	Sprague-Dawley	Male	Cat#101
Rattus norvegicus neonatal	Vital River Laboratories	Sprague-Dawley	N/A	Cat# 734476,
Mouse	Vital River Laboratories	C57BL/6N	Male	https://www.vitalriver.com/#/animalModel/detailedReading?id=20&idd=9&namecode=productserve
Mouse (*db/db* *Lepr*^{em2Cd})	GemPharmatech	C57BLKS/JGpt	Male/Female	Cat#T002407
Nonhuman primate	This paper	N/A	Male	N/A

Genetically Modified Animals

Species	Vendor or Source	Background Strain	Other Information	Persistent ID / URL
Mouse	This paper	C57BL/6J	MG53 S255A mutation knock-in mouse, S255A$^{ki/+}$	N/A
Mouse	Song et al., *Nature*, volume 494:375-379(2013)	C57BL/6J	MG53 knock out mouse, MG53$^{-/-}$	N/A

Antibodies

Target antigen	Vendor or Source	Catalog #	Working concentration	Lot # (preferred but not required)	Persistent ID / URL
Phospho-Akt S473	Cell Signaling Technology	4060	1:1,000		RRID: AB_2315049
Total Akt	Cell Signaling Technology	9272	1:1,000		RRID: AB_329827
Insulin receptor β subunit	Cell Signaling Technology	3025	1:1,000		RRID: AB_2280448
Insulin receptor substrate 1	Cell Signaling Technology	2382	1:1,000		RRID: AB_330333
Total Glycogen synthase kinase-3 beta (GSK3β)	Cell Signaling Technology	9315	1:1,000		RRID: AB_490890
β-Catenin	Cell Signaling Technology	9562	1:1,000		RRID: AB_331149
Myc	Sigma-Aldrich	M5546	1:5,000		RRID: AB_260581
Insulin receptor α subunit	Abcam	ab36550	1:1,000		RRID: AB_775690

DOI [to be added]

Flag	Sigma-Aldrich	F1804	1:5,000		RRID: AB_262044
HA	Santa Cruz	sc-7392	1:1,000		RRID: AB_627809
GAPDH	Bioeasy Technology	BE0023	1:10,000		N/A
Phosphor-GSK3β S9	Invitrogen	MA5-14873	1:1,000		N/A
MG53	Wu et al., *Circulation*, volume 139:901-914(2019)	N/A	1:2,000		N/A
Phospho-MG53 S255	This paper	N/A	1:1,000		N/A

DNA/cDNA Clones

Clone Name	Sequence	Source / Repository	Persistent ID / URL
Plasmid: MG53-S2/13A	Gene ID: 493829, Ser2Ala, Ser13Ala	This Paper	N/A
Plasmid: MG53-S189A	Gene ID: 493829, Ser189Ala	This Paper	N/A
Plasmid: MG53-S255A	Gene ID: 493829, Ser255Ala	This Paper	N/A
Plasmid: MG53-S307A	Gene ID: 493829, Ser307Ala	This Paper	N/A
Plasmid: MG53-WT	Gene ID: 493829	Song et al., *Nature*, volume 494:375-379(2013)	N/A
Plasmid: MG53-ΔRING	Gene ID: 493829, 271-477aa	Song et al., *Nature*, volume 494:375-379(2013)	N/A
Plasmid: pBS mouse IRS1	Gene ID: 16367	Addgene	Cat#11026
Pasmid: pcDNA4/TO/myc-His B	N/A	Invitrogen	Cat#V103020
Plasmid: pcDNA3.1(+)-myc-HisA	N/A	Invitrogen	Cat#V80020
Plasmid: GSK3β–HA	Gene ID: 56637	Sun et al., *J Biol Chem*, volume 287:22882-22888(2012)	N/A
Adenovirus: MG53-WT	Gene ID: 493829	Song et al., *Nature*, volume 494:375-379(2013)	N/A
Adenovirus: MG53-S255A	Gene ID: 493829, Ser255Ala	This Paper	N/A
Adenovirus: MG53-S255E	Gene ID: 493829, Ser255Glu	This Paper	N/A
Adenovirus: MG53-S255D	Gene ID: 493829, Ser255Asp	This Paper	N/A
Adenovirus: MG53-ΔRING	Gene ID: 493829, 271-477aa	Song et al., *Nature*, volume 494:375-379(2013)	N/A
Adenovirus: β-gal	Gene ID: 945006	Zhang et al., *Nature Cell Biology*, volume 21, pages1152–1163 (2019)	N/A
Adenovirus: tPA-MG53-WT	tPA coding sequence: 5’-ATGGATGCAATGAAGAGAGGGCT	Feng et al., *Diabetes*, volume 71:298-314(2022)	N/A

DOI [to be added]

	CTGCTGTGTGCTGCTGCTGTGTGG AGCAGTCTTCGTTTCGCCC-3' Gene ID: 493829		
Adenovirus: tPA-MG53-S255A	tPA coding sequence: 5'-ATGGATGCAATGAAGAGAGGGCT CTGCTGTGTGCTGCTGCTGTGTGG AGCAGTCTTCGTTTCGCCC-3' Gene ID: 493829, Ser255Ala	This paper	N/A

Cultured Cells

Name	Vendor or Source	Sex (F, M, or unknown)	Persistent ID / URL
HEK293 cells	ATCC	unknown	Cat# CRL-1573
Neonatal rat ventricular cardiomyocytes	Vital River Laboratories	unknown	N/A

Other

Description	Source / Repository	Persistent ID / URL
**Protein**		
Human MG53-WT protein	This paper	N/A
Human MG53-S255A protein	This paper	N/A
Human Serum Albumin	Sigma-Aldrich	Cat#70024-90-7
Human insulin receptor extracellular domain protein	ACROBiosystems	Cat#INR-H5220
**Kit**		
Lactate Dehydrogenase Activity Assay Kit	Gensource Co., Ltd.	Cat#LDH0360
LDH assay kit	Sigma-Aldrich	Cat#MAK066
Cell counting kit-8	Solarbio	Cat# 1210-500
CardioTACS in situ apoptosis detection kit	Roche	Cat#11684795910
Stratagene's QuikChange II site-directed mutagenesis kit	Stratagene	Cat#210518
**Instrument and software**		
VEVO-2100 System	VisualSonics	RRID:SCR_015816
GraphPad Prism version 8.0.1	GraphPad Software	RRID:SCR_002798
SPSS 24.0 software package	SPSS	RRID:SCR_019096
Amicon Ultra-4 10K Centrifugal Filter Devices	Millipore	Cat#UFC801096
Accu-Check blood glucose meter	Roche	N/A
**Drug and Reagent**		
saponin	Sigma-Aldrich	Cat#S4521
Alcian blue	Sigma-Aldrich	Cat#A3157
Brilliant Green	Amresco	Cat#5141-20-8
2,3,5-triphenyl-tetrazolium chloride	Sigma-Aldrich	Cat#T8877
LY2090314	MedChemExpress	Cat#HY-16294
CHIR99021	MedChemExpress	Cat#HY-10182
Glucose-free DMEM	ThermoFisher	Cat#11966
1640	Corning	Cat# 10-040-CVR
Lipofectamine 2000	Invitrogen	Cat#11668027
Laemmli buffer	BIO-RAD	Cat#1610737
Fluorescent mounting medium with DAPI	ZSGB-BIO	Cat# ZLI-9557
High glucose DMEM	Solarbio	Cat# 11995
Protein A Sepharose™ 4 Fast Flow	GE Healthcare	Cat#17-5280-02
EZview Red Anti-Myc Affinity Gel	Sigma-Aldrich	Cat#CFAD-E6654
Immobilon-P membrane	Millipore	Cat#IPVH00010

ARRIVE GUIDELINES

The ARRIVE guidelines (https://arriveguidelines.org/) are a checklist of recommendations to improve the reporting of research involving animals. Key elements of the study design should be included below to better enable readers to scrutinize the research adequately, evaluate its methodological rigor, and reproduce the methods or findings.

Study Design

**Groups**	**Sex**	**Age**	**Number (prior to experiment)**	**Number (after termination)**	**Littermates (Yes/No)**	**Other description**
***db/db* mice**						
**Fig6A**						
Group 1 (HSA)	M	8 weeks	12	10	No	
Group 2 (rhMG53-WT)	M	8 weeks	12	11	No	
Group 3 (rhMG53-S255A)	M	8 weeks	12	10	No	
**Fig6D**						
Group 4 (HSA)	M	12 weeks	11	9	No	
Group 5 (rhMG53-WT)	M	12 weeks	11	9	No	
Group 6 (rhMG53-S255A)	M	12 weeks	11	11	No	
**Fig6G**						
Group 7 (HSA)	F	12 weeks	11	11	No	
Group 8 (rhMG53-WT)	F	12 weeks	13	13	No	
Group 9 (rhMG53-S255A)	F	12 weeks	11	11	No	
**post-ischemia (Fig6K)**						
Group 10 (HSA)	M	12 weeks	12	12	No	
Group 11 (rhMG53-WT)	M	12 weeks	12	12	No	
Group 12 (rhMG53-S255A)	M	12 weeks	12	12	No	
**tPA-MG53 (Fig7C)**						
Group 13 (null)	M	12 weeks	7	7	No	
Group 14 (tPA-MG53-WT)	M	12 weeks	9	9	No	
Group 15 (tPA-MG53-S255A)	M	12 weeks	9	9	No	
**Fibrosis (Fig7J)**						
Group 16 (HSA)	M	8 weeks	10	6	No	
Group 17 (rhMG53-WT)	M	8 weeks	11	6	No	
Group 18 (rhMG53-S255A)	M	8 weeks	10	6	No	
**$MG53^{-/-}$ mice (Fig6I)**						
Group 19 (HSA)	M	6-8 weeks	10	8	No	
Group 20 (rhMG53-WT)	M	6-8 weeks	10	7	No	
Group 21 (rhMG53-S255A)	M	6-8 weeks	10	9	No	
**$MG53\text{-}S255A^{ki/+}$ (Fig7D)**						
Group 22 (WT+control)	M	7-8 weeks	5	5	Yes	
Group 23 (WT+STZ)	M	7-8 weeks	5	5	Yes	
Group 24 ($S255A^{ki/+}$ +STZ)	M	7-8 weeks	5	5	Yes	
**Rat (FigS10B)**						

DOI [to be added]

Group 25 (HSA)	M	8 weeks	18	14	No	
Group 26 (rhMG53-WT)	M	8 weeks	14	13	No	
Group 27 (rhMG53-S255A)	M	8 weeks	7	7	No	

Sample Size: Please explain how the sample size was decided Please provide details of any a *prior* sample size calculation, if done.

No statistical method was used to predetermine the sample size. The number of animals used in each experiment was determined based upon sample size commonly used in the field.

Inclusion Criteria

All of the animals were included except for those died right after the surgery.

Exclusion Criteria

The animals died from the surgery were excluded.

Randomization

All animals were randomly assigned to different experimental groups.

Blinding

Data acquisition and analysis were performed by investigators who were blinded to the group assignment.
