# Supplementary material for: Blocking MG53S255 Phosphorylation Protects Diabetic Heart From Ischemic Injury
Source: Circ Res. 2022 Nov 7;131(12):962–76. doi: 10.1161/CIRCRESAHA.122.321055 (PMC9770150; doi:10.1161/CIRCRESAHA.122.321055)
Supplement: Supplementary file 6 [file res-131-0962-s006.pdf]

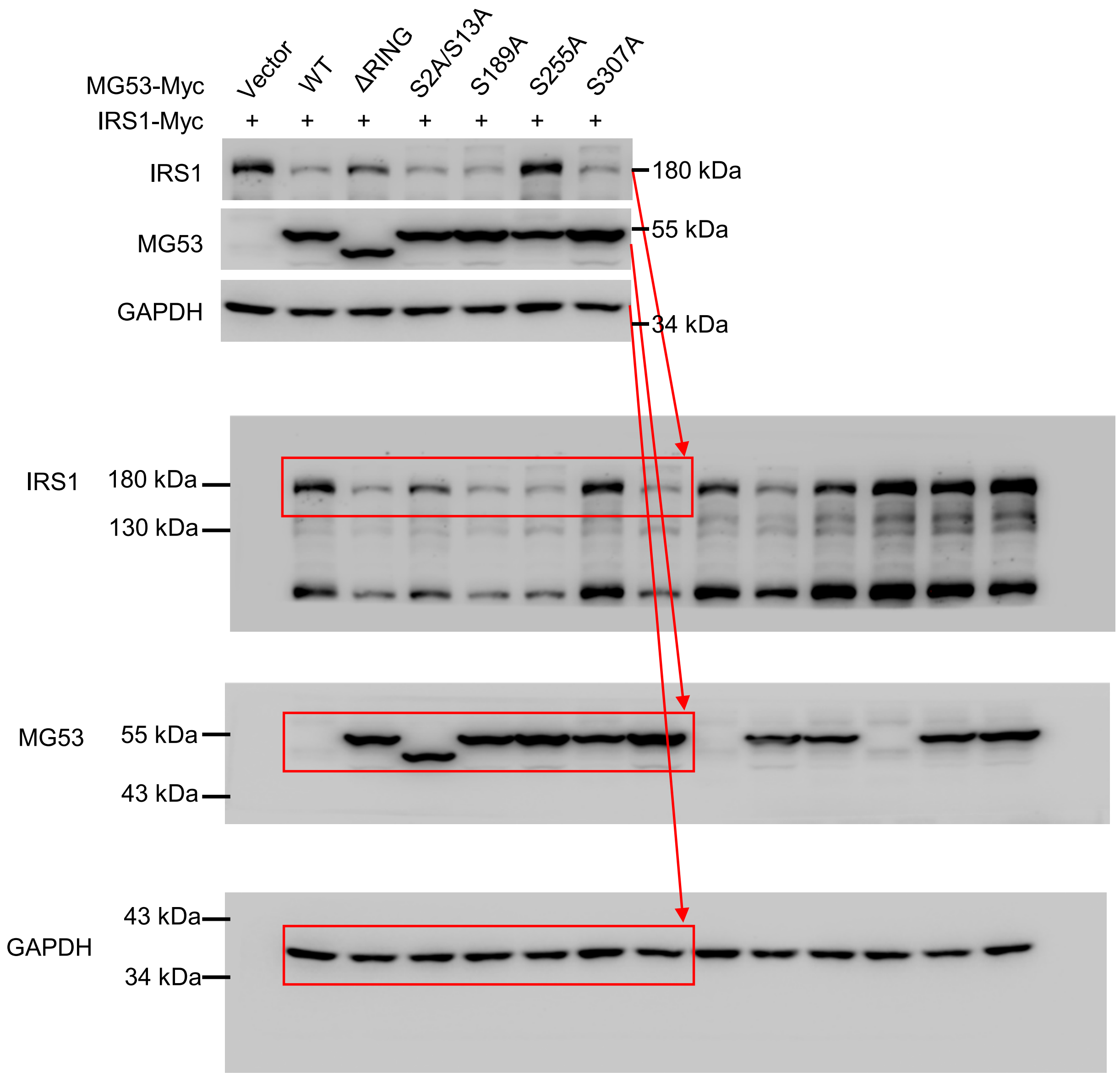

Full unedited gel for Figure 1A

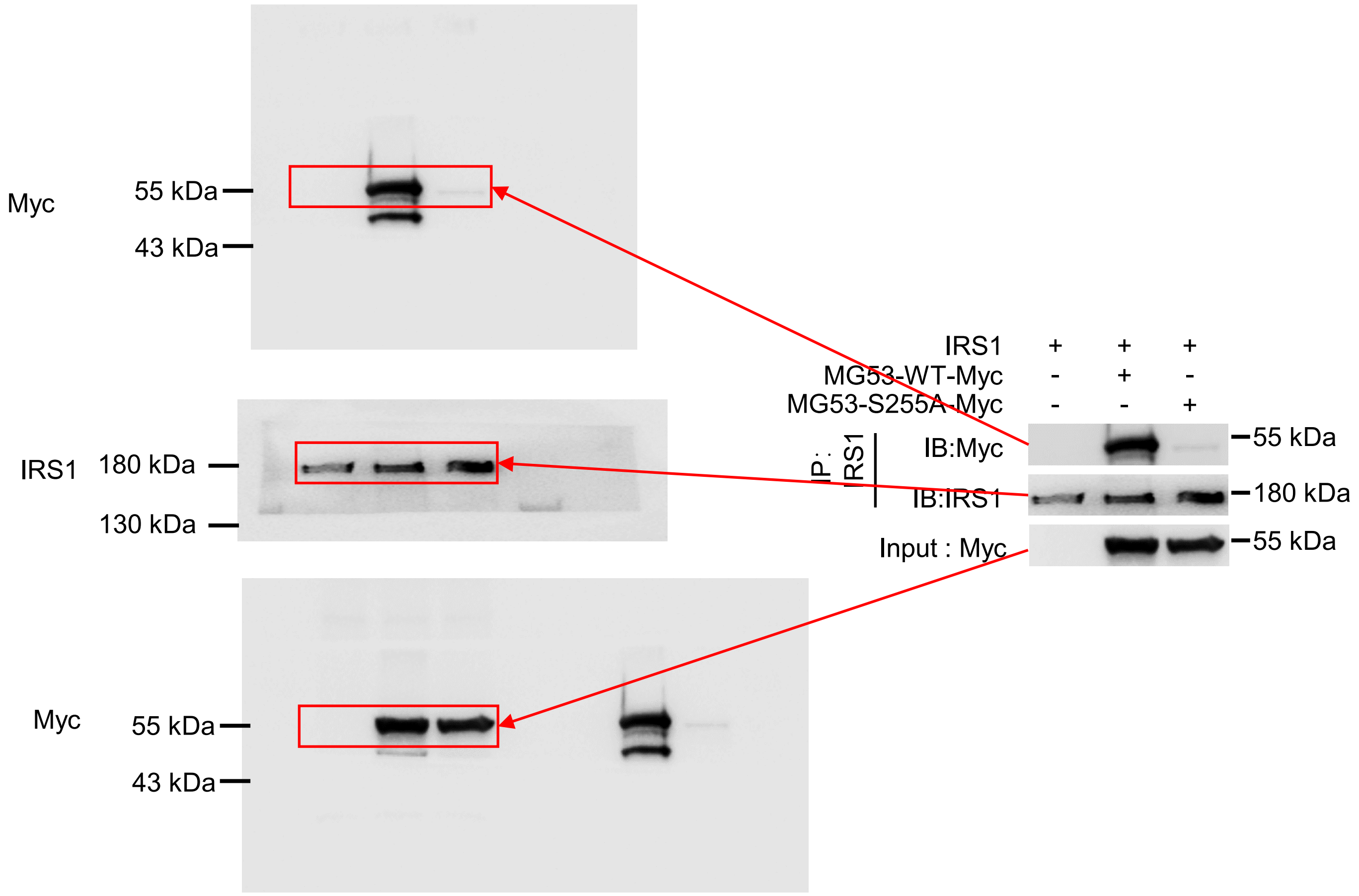

Full unedited gel for Figure 1B

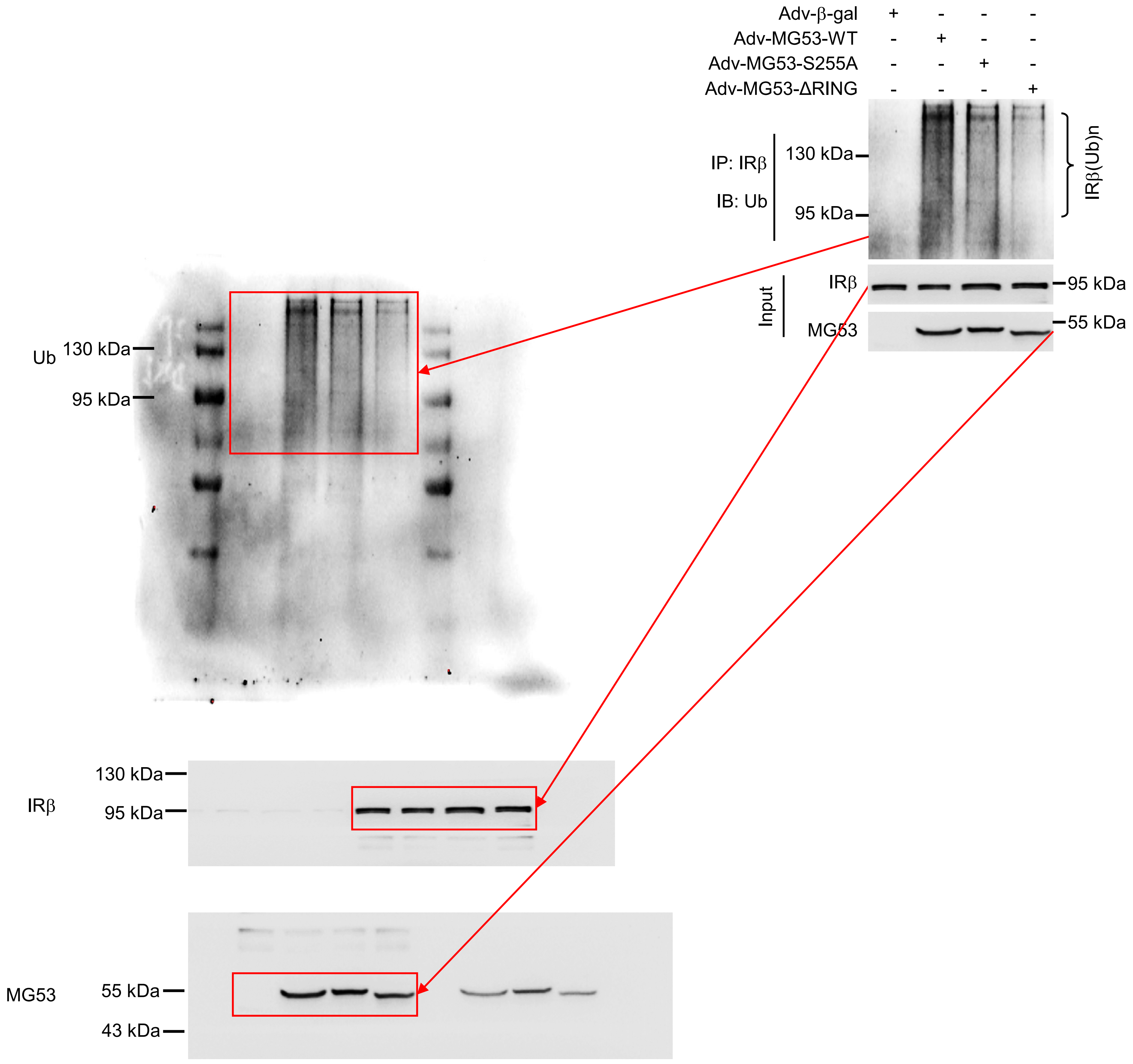

Full unedited gel for Figure 1D

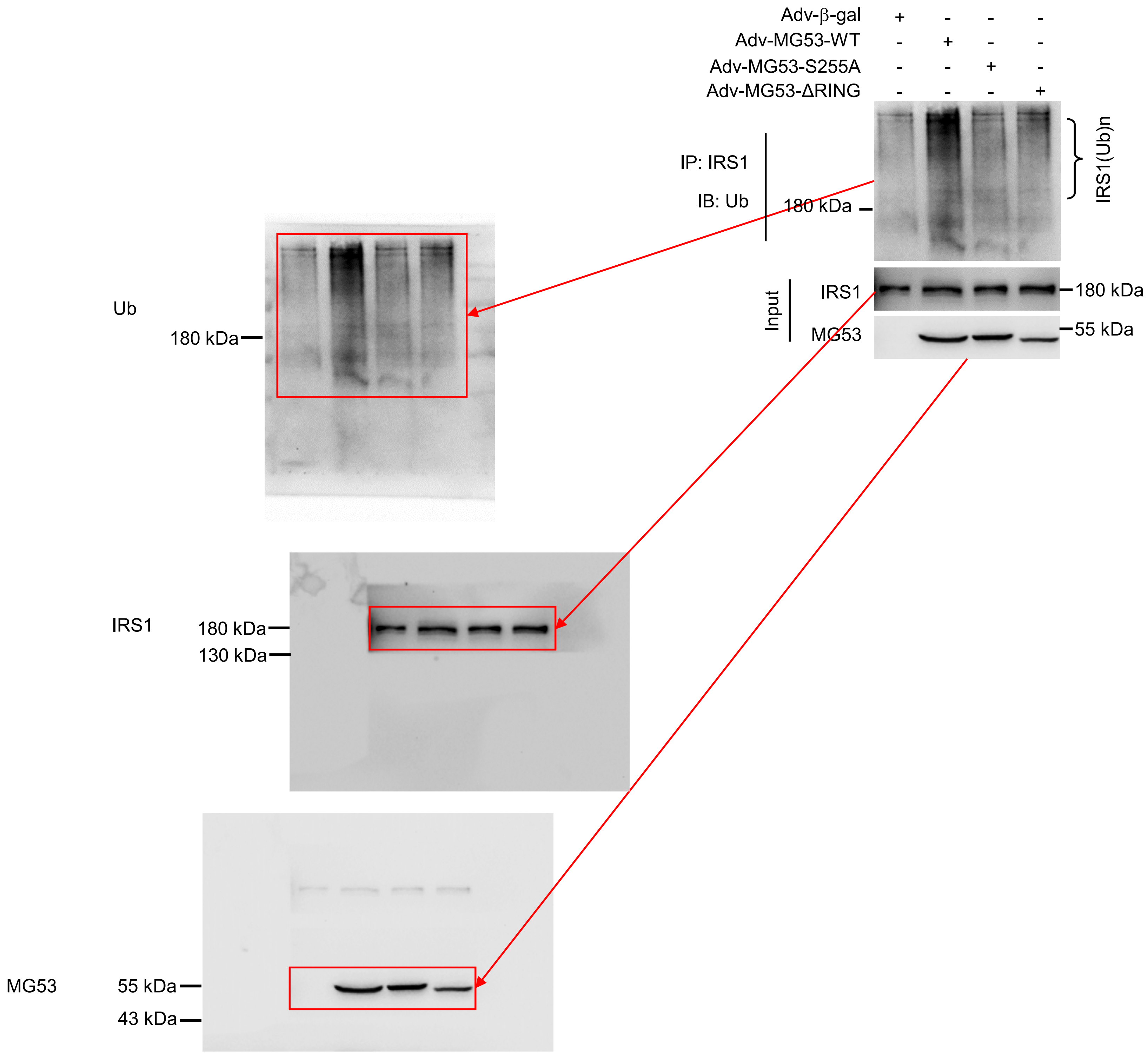

Full unedited gel for Figure 1E

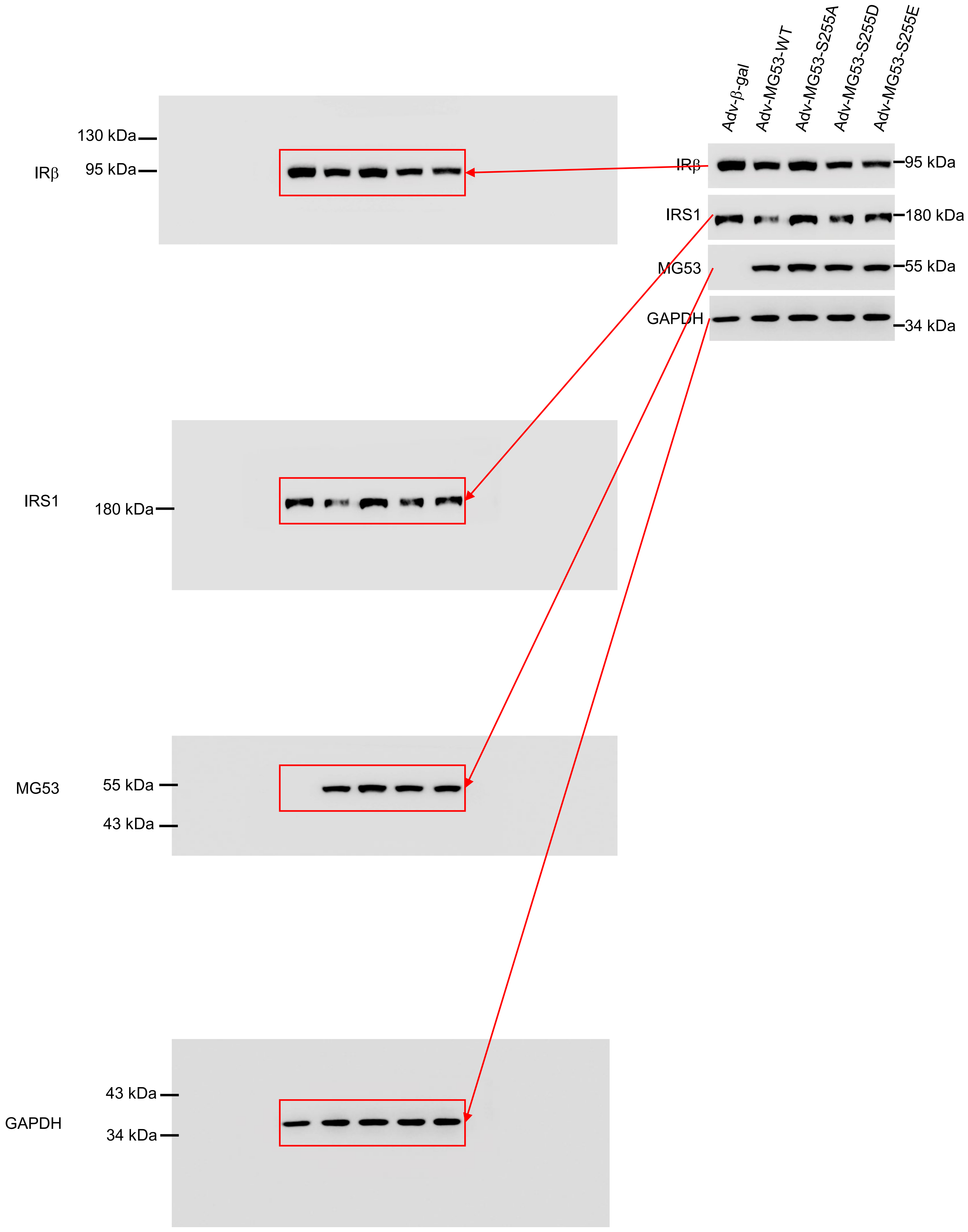

Full unedited gel for Figure 1F

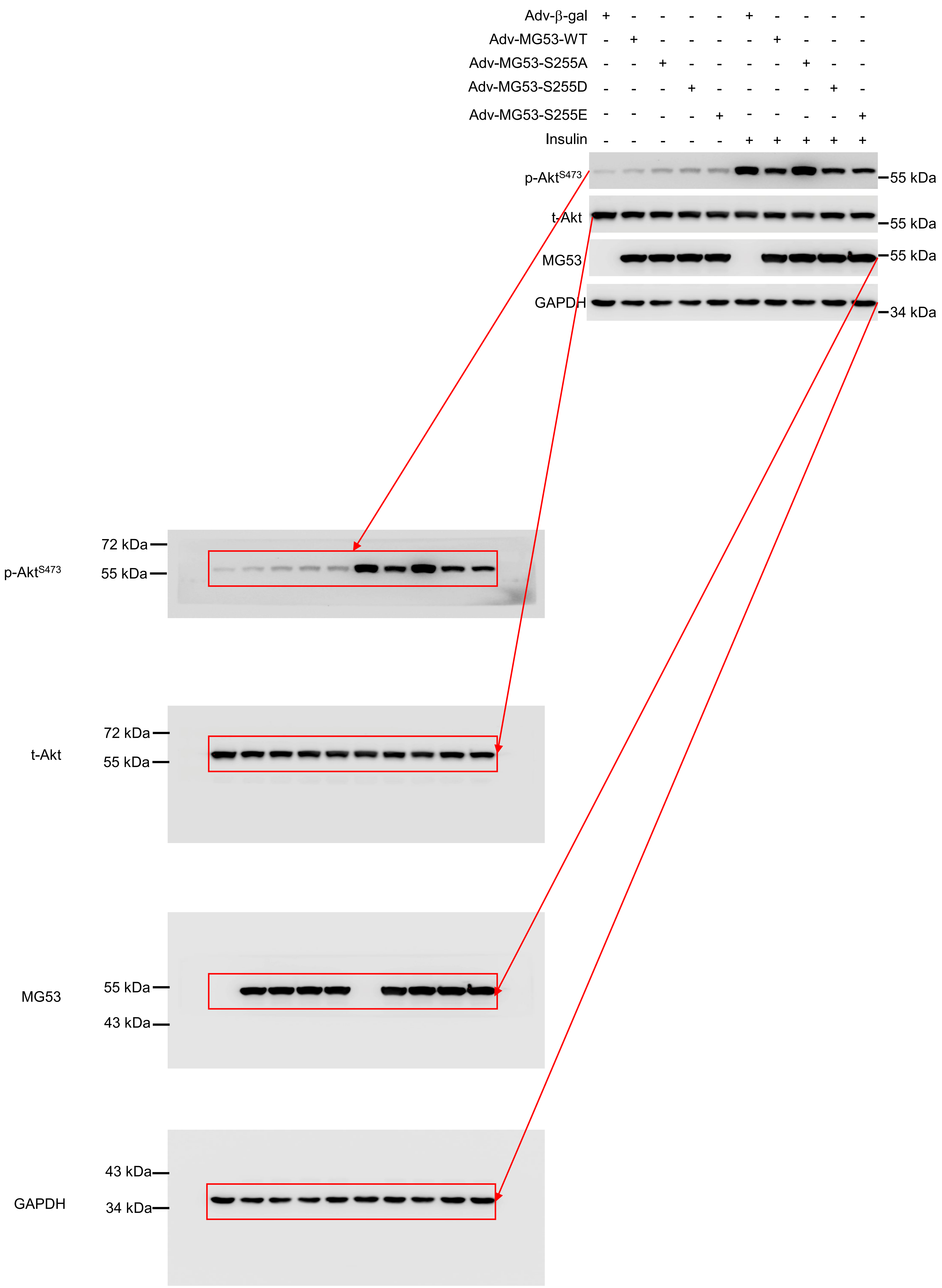

Full unedited gel for Figure 1G

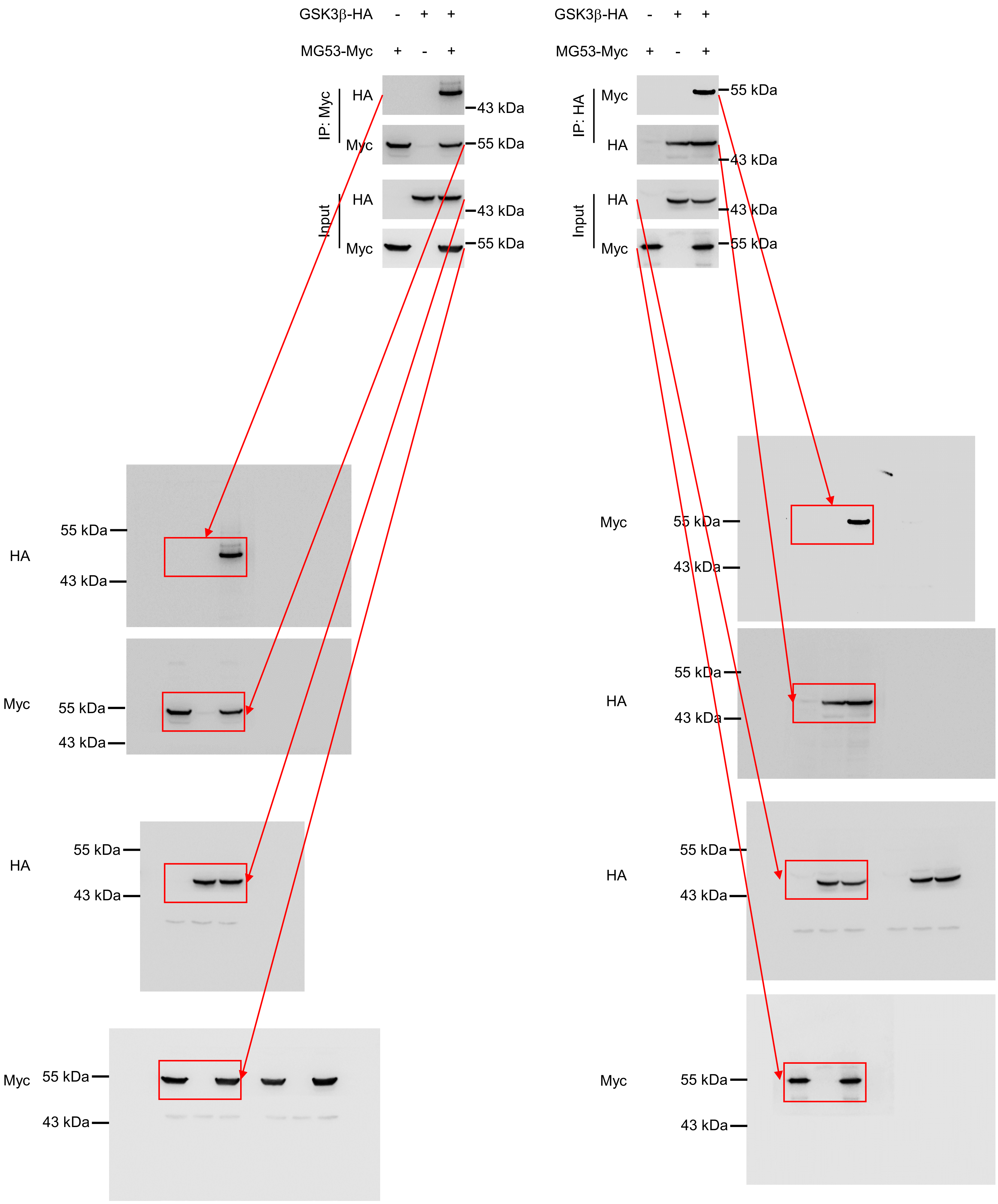

Full unedited gel for Figure 2A

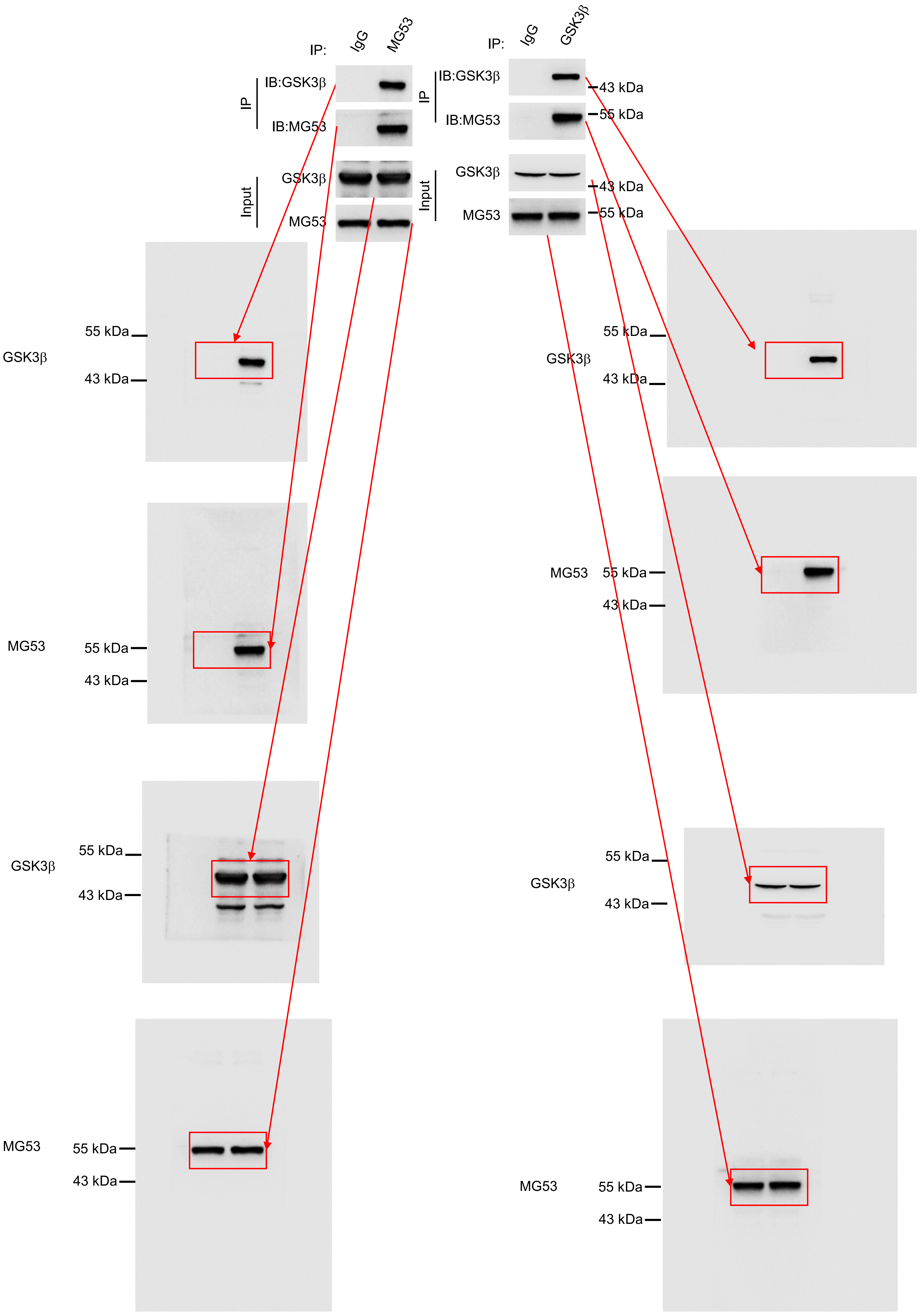

Full unedited gel for Figure 2B

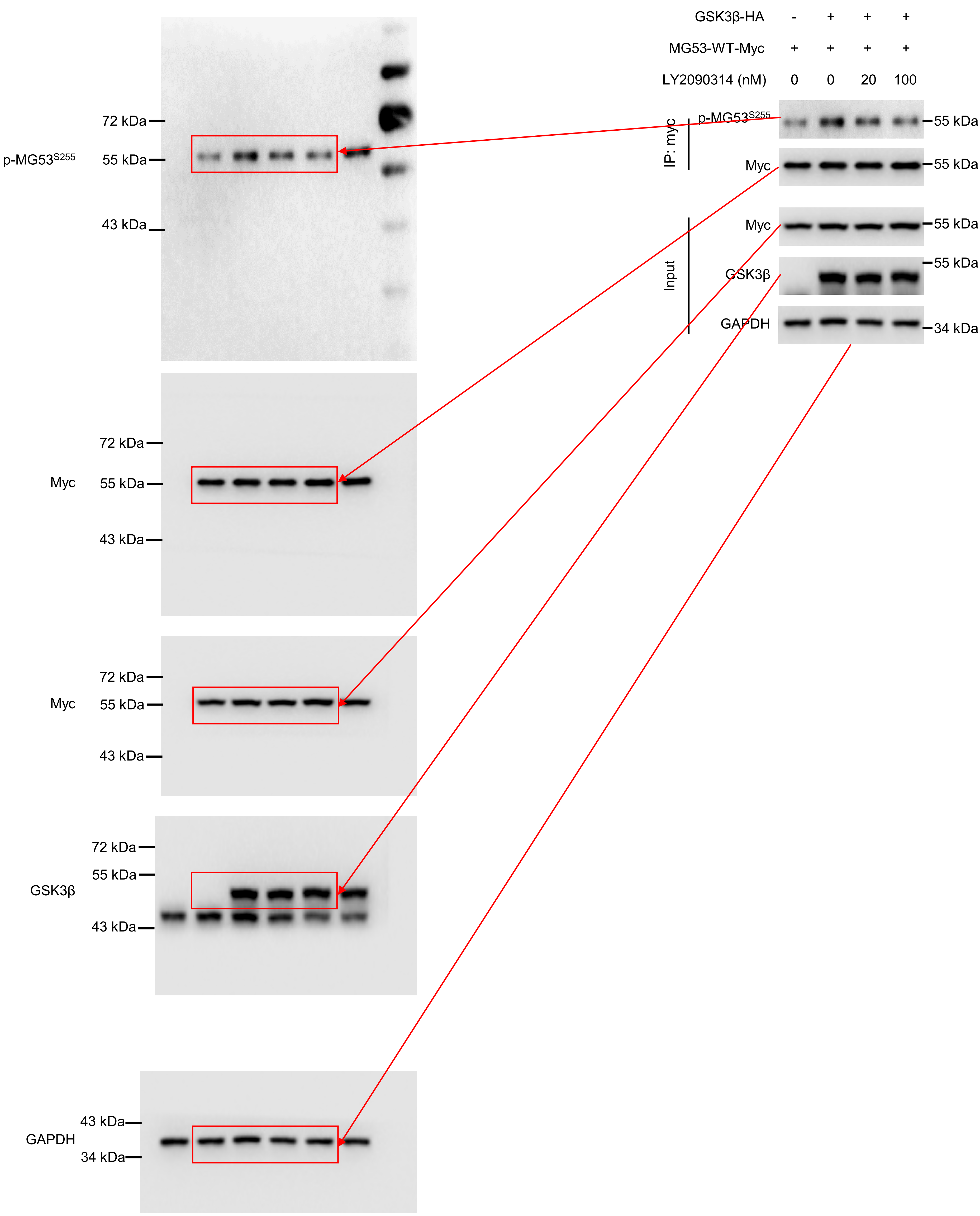

Full unedited gel for Figure 2C

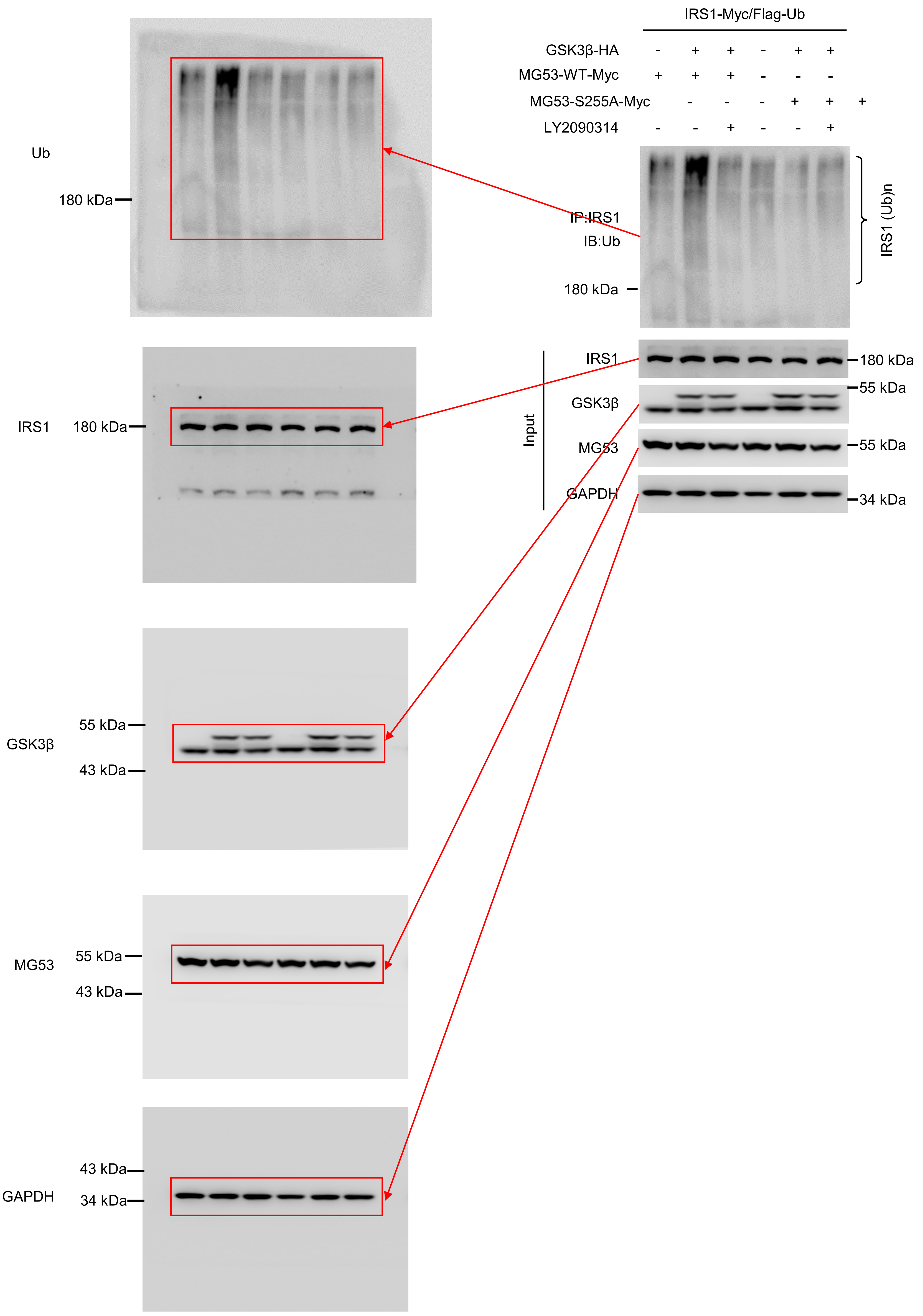

Full unedited gel for Figure 2D

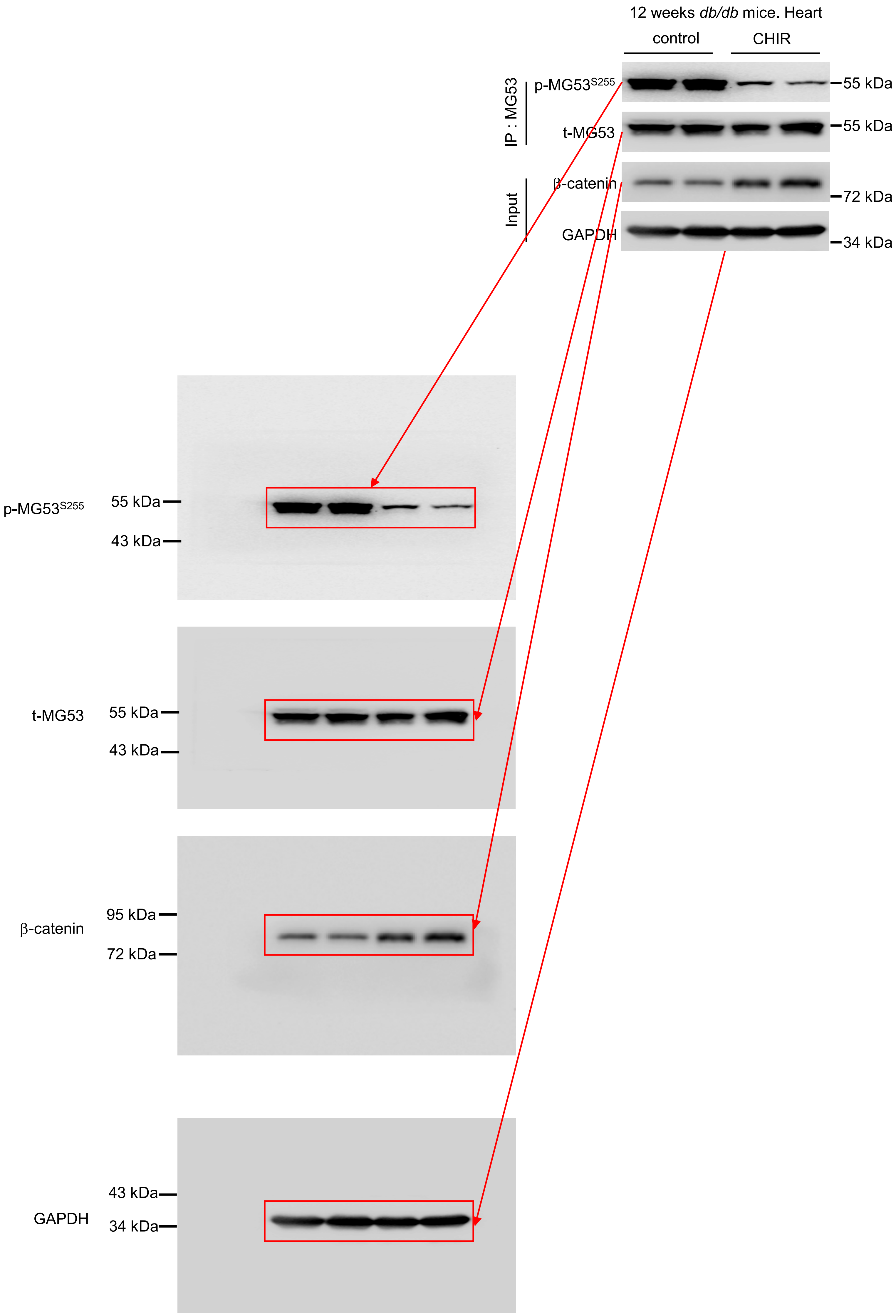

Full unedited gel for Figure 2E

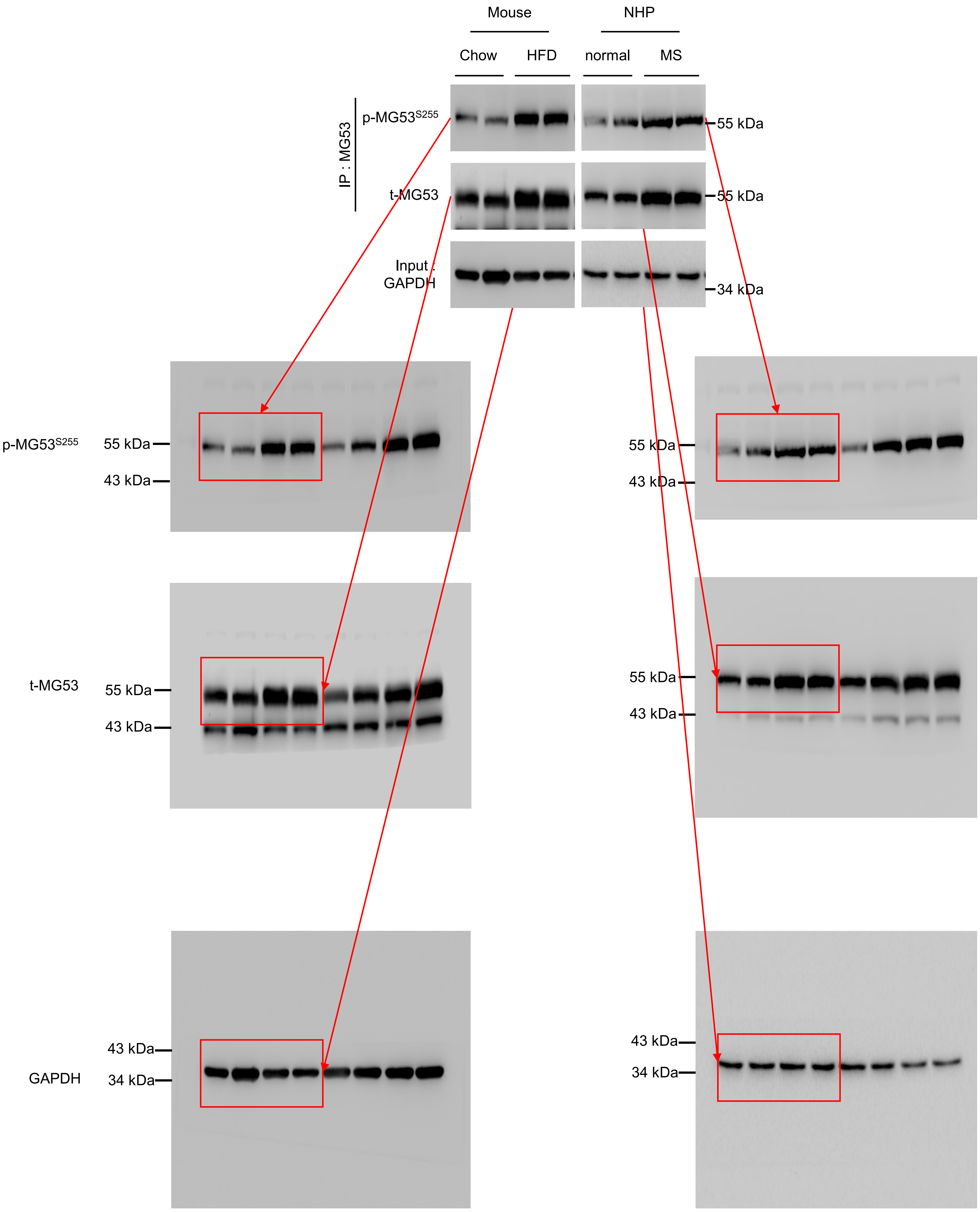

Full unedited gel for Figure 3A

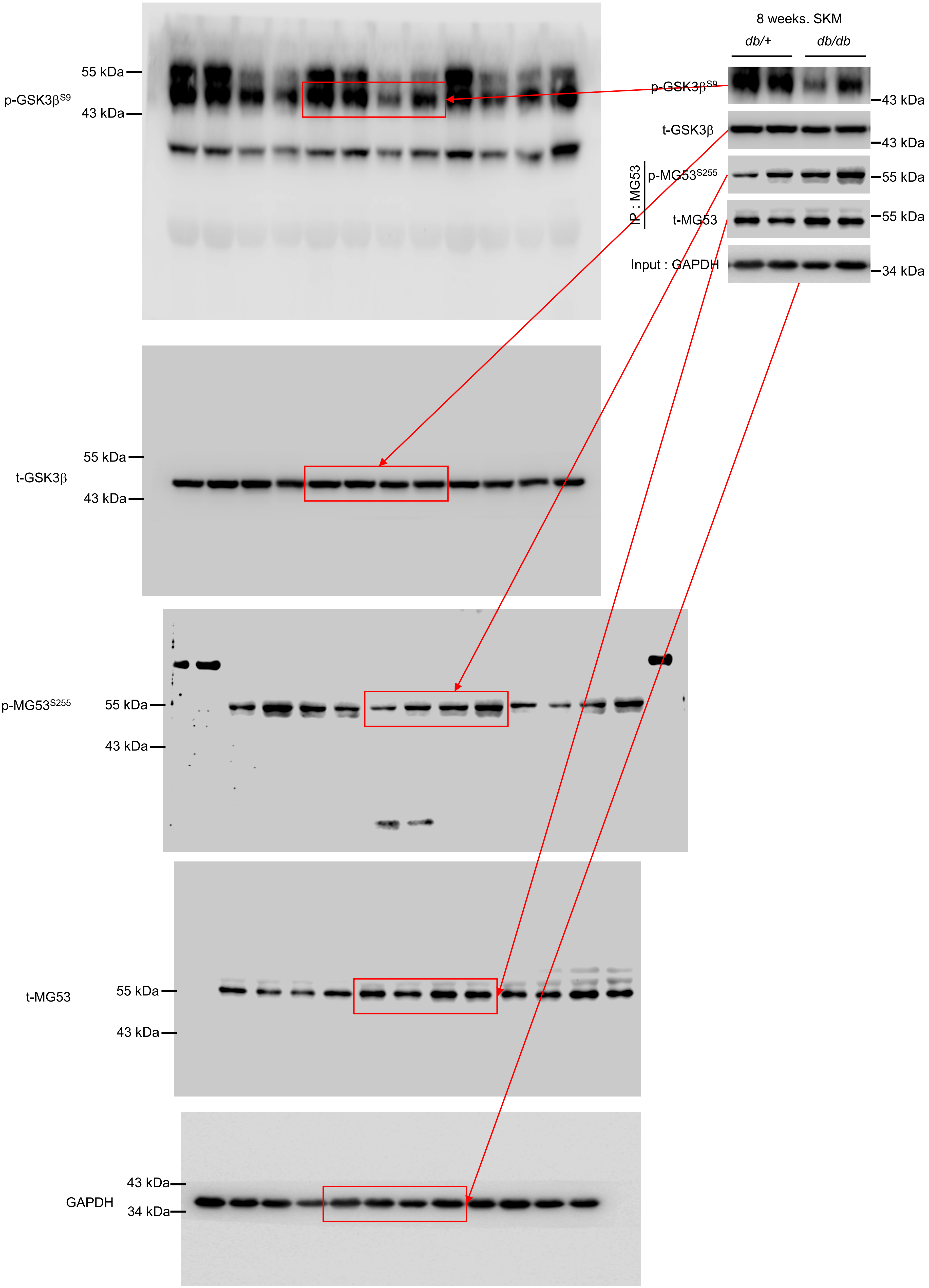

Full unedited gel for Figure 3B

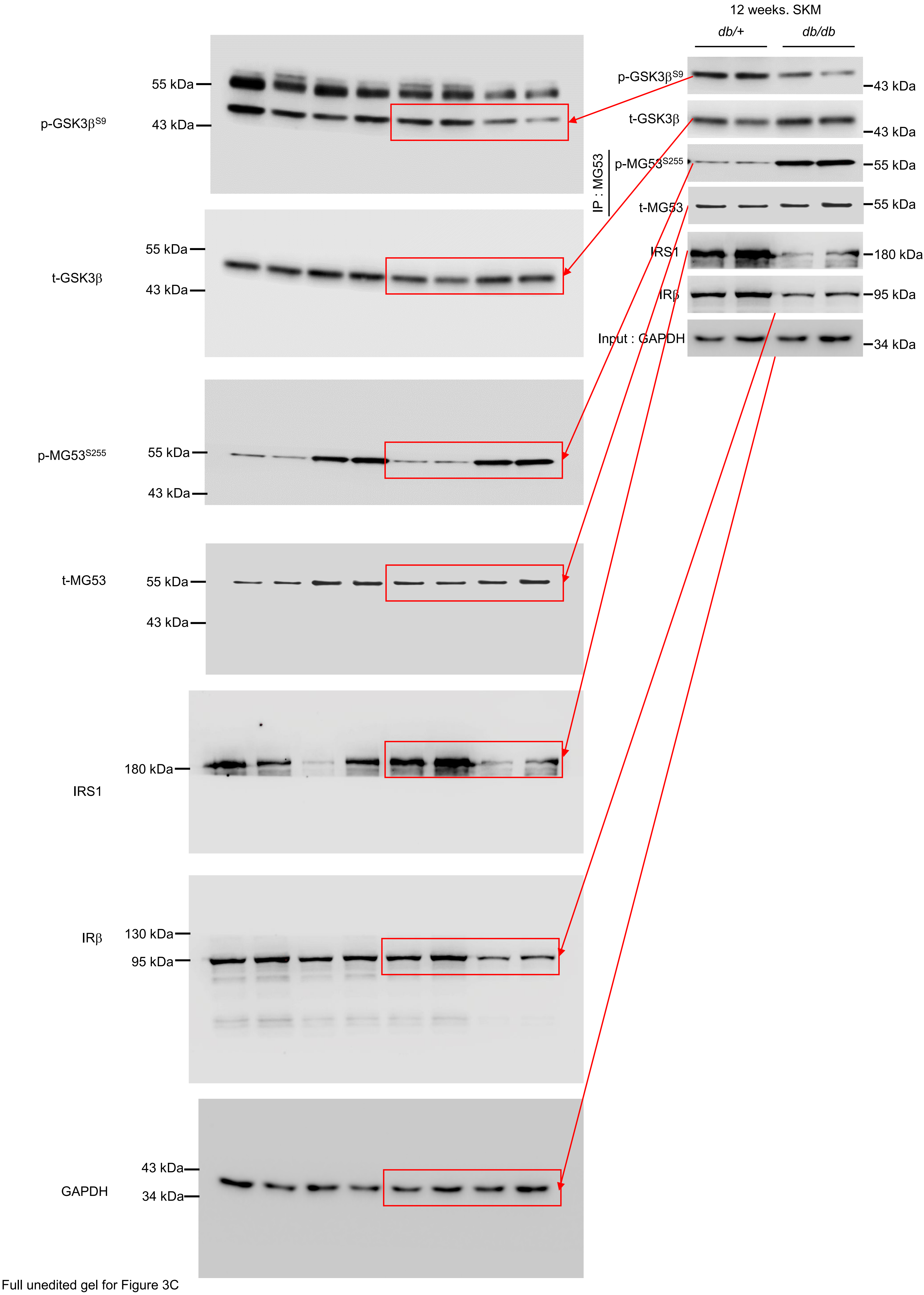

Full unedited gel for Figure 3C

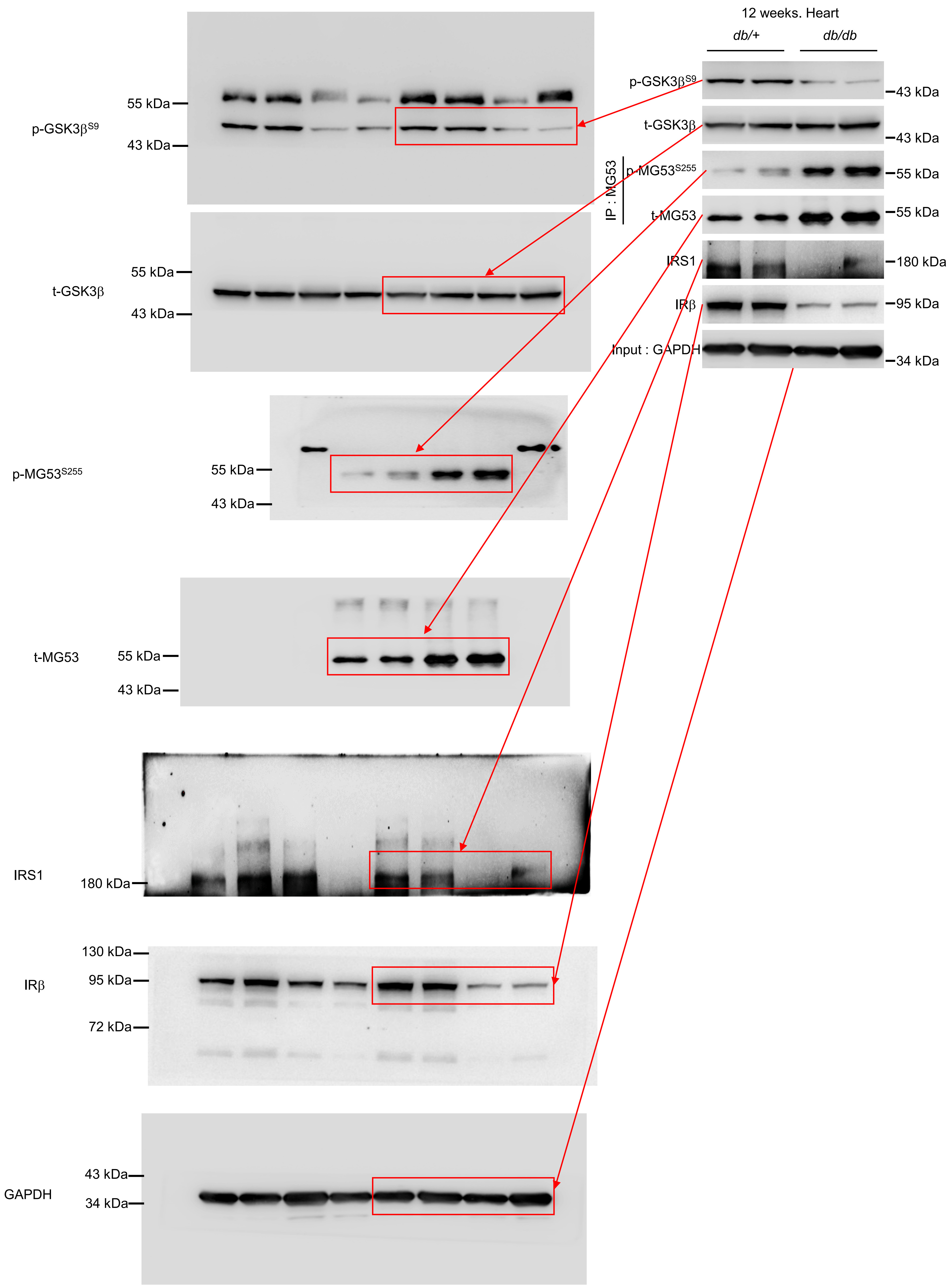

Full unedited gel for Figure 3D

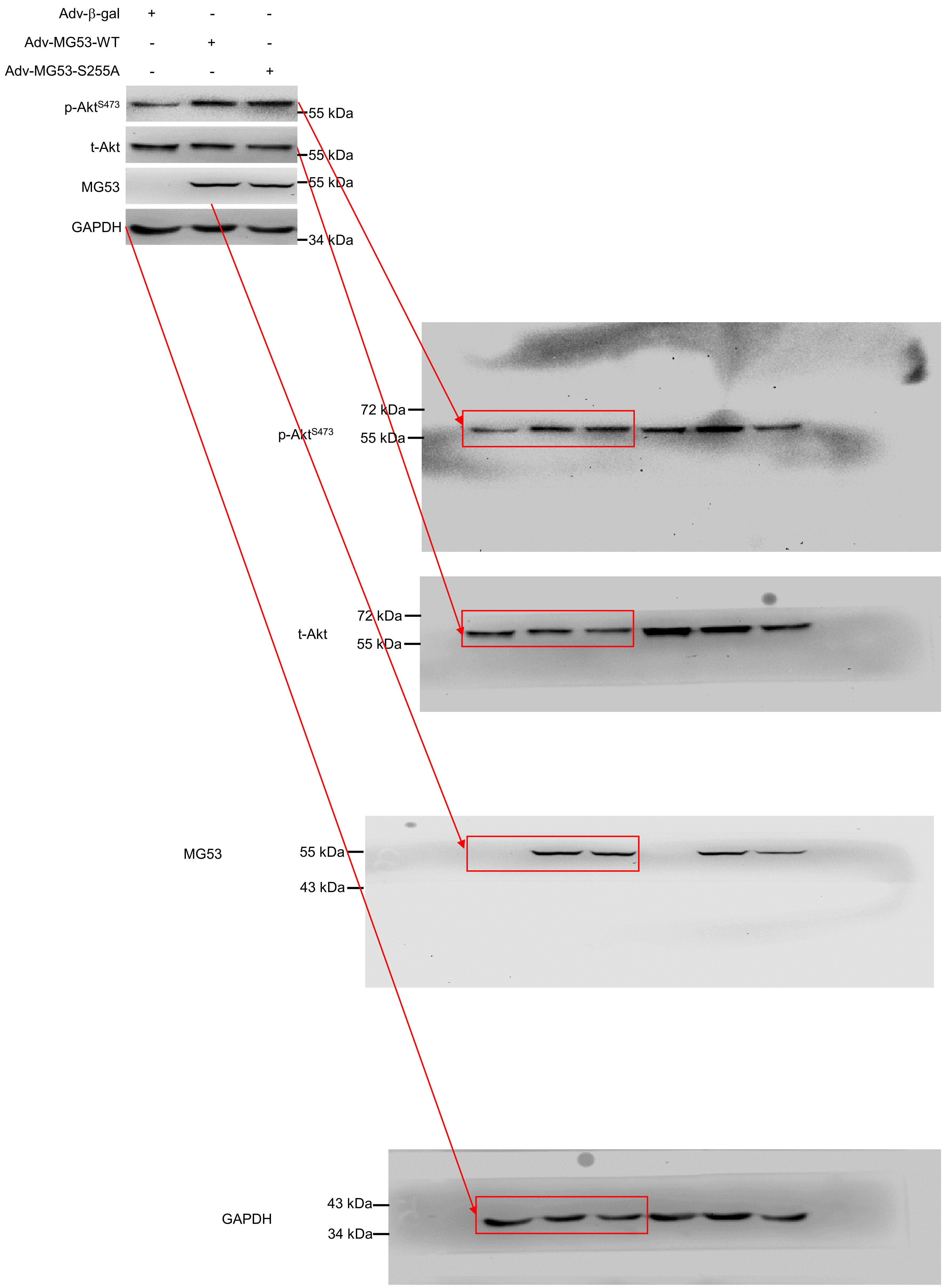

Full unedited gel for Figure 4C

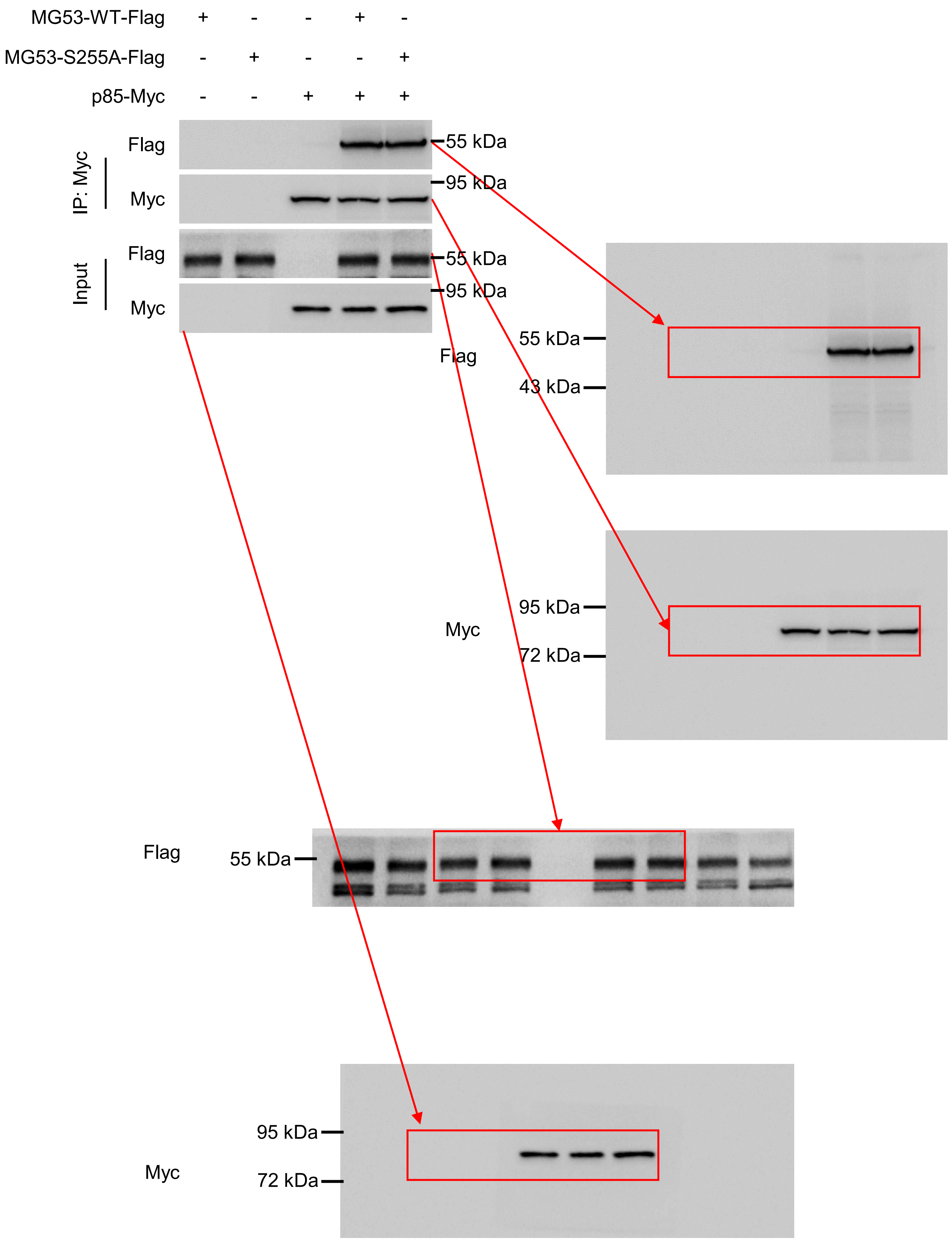

Full unedited gel for Figure 4D

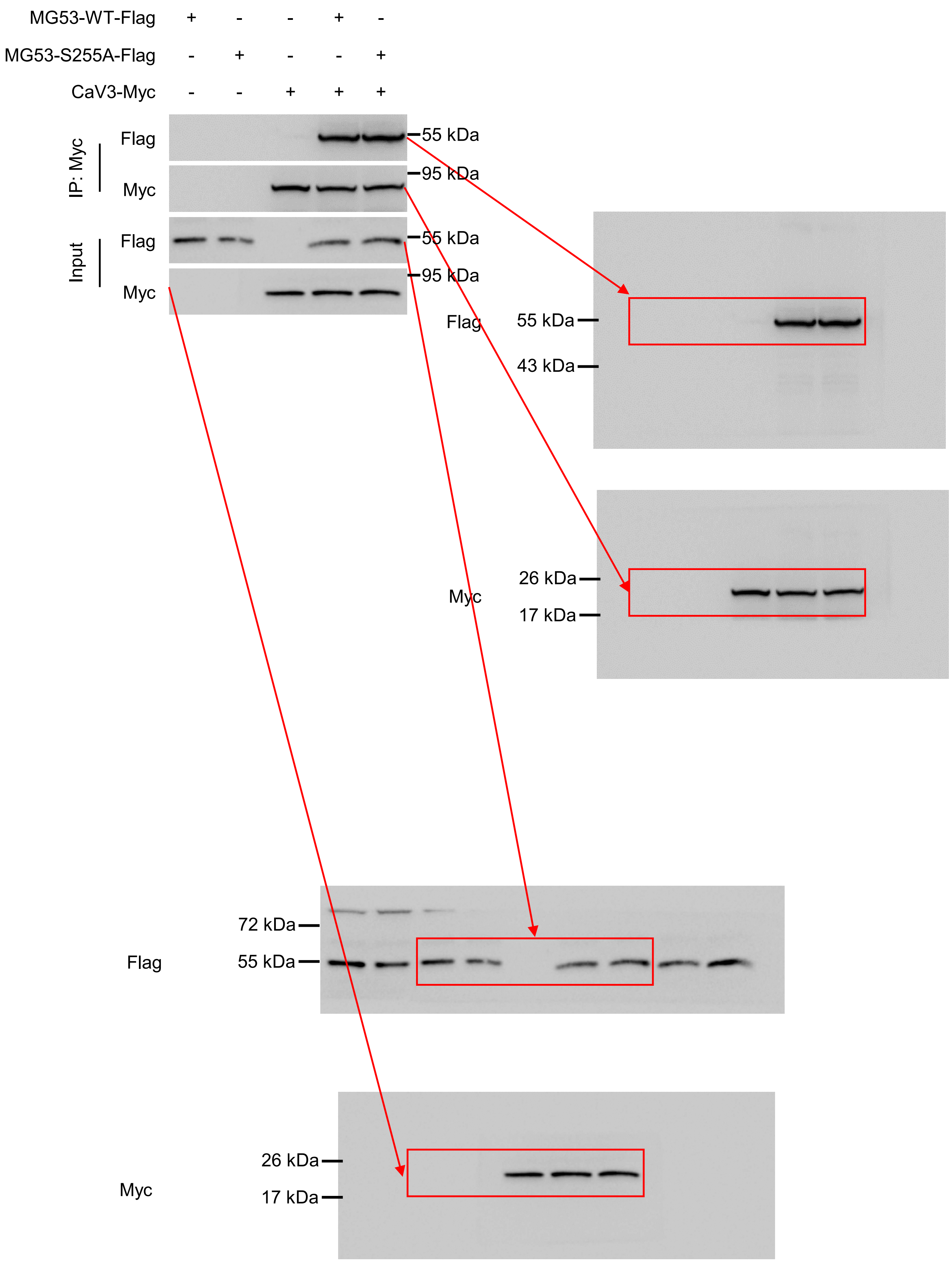

Full unedited gel for Figure 4D continued

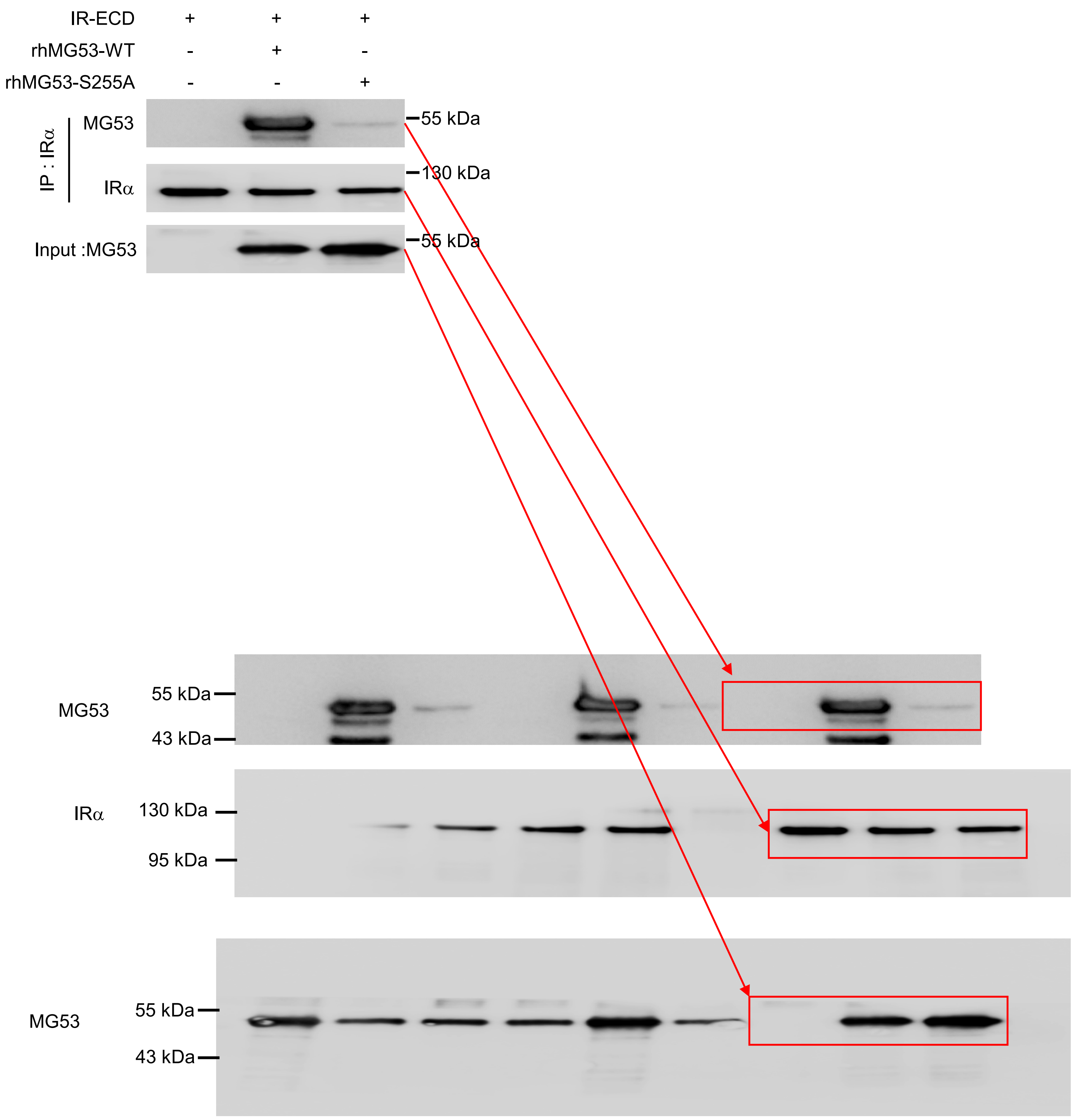

Full unedited gel for Figure 5A

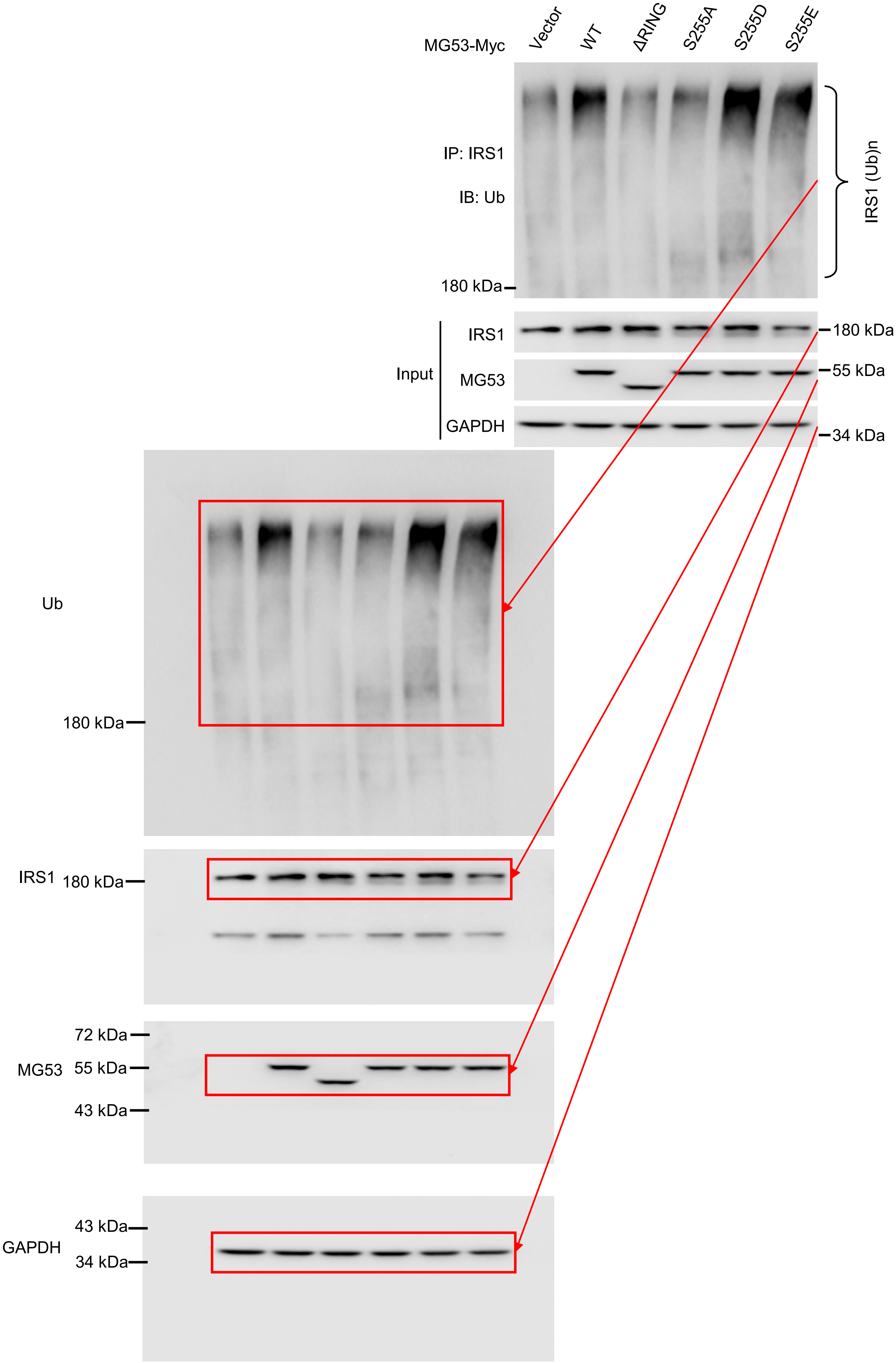

Full unedited gel for Figure S2A

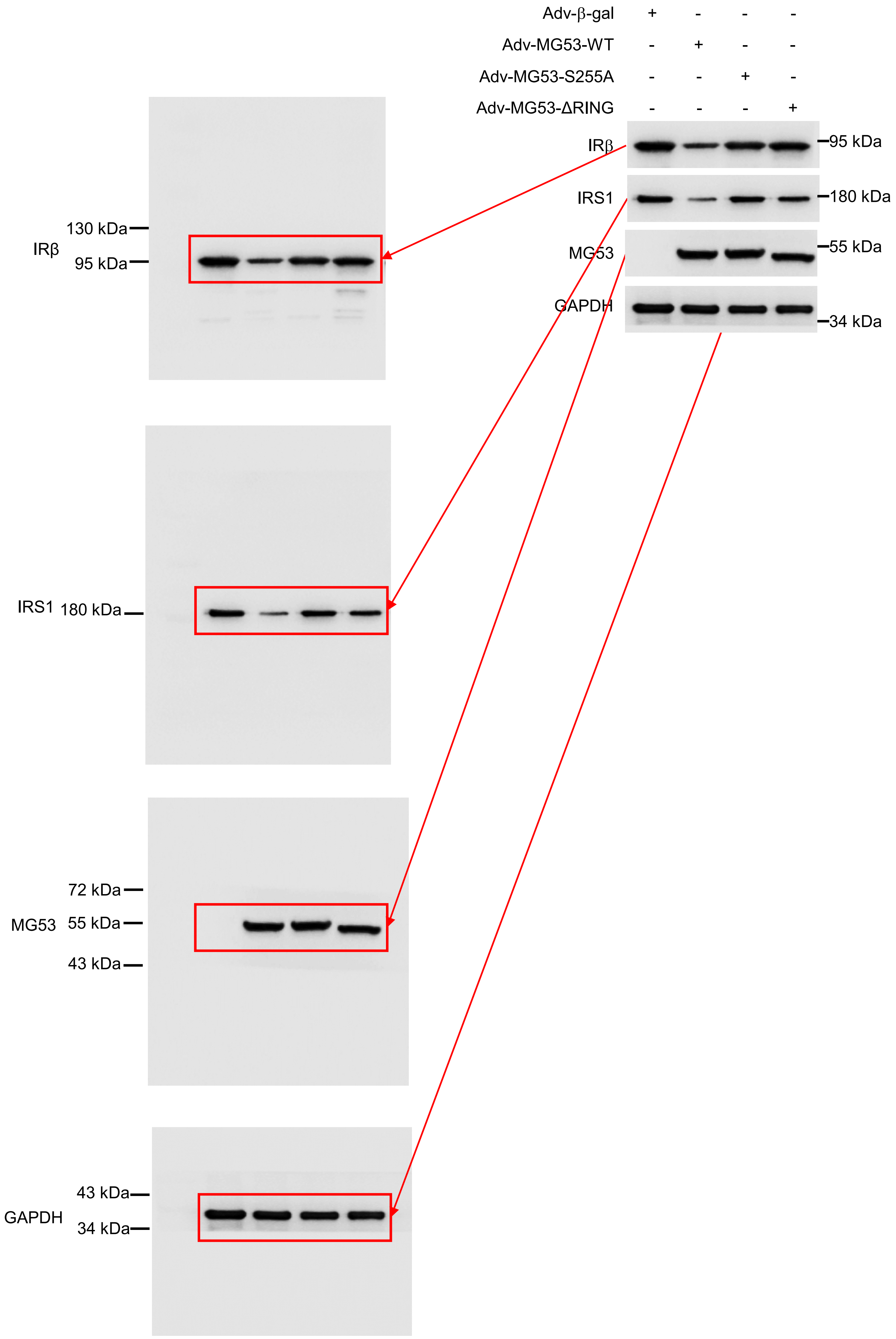

Full unedited gel for Figure S2C

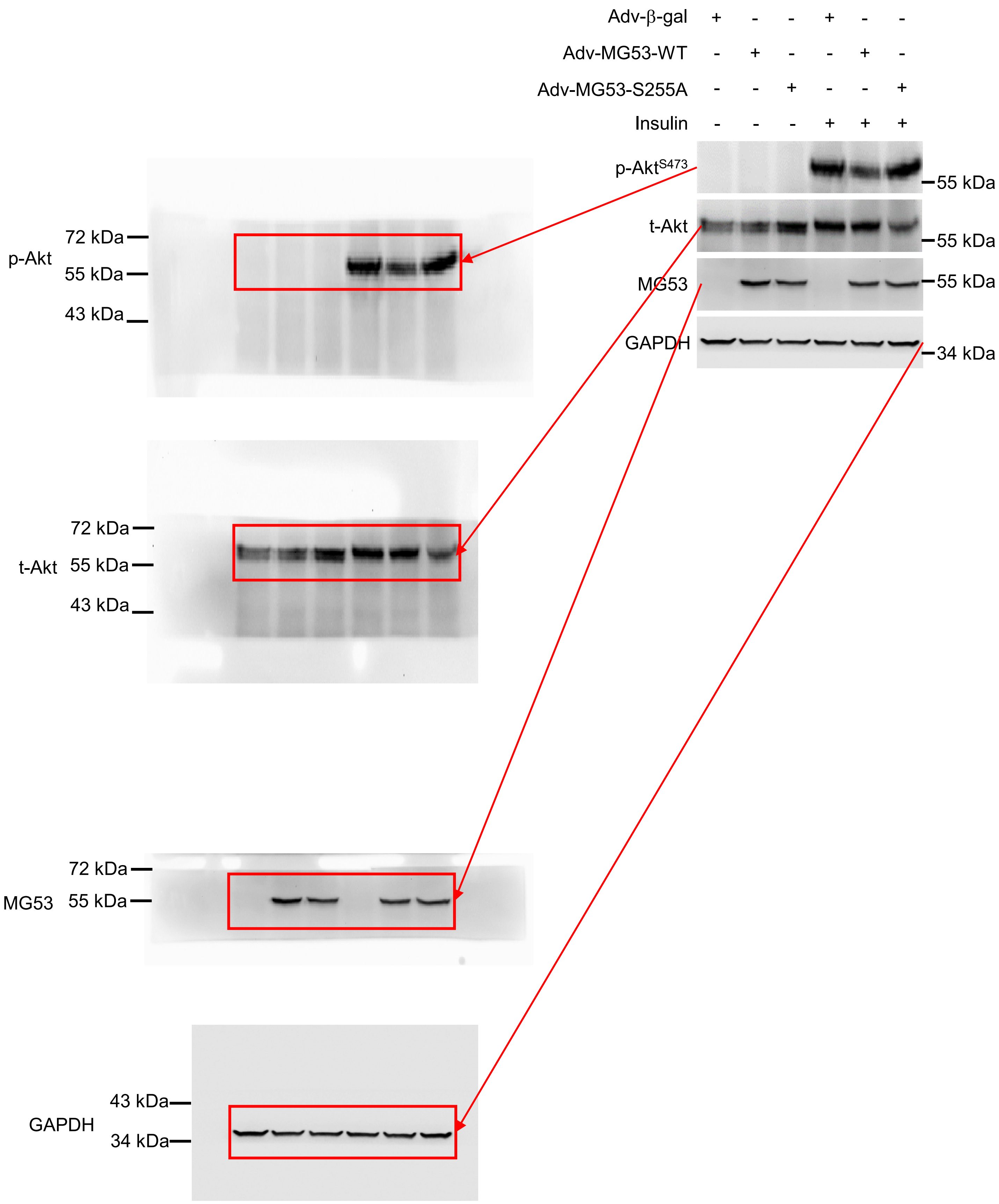

Full unedited gel for Figure S2D

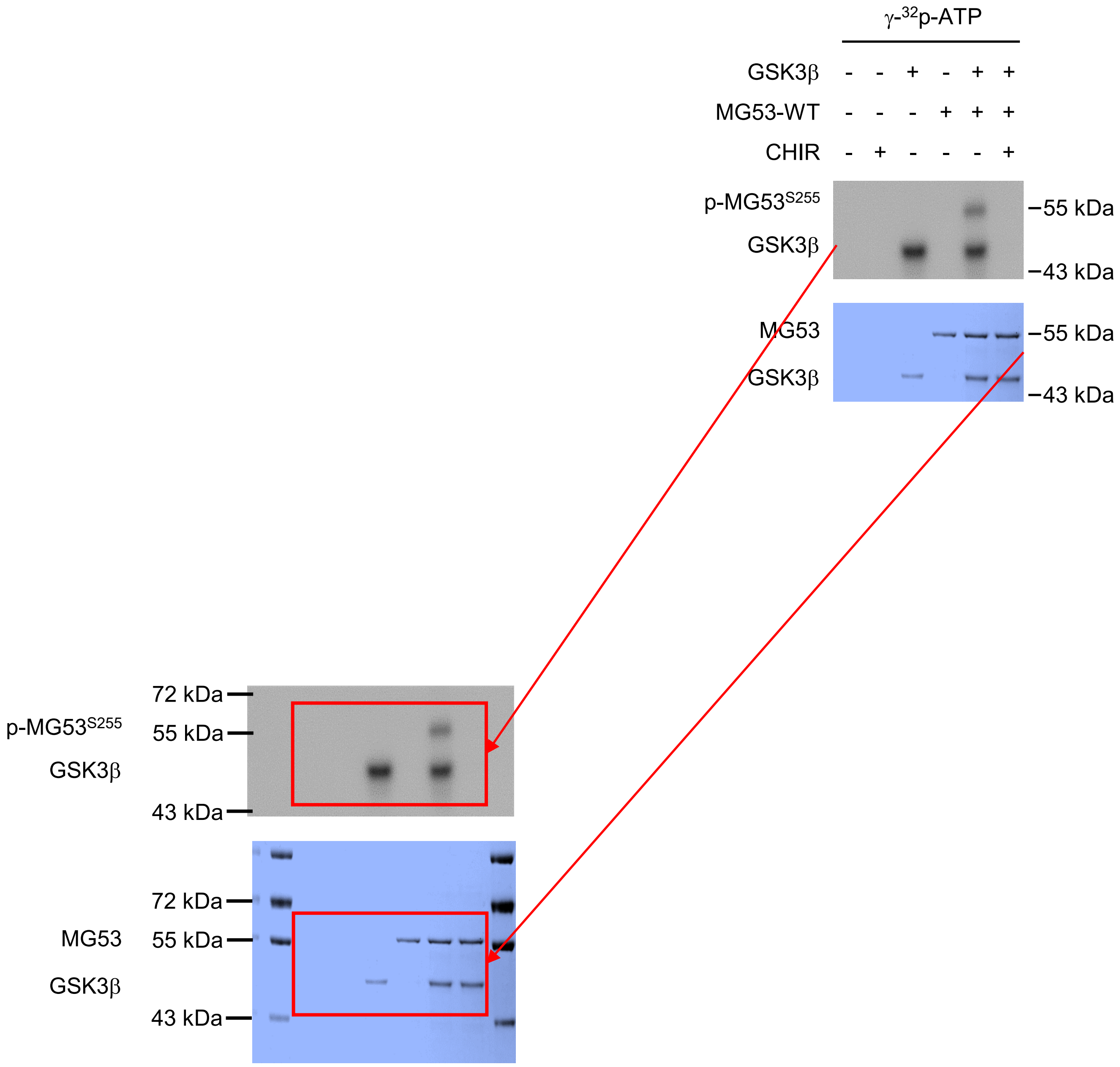

Full unedited gel for Figure S4A

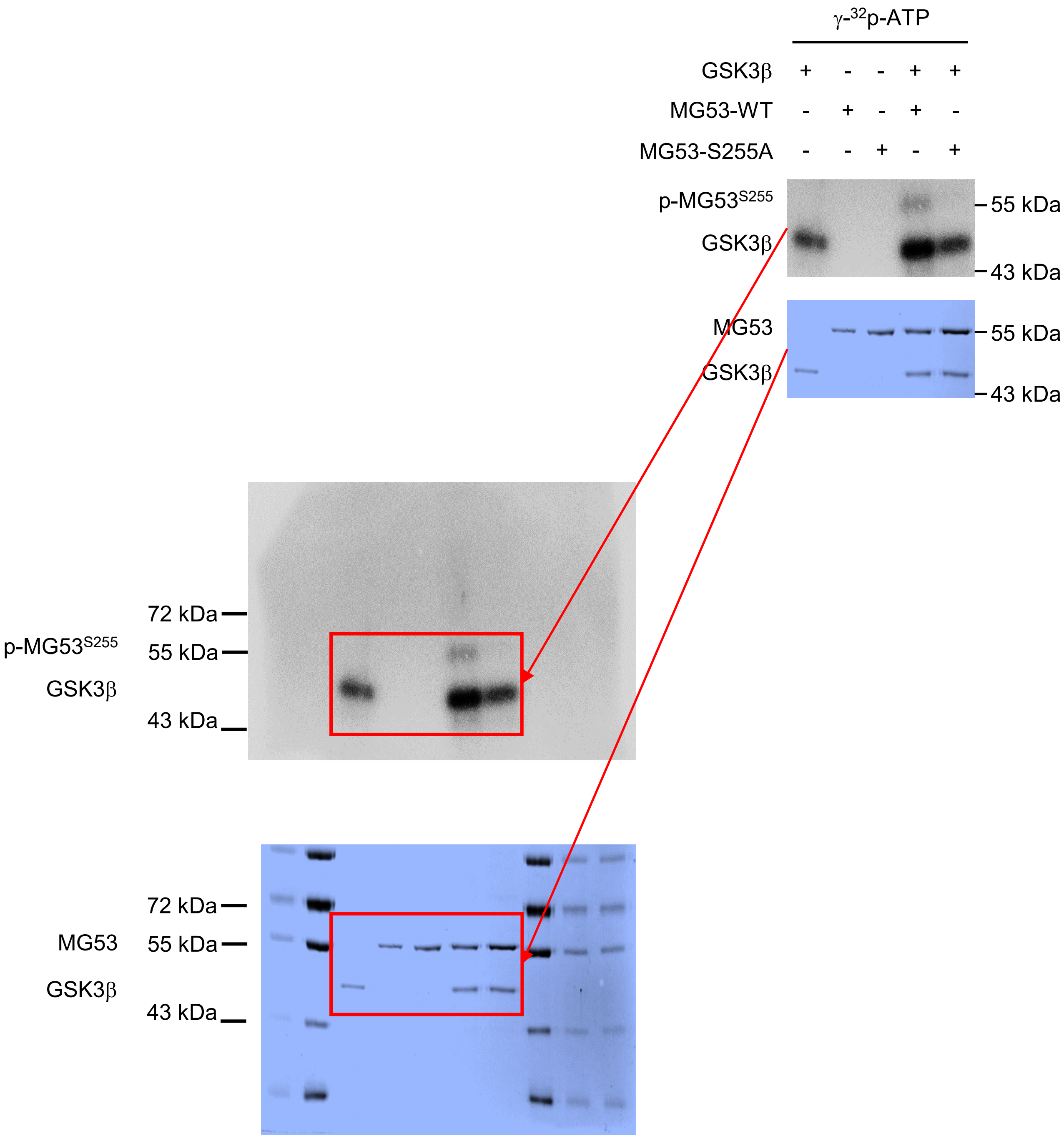

Full unedited gel for Figure S4B

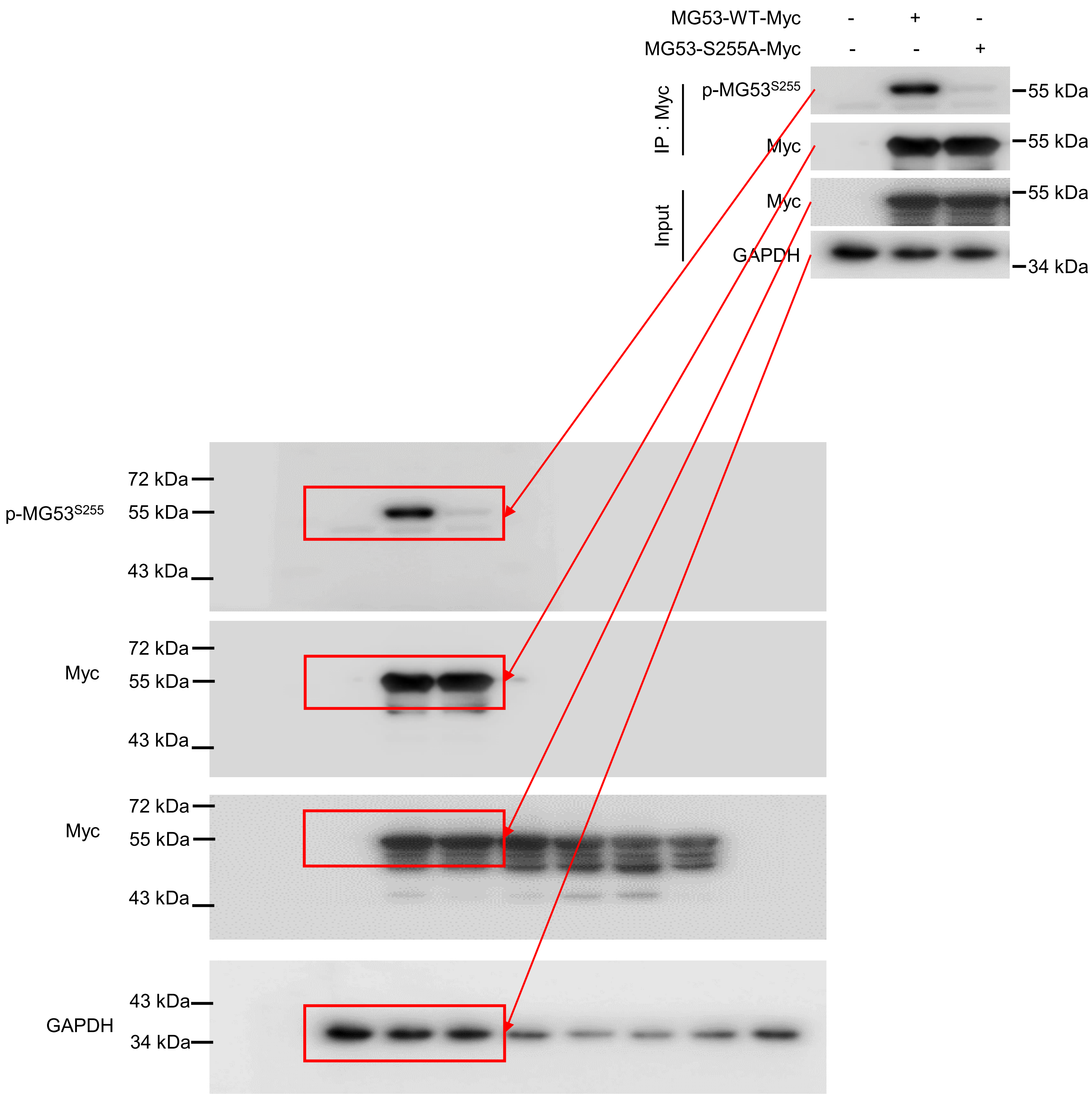

Full unedited gel for Figure S5A

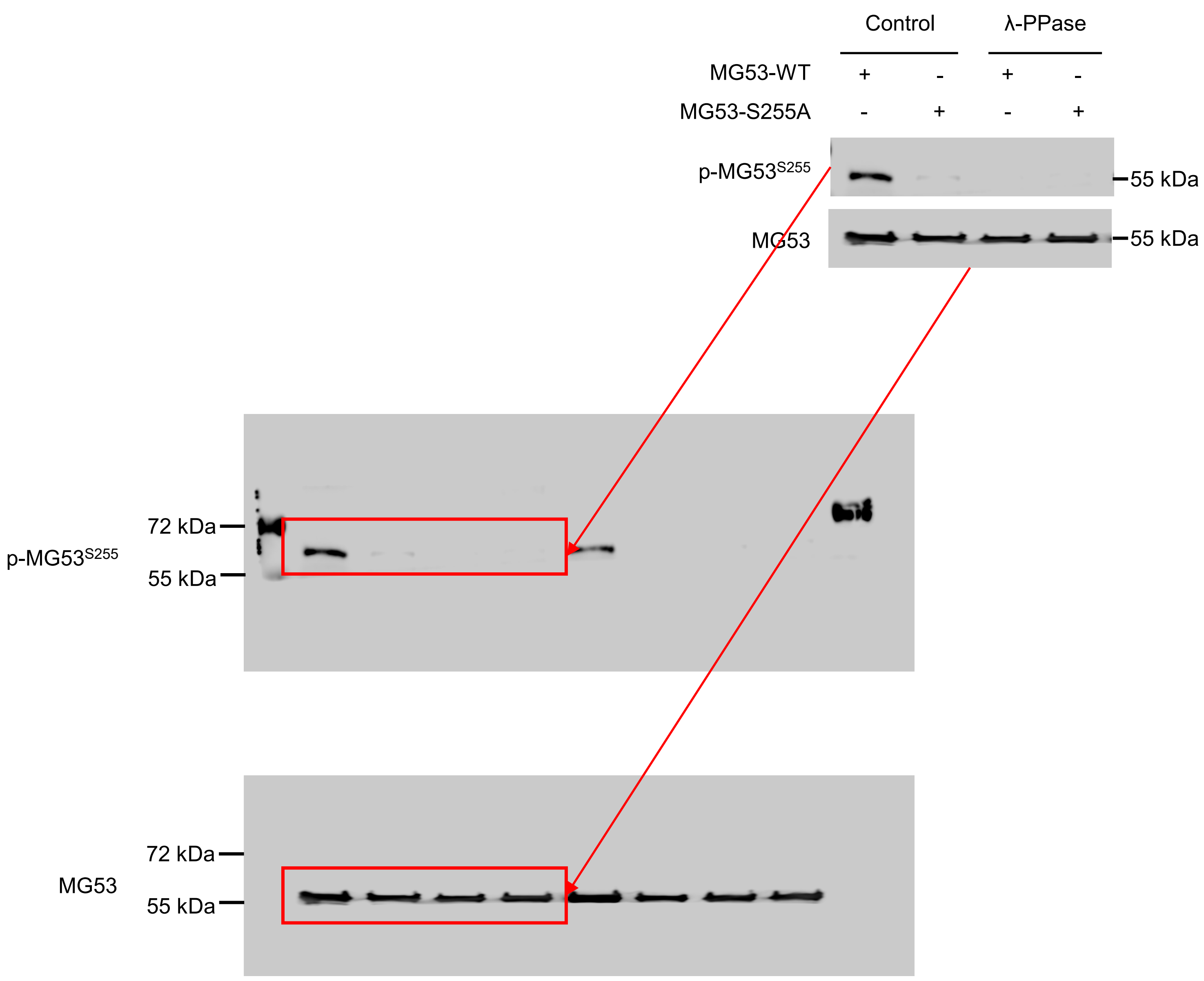

Full unedited gel for Figure S5B

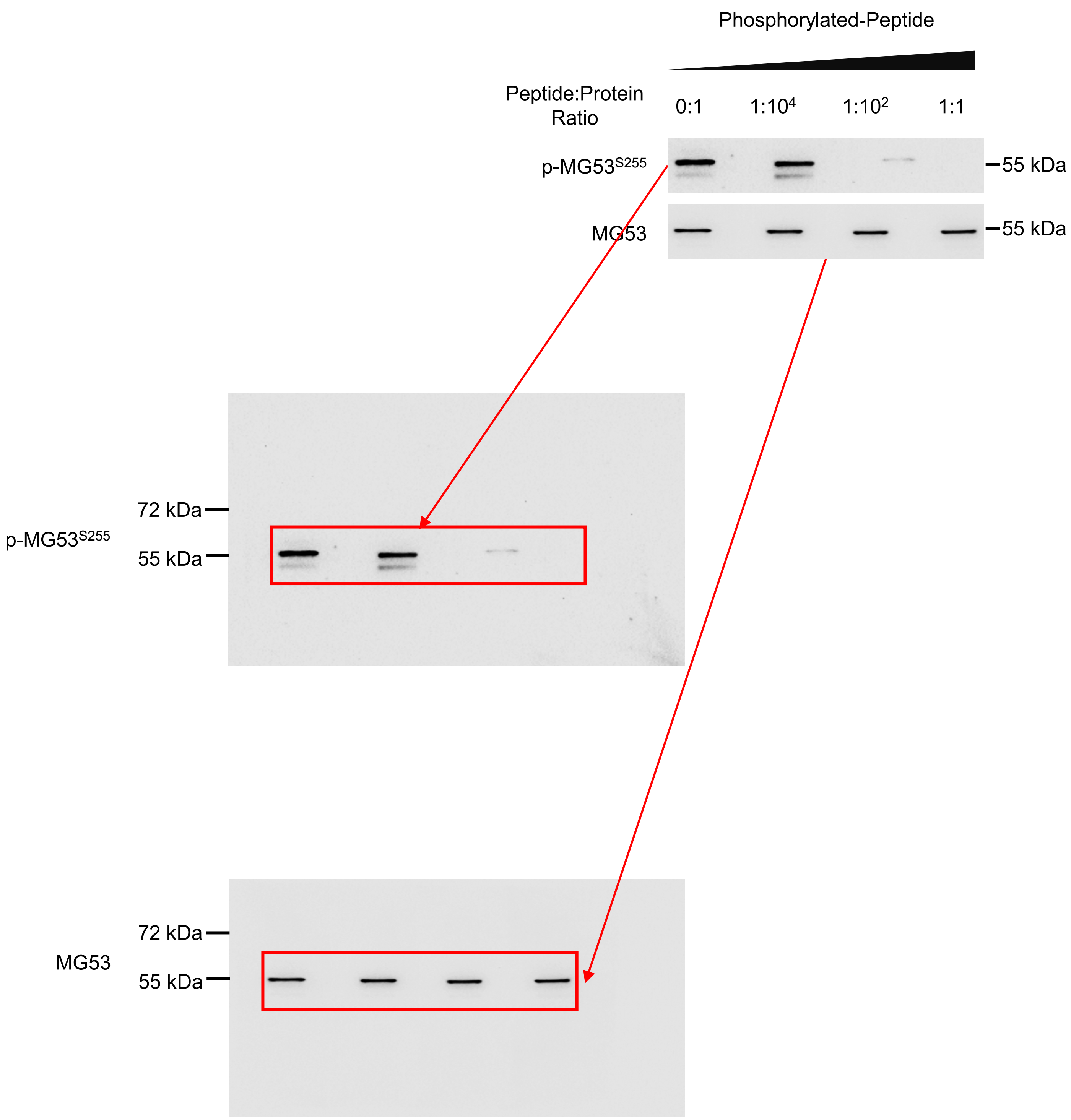

Full unedited gel for Figure S5C

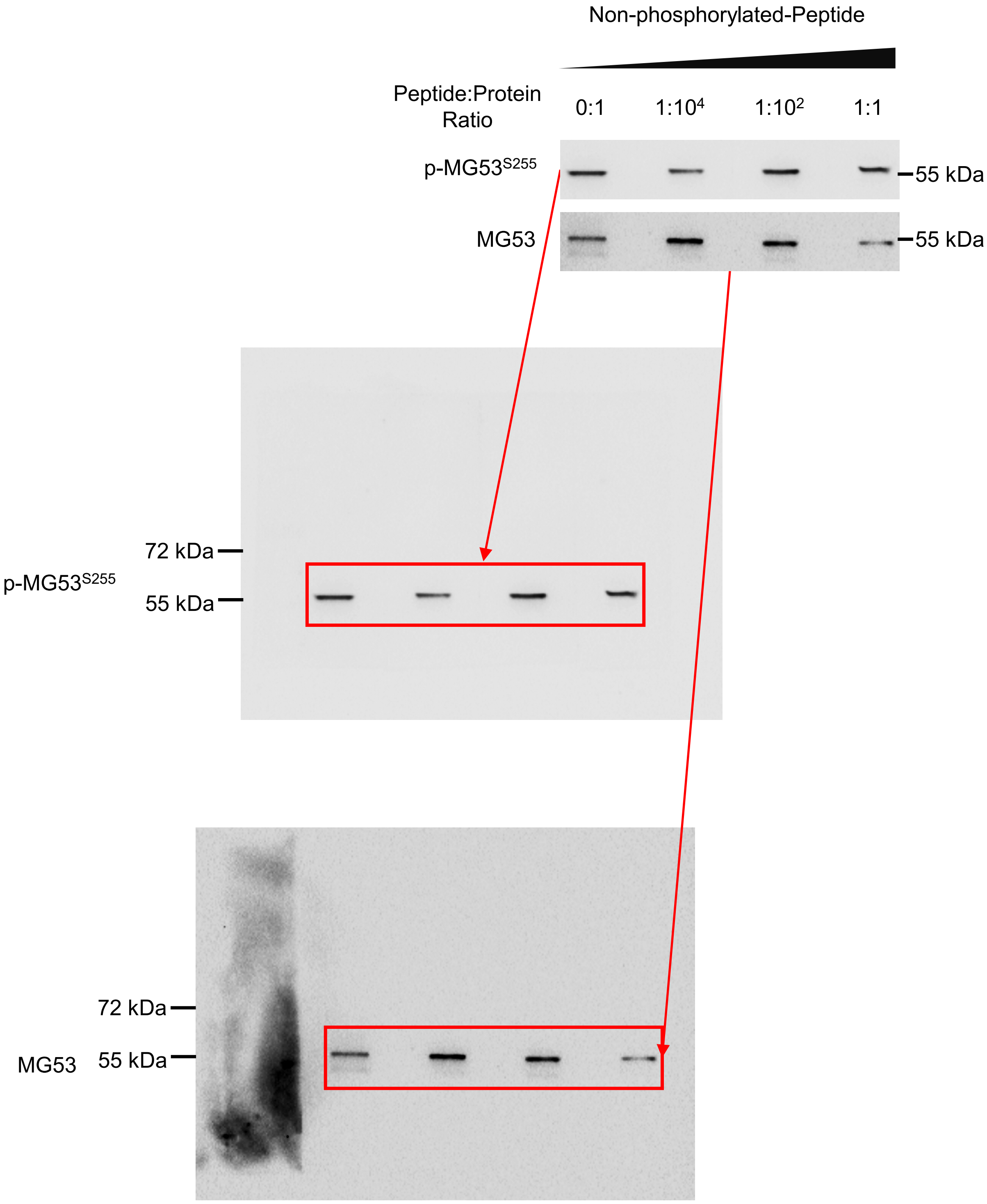

Full unedited gel for Figure S5D

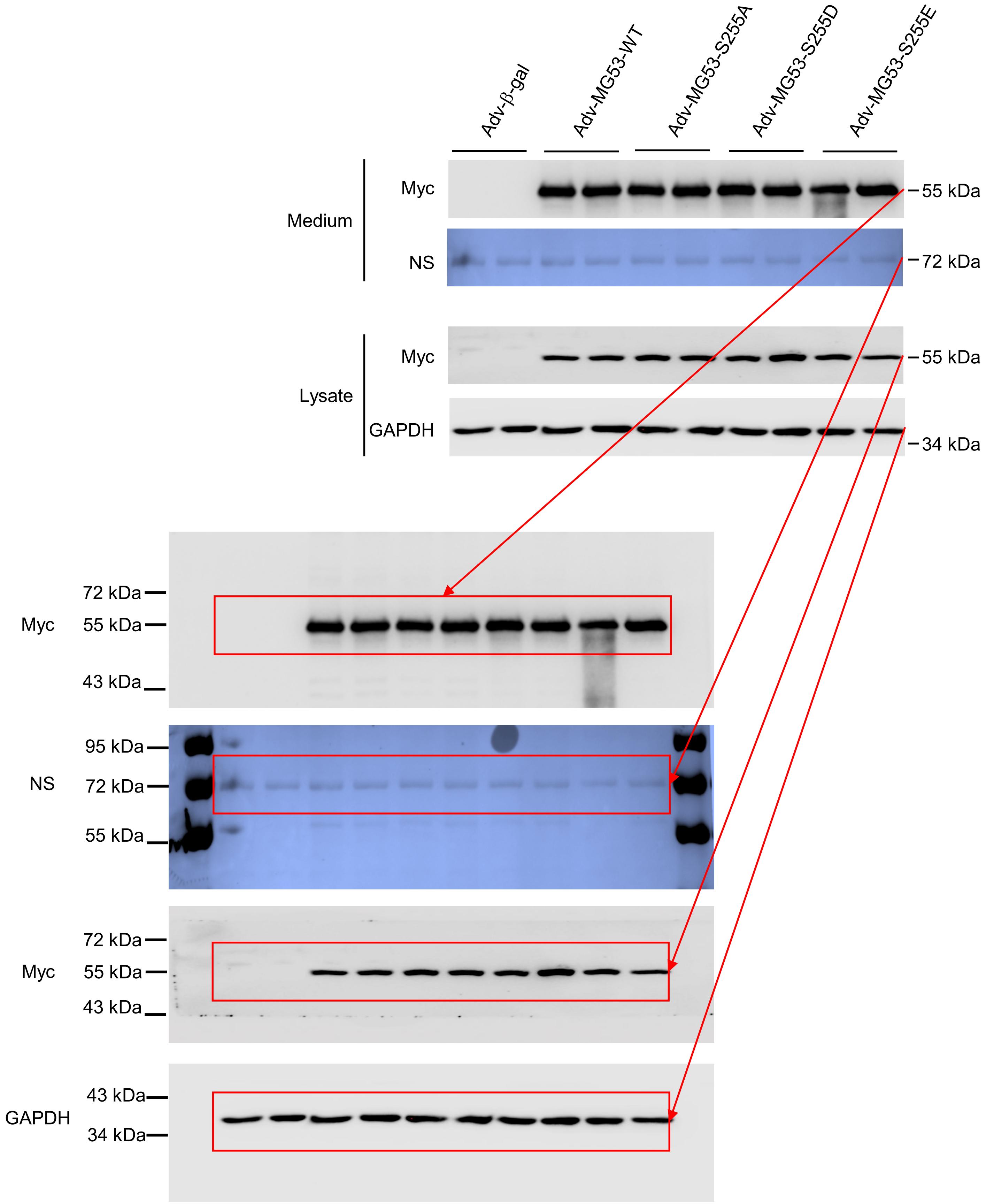

Full unedited gel for Figure S7A

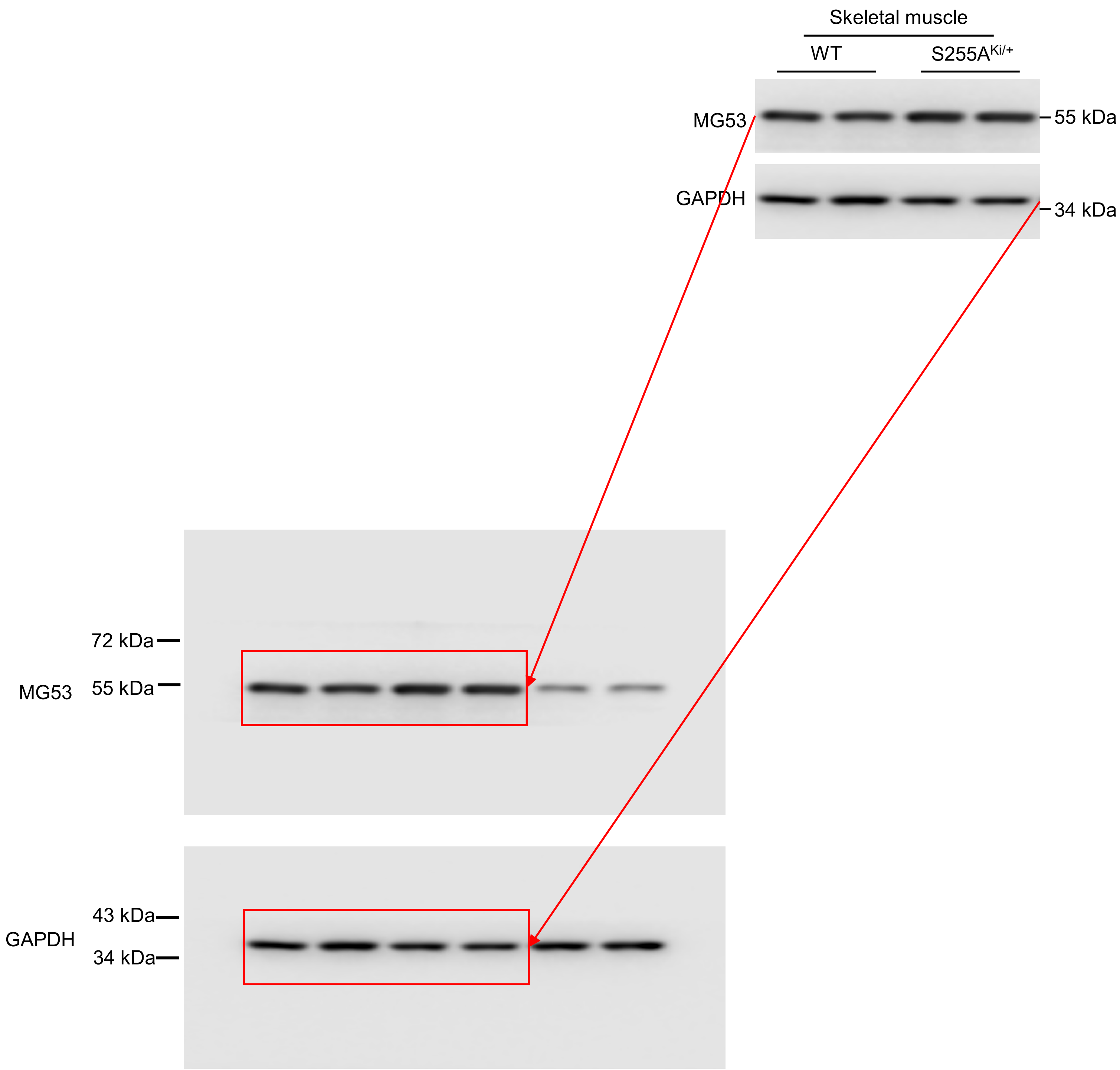

Full unedited gel for Figure S12D
